# An Investigation of Structure–Activity Relationships and Cell Death Mechanisms of the Marine Alkaloids Discorhabdins in Merkel Cell Carcinoma Cells

**DOI:** 10.3390/md21090474

**Published:** 2023-08-29

**Authors:** Maria Orfanoudaki, Emily A. Smith, Natasha T. Hill, Khalid A. Garman, Isaac Brownell, Brent R. Copp, Tanja Grkovic, Curtis J. Henrich

**Affiliations:** 1 Molecular Targets Program, Center for Cancer Research, National Cancer Institute, Frederick, MD 21702, USA; maria.orfanoudaki@nih.gov (M.O.); emily.smith2@nih.gov (E.A.S.); 2 Basic Science Program, Frederick National Laboratory for Cancer Research, Frederick, MD 21702, USA; 3 Dermatology Branch, National Institute of Arthritis and Musculoskeletal and Skin Diseases, Bethesda, MD 20891, USA; natasha.hill@nih.gov (N.T.H.); khalid.garman@nih.gov (K.A.G.); isaac.brownell@nih.gov (I.B.); 4 School of Chemical Sciences, University of Auckland, Auckland 1142, New Zealand; b.copp@auckland.ac.nz; 5 Natural Products Branch, Developmental Therapeutics Program, Division of Cancer Treatment and Diagnosis, National Cancer Institute, Frederick, MD 21702, USA

**Keywords:** Merkel cell carcinoma, discorhabdin, structure–activity relationship, mechanism of action

## Abstract

A library of naturally occurring and semi-synthetic discorhabdins was assessed for their effects on Merkel cell carcinoma (MCC) cell viability. The set included five new natural products and semi-synthetic compounds whose structures were elucidated with NMR, HRMS, and ECD techniques. Several discorhabdins averaged sub-micromolar potency against the MCC cell lines tested and most of the active compounds showed selectivity towards virus-positive MCC cell lines. An investigation of structure–activity relationships resulted in an expanded understanding of the crucial structural features of the discorhabdin scaffold. Mechanistic cell death assays suggested that discorhabdins, unlike many other MCC-active small molecules, do not induce apoptosis, as shown by the lack of caspase activation, annexin V staining, and response to caspase inhibition. Similarly, discorhabdin treatment failed to increase MCC intracellular calcium and ROS levels. In contrast, the rapid loss of cellular reducing potential and mitochondrial membrane potential suggested that discorhabdins induce mitochondrial dysfunction leading to non-apoptotic cell death.

## 1. Introduction

Merkel cell carcinoma (MCC) is a rare, aggressive, and rapidly metastatic neuroendocrine skin cancer [1,2,3,4]. Although rare, the incidence of MCC is increasing [5] due to the aging population, UV exposure [1,2,3,4], and immune suppression [6]. The majority of MCC cases result from chromosomal integration of Merkel cell polyomavirus (MCPyV) and expression of large T antigen and small T antigen [1]. These MCC virus-positive cases (VP-MCC) have disruptions in a variety of cell signaling pathways [1]. The other 20% of MCC cases have mutagenesis induced by UV light damage [1]. These virus-negative cases (VN-MCC) have a higher mutational burden with frequent mutations in oncogenes and tumor suppressor genes [1], and generally have poorer prognosis than VP-MCC patients [7]. The primary treatment of MCC is surgery and radiation for local disease and immune checkpoint inhibitors for metastasis [1,2,3,4,8]. Despite high response rates to immunotherapy, fewer than half of patients have a durable response, and thus, novel effective treatments are needed [9]. Genomic and expression analysis of MCC tumors has suggested a variety of potential therapeutic targets [10,11].

**Figure 1:**
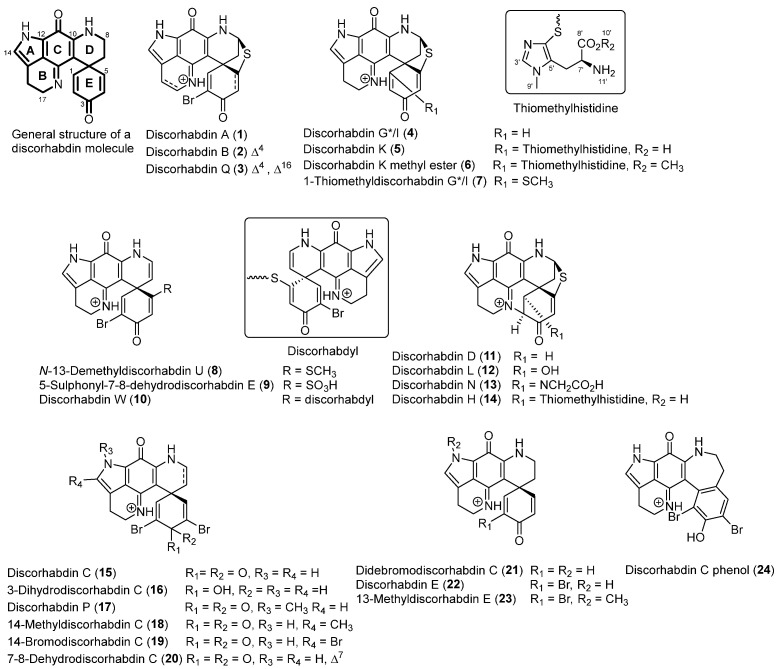
Structures of the naturally occurring and semi-synthetic molecules used in this study.

The ongoing opportunity for development of new MCC-active therapeutics has driven a number of drug discovery efforts over the years, typically based on in vitro MCC cell viability applied to chemical libraries or to small numbers of known compounds and leading to the identification of a range of potential drugs and probes [12,13,14,15,16,17,18,19,20,21]. In particular, a recent high-throughput screening campaign resulted in the identification of a substantial number of synthetic and naturally occurring small molecules as differential modulators of MCC cell viability compared to immortalized skin cells [22]. Among these were natural products not previously demonstrated to be MCC-selective agents, including the terpenes glaucarubin, englerin A, and thapsigargin; the lignan etoposide; naphtoquinone plumbagin, macrolide borrelidin; and the alkaloids discorhabdin D, petrosamine A, mitomycin C, and pluripotin. Discorhabdin D, a natural product isolated from marine sponges [23,24], was one of the most potent and selective of the active compounds identified in the screen. The discorhabdins are a group of marine alkaloids containing a core pyrido [2,3-*h*]pyrrolo [4,3,2-*de*]quinoline tetracyclic skeleton bound to various *spiro*-substituents at the C-6 position (Figure 1). The compounds have been associated with potent cytotoxic, antimicrobial, antiviral, antimalarial, and immunomodulatory activities [23,25]; however, their general toxicity towards mammalian cell lines and lack of selectivity have hampered their progress towards drug development. Recently, several trends on the structure/activity of the discorhabdins have been established—analogues containing the redox-active iminoquinone moiety and the electrophilic spirodienone ring are cytotoxic in the sub micromolar ranges, but these two reactive structural features were hypothesized to be involved in the generation of an oxygen radical and nucleophilic substitution, respectively, which is likely responsible for the broad cytotoxic activity observed for the group [23,26,27]. Compounds which possess an additional ring between C-2 and *N*-18 atoms and substitution at the C-1 position have neither of these reactive structural features and tend to be less cytotoxic to mammalian and cancer cell lines [27].

Investigation of cell death mechanisms of action for cytotoxic compounds is an increasingly important activity in drug discovery and development [28], and cell death mechanisms have been probed for a number of MCC-active compounds. In most cases, induction of apoptosis in the MCC cells was observed [29,30,31,32,33,34,35,36]. Ferroptosis [37,38] and autophagy/necroptosis/autophagic cell death [39] have also been reported as potential mechanisms of action in targeted MCC cell death. On the other hand, relatively little has been reported in the literature regarding molecular targets and mechanisms of action of discorhabdins beyond general toxicity in a variety of cell types. There are a few reports of effects of various discorhabdins on specific molecular targets, including acetylcholinesterases [40], calcineurin [41], caspase-3 [41], heat shock proteins [42], and HIF1α-P300 binding interaction [43] in tumor and angiogenesis models [44]. However, cell death mechanisms have not been extensively investigated for cytotoxic activities of discorhabdins.

In the current study, a large library of discorhabdins was assessed for effects on MCC cell viability. The discorhabdins were sourced from in-house pure compound libraries as well as new natural products isolated and described herein. Investigation of structure–activity relationships (SAR) resulted in the identification of crucial structural features driving the activity and has allowed for an expanded understanding of discorhabdin SAR. Analysis of cell death mechanisms induced in MCC cells by discorhabdins suggested induction of non-apoptotic cell death involving mitochondrial dysfunction in all tested MCC cells. Active discorhabdins induced cell death at higher potency in VP-MCC cell lines compared to VN-MCC lines, but without apparent mechanistic differences.

## 2. Results

The library of twenty-four molecules shown in Figure 1 represents the majority of structural diversity reported for the monomeric discorhabdins. In addition to eighteen known natural products (**1**–**5**, **7**, **10**–**16**, **19**, **21**, **22,**) sourced from pure compound libraries and three previously reported semi-synthetic derivatives (**8**, **17**, and **24**), the set was expanded to include the structures of three new structures, namely, discorhabdin K methyl ester (**6**), 5-sulphonyl-7,8-discorhabdin E (**9**), and 7–8-dehydrodiscorhabdin C (**20**), as well as two new alkylated semi-synthetic discorhabdins, namely, 14-methyl discorhabdin C (**18**) and 13-methyl discorhabdin E (**23**) (Figure 1). A summary of the physicochemical properties of compounds **1**–**24** is presented in Supporting Information (Figure S2) and shows molecular weights ranging from 400 to 700 (with the exception of the dimer discorhabdin W at Mw = 1055), *c*logP ranging from −4 to 3, polar surface area ranging from 60 to 180, hydrogen bond acceptors ranging from 3 to 9, and hydrogen bond donor ranging from 1 to 7, all showing favorable physicochemical properties for drug-like small molecules. The twenty-four structures can be classified into three distinct groups represented by discorhabdins B, C, and D as prototypical compounds. The discorhabdin B series is characterized by the core pentacyclic pyrridopyroloiminoquinone structures **1**–**10** containing a *spiro* ring at C-6 and either a thioether bridge between C-5 and C-8 (**1**–**7**) or a thiol substitution at C-5 (**8**–**10**). The discorhabdin D series (**11**–**14**) is characterized by an additional ring between *N*-18 and C-2 positions and various -*O*, -*N*, and -*S* atom substitutions at C-1. The discorhabdin C series is characterized by pentacyclic pyrridopyroloiminoquinone structures **15**–**24** containing a *spiro* ring at C-6, thioether or sulfur substitution at C-5, and bromine or alkyl substitutions at C-2, C-4, *N*-13, and C-14.

### 2.1. Structure Elucidation

**Figure 2:**
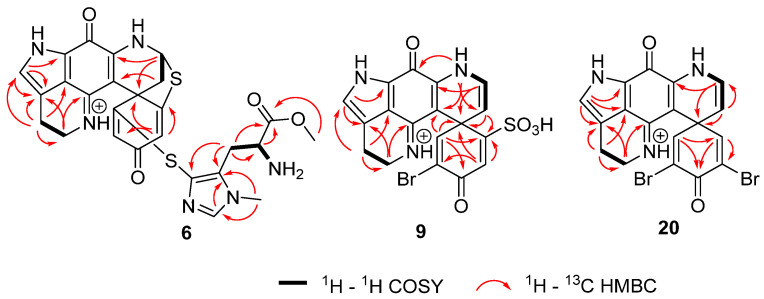
Crucial 2D NMR correlations used to establish the structures of **6**, **9**, and **20**.

Compound **6** was assigned the molecular formula C_26_H_25_N_6_O_4_S_2_ as established by the quasi-molecular ion at *m*/*z* 549.1374 (calculated for C_26_H_25_N_6_O_4_S_2_ 549.1379) observed in HRESIMS. ^1^H and ^13^C NMR data indicated high similarity with the known compound discorhabdin K (**5**) [26]. Additionally, an extra methyl group (*δ*_H_ 3.61, *δ*_C_ 53.0) at the carboxylic acid on the thiohistidine residue of the molecule was observed, supported by lower chemical shifts of the carbonyl group at position C-8′ (*δ*_C_ 168.7) and the methine group at position C-7′ (*δ*_C_ 50.9), as well as a higher chemical shift of proton H-7′ (*δ*_H_ 4.20). Key ^1^H–^13^C HMBC correlations from H-10′ (*δ*_H_ 3.61) to C-8′ (*δ*_C_ 168.7) (Figure 2) confirmed the location of the methyl group. In order to annotate the stereochemical assignment of compound **6,** its experimental ECD spectrum was compared to that of a structurally related discorhabdin with a previously defined configuration, namely (+)-(6*S*,8*S*,7′S*)-discorhabdin K (**5**). The two structures showed high similarity of the ECD spectra (Figure S3) and the same sign of optical rotary dispersion values at three different wavelengths, thereby establishing that the two molecules have the same configuration and identifying compound **6** as (+)-(6*S*,8*S*,7′S*)-discorhabin K methyl ester. In an effort to determine if compound **6** was an isolation procedure artefact of the co-occurring metabolite discorhabdin K (**5**), we heated **6** in methanol [0.1% TFA] as well as methanol-water (1:1) [0.1% TFA] at 40 °C and monitored the stability of the compound over 72 h. These conditions represented an extreme version of the steps involved in the isolation procedure where the fractions were subjected to semi-preparative HPLC using acidified water and methanol and then dried overnight in a centrifugal evaporator at 40 °C. The results showed a small but detectable appearance of an ion at *m*/*z* 549.1374 corresponding to discorhabdin K methyl ester, and the authors conclude that **6** may be an isolation procedure artefact of the natural product **5**.

**Figure 3:**
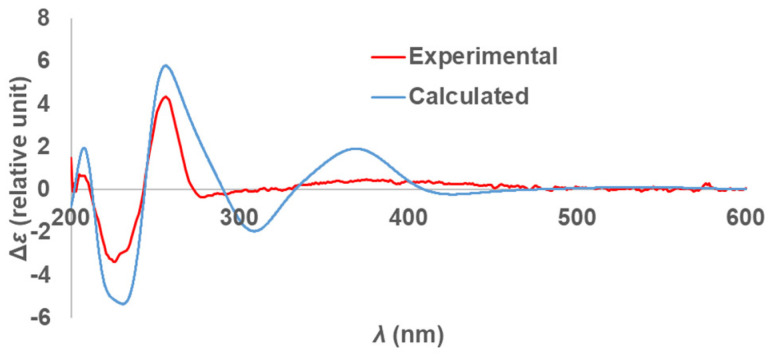
Calculated ECD spectrum (blue line) for compound **9** compared to the experimental spectrum observed for compound **9** (red line).

Compound **9** was assigned the molecular formula C_18_H_13_BrN_3_O_5_S as established from the quasi-molecular ions at *m*/*z* 461.9751 (calculated for C_18_H_13_^79^BrN_3_O_5_S, 461.9754) and 463.9730 (calculated for C_18_H_13_^81^BrN_3_O_5_S, 463.9734) observed in HRESIMS spectra. ^1^H and ^13^C NMR data indicated high similarity with the known semisynthetic compound *N*-13-demethyldiscorhabdin U (**8**) [45], with one major difference at the substitution of C-5. The existence of the sulfonyl group at C-5 was indicated by two IR characteristic bands at 1210 and 1137 cm^−1^ [46,47] and a loss of *m*/*z* 79.9567 (negative mode) in the high-resolution product ion spectra (Figure S4). The sulphonyl group while relatively rare in nature, has been reported in metabolites from both marine and terrestrial organisms, for example in nakijiquinone R isolated from marine sponges of the family Spongiidae [48], in the triterpene glycoside koreoside A isolated from the sea cucumber *Cucumaria koraiensis* [49], and in diterpenoid alkaloids from *Aconitum carmichaelii* [50]. In order to complete the stereochemical assignment of compound **9**, the experimental ECD spectrum of the natural product was compared to the calculated ECD spectrum of the 6*S* enantiomer of **9** using TDDFT calculations. A good agreement between the measured and calculated ECD spectra of **9** (Table S1, Figure 3) was supportive of the *S* configuration at C-6 to complete the structure of (+)-(6*S*)-5-sulphonyl-7,8-dehydro-discorhabdin E. Additionally, here for the first time, the absolute configuration of discorhabdin E (**22**) has been established to be 6*S* by comparison of the experimental and calculated ECD spectra (Table S2, Figure S1).

Compound **20** was assigned the molecular formula C_18_H_12_Br_2_N_3_O_2_ as established from the quasi-molecular ions at *m*/*z* 459.930 (calculated for C_18_H_12_^79^Br_2_N_3_O_2_, 459.930), 461.9276 (calculated for C_18_H_12_^79^Br^81^BrN_3_O_2_, 461.9271), and 463.9260 (calculated for C_18_H_12_^81^Br_2_N_3_O_2_, 463.9250) observed in HRESIMS spectra. ^1^H and ^13^C NMR data had high similarity with the known compound discorhabdin C (**15**) [51]. However, one difference could be observed, the presence of an additional olefin at dihydropyridine ring between C-7 and C-8 which was indicated by higher chemical shifts of position C-7 (*δ*_H_ 4.71, *δ*_C_ 109.8) and position C-8 (*δ*_H_ 6.53, *δ*_C_ 125.6) and were consistent with literature values [52,53,54]. ^1^H–^1^H COSY correlations of the protons H-7/H-8/H-9 and ^1^H–^13^C HMBC correlations from H-7 (*δ*_H_ 4.71) to C-20 at *δ*_C_ 95.4 and H-8 (*δ*_H_ 6.53) to C-6, C-7, and C-10, (*δ*_C_ 47.1, 109.8 and 145.2) (Figure 2) confirmed the placement of the double bond and completed the structure of compound **20** which was assigned the trivial name 7,8-dehydro-discorhabdin C.

Additionally, two semisynthetic compounds were made: 14-methyldiscorhabdin C (**18**) and (+)-(6*S*)-*N*-13-methyldiscorhabdin E (**23**). 14-Methyldiscorhabdin C (**18**) was prepared with the addition of FeCl_2_ and H_2_O_2_ in a DMSO solution of discorhabdin C based on the protocol described in Zhang et al., [55] which resulted in the addition of a methyl group at C-14 of discorhabdin C. The position of the extra methyl group was indicated by specific chemical shifts of the methyl group (*δ*_H_ 2.25, *δ*_C_ 11.1) and ^1^H–^13^C HMBC correlations from H-22 (*δ*_H_ 2.25) to C-15 at *δ*_C_ 118.1 and C-14 at *δ*_C_ 139.3. (+)-(6*S*)-*N*-13-Methyldiscorhabdin E (**23**) was prepared using a previously established semi-synthetic route [45] which included the addition of CH_3_I and K_2_CO_3_ to an acetone solution of discorhabdin E and resulted in the addition of a methyl group at position C-13 of discorhabdin E. The placement of the extra methyl group was indicated by specific chemical shifts at *δ*_H_ 3.92, *δ*_C_ 36.5 and ^1^H–^13^C HMBC correlations from H-22 (*δ*_H_ 3.92) to C-12 at *δ*_C_ 124.8 and C-14 at *δ*_C_ 132.0 for compound **23**.

### 2.2. Assay Activity

The discorhabdins were assessed for activity against seven cell lines: three virus-negative (VN-MCC) cell lines (MCC13, MCC26, and UISO), three virus-positive (VP-MCC) cell lines (MKL-1, MKL-2, and Waga), and one non-cancerous skin cell line used as a control (HaCaT). Dose–response curves, discorhabdin structures, and IC_50_ values for each cell line/discorhabdin pair are shown in Figure S39. Activity of the discorhabdins against the MCC cell lines ranged from low nano molar values to greater than 10 µM (highest concentration tested). Eight discorhabdins averaged sub-micromolar potency (IC_50_) across all MCC cell lines tested. These were (in decreasing order of potency) discorhabdin A (**1**), discorhabdin B (**2**), N-13-demethyldiscorhabdin U (**8**), discorhabdin P (**17**), 14-bromodiscorhabdin discorhabdin C (**19**), discorhabdin L (**12**), discorhabdin E (**22**), and discorhabdin G*/I (**4**). Discorhabdins A (**1**), B (**2**), and N-13-demethyl U (**8**) also had IC_50_ values at or below 1 µM for control cells. Five compounds were essentially inactive (>5 µM average IC_50_) against all the cell lines: discorhabdin K (**5**), didebromodiscorhabdin C (**21**), 7-8-dehydrodiscorhabdin C (**20**), discorhabdin H (**14**), and 5-sulphonyl-7,8 dehydrodiscorhabdin E (**9**).

**Figure 4:**
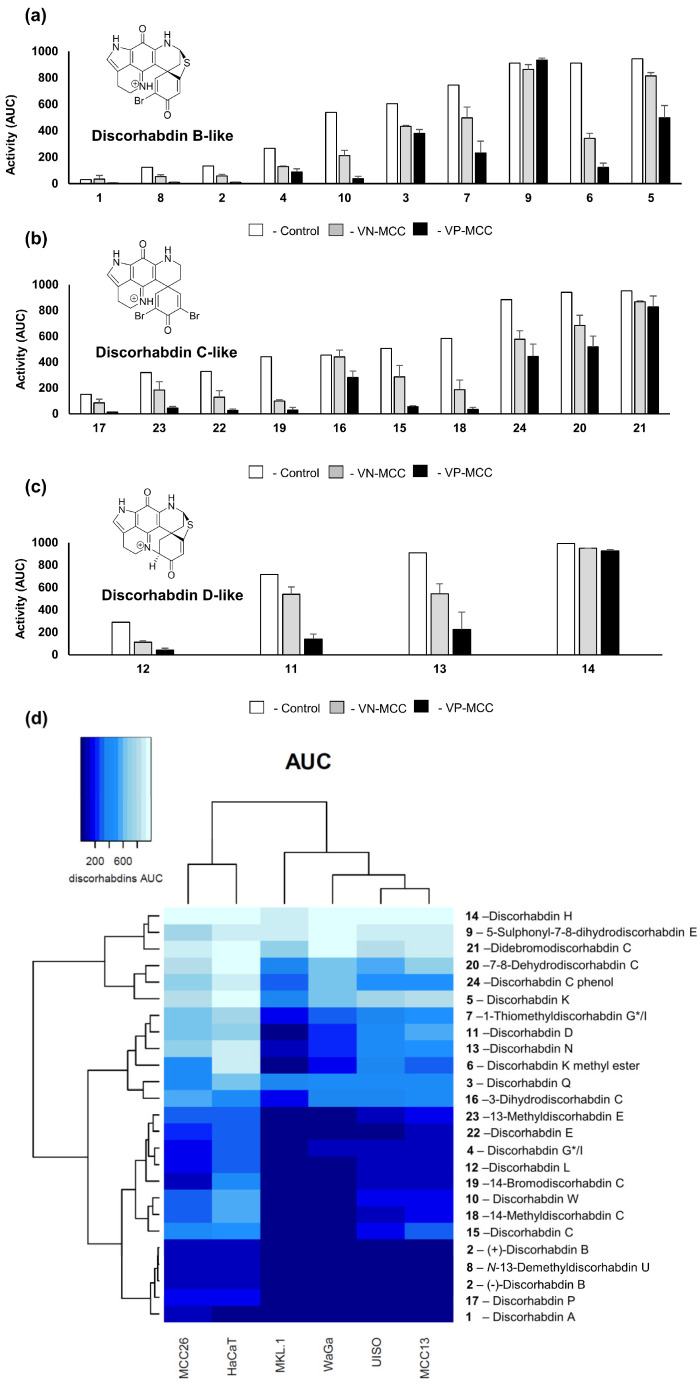
(**a**–**c**) Effects of the discorhabdins on MCC cell viability. Cytotoxicity data are presented from most active (lowest AUC) to least active (highest AUC) based on activity against control cells. VN-MCC and VP-MCC data are averages across each class of cell lines. (AUC, arbitrary units—see Table S3). For VN-MCC and VP-MCC cells, error bars represent se (*n* = 3) or range. Data for discorhabdin B-like structures **1**–**10** is shown in panel (**a**); data for discorhabdin D-like structures **12**–**14** is shown in panel (**b**); and data for discorhabdin C-like structures **17**–**21** is shown in panel (**c**). (**d**) Heat map of the discorhabdin cytotoxicity data organized by inhibitory effect by AUC where darker regions indicate higher activity of each discorhabdin on VN-MCC (MCC13, MCC26, and UISO), VP-MCC (MKL-1, WaGa), and HaCaT control cells. The heatmap was generated in R version 1.3.1073 (R Core Team (2013). R: A language and environment for statistical computing, Vienna, Austria, http://www.R-project.org/ (accessed on 12 January 2023). Dendrograms indicate cell line and compound similarities based on unsupervised hierarchical clustering. Darker shades indicate higher activity.

In order to compare and visualize differences in the activity of the compounds, all of the dose–response curves used identical dilution series, and area under the curve (AUC) was determined for each cell line/discorhabdin pair (Table S3). AUC is a parameter that incorporates potency and efficacy and is useful for comparing activity across multiple cell lines and drugs [56]. The data showed a very high linear correlation between IC_50_ and AUC (R^2^ = 0.98, Figure S40). Subsequent analyses generally utilized AUC, since comparisons could also then include compounds with minimal effect on specific cell lines at the concentrations tested. Figure 4 shows the effects of the discorhabdin alkaloids on Merkel cell viability using AUC and sorted into discorhabdin structural classes. As expected, the AUC data led to similar general conclusions as noted above from IC_50_ data. Figure S41 details selectivity among VN-MCC and VP-MCC cells compared to the control. Interestingly, only 14-bromo discorhabdin C (**19**) and 14-methyl discorhabdin C (**18**) showed substantially increased activity against VN-MCC compared to control cells (>3-fold difference). In contrast, seven discorhabdins had >10-fold increased activity against VP-MCC compared to the control—in order of decreasing relative activity: 14-methyldiscorhabdin C (**18**), 14-bromodiscorhabdin C (**19**), discorhabdin B (**2**), discorhabdin W (**10**), discorhabdin E (**22**), N-13-demethyldiscorhabdin U (**8**), and discorhabdin P (**17**).

Among the discorhabdin B-like structures **1**–**10** (Figure 4a), the most potent compounds were discorhabdins A (**1**), *N*-13 demethyl U (**8**), and B (**2**), which all had the α-bromo enone moiety, suggesting, as others have found [57], that increased cytotoxicity is potentiated by electrophilic reactivity. However, the C-5 sulphonyl-substituted α-bromo enone **9** was practically inactive, while the C-5 thiomethyl-substituted compound **8** was one of the most active compounds tested, suggesting the size and polarity of the C-5 substituent on the discorhabdin B-like scaffold is also crucial for the activity. Discorhabdin B (**2**) was >10-fold more active than discorhabdin Q (**3**) against MCC cells. Discorhabdin Q (**3**) has additional oxidation between C-16 and C-17 as compared to **2,** suggesting that unsaturation on ring B has a deleterious effect on cytotoxicity possibly because of the unstable nature of this metabolite, which may degrade to complex mixtures [57]. Discorhabdin G*/I (**4**) lacking the bromine substitution at C-2 showed a loss of activity compared to compound **2** (compound **2** was 3-fold more active against MCC cells compared to **3**), and substitution at C-1 further decreased activity of the scaffold, in particular the addition of a large thiohistidine group. Interestingly, the presence of the methoxy group in **6** drastically improved the selectivity compared to the free acid group on the thiohistidine in compound **5**. As the discorhabdins are known to occur in enantiomeric configurations, both forms of discorhabdin B were tested. It was found that (+)-**2** and (−)-**2** had comparable activity against MCC and control cell lines.

In the discorhabdin D-like series **11**–**14** (Figure 4b), the presence of an additional ring between C-2 and *N*-18 positions generally reduced potency compared to the discorhabdin B and C analogues. The size of the C-1 substituents had a drastic effect on the potency, with the hydroxy substituted discorhabdin L (**12**) being most active while the glycine and thiohistidine analogues showed a loss of activity. Among the discorhabdin C-like structures **15**–**24** (Figure 4c), the crucial features that enhanced the activity of discorhabdin C were *N*-13 methylation, as seen in compounds **17** and **23**, and the presence of an α-bromo enone moiety. The mono-brominated discorhabdin E (**22**) had comparable activity to the 14-bromodiscorhabdin C (**15**) as well as discorhabdin C (**15**), suggesting that a bromine substitution at positions C-4 and C-14 is not essential for the activity. Additionally, changes in the conjugation of the spirodienone ring E and addition of a C-7 and C-8 olefin had detrimental effects for all cell lines tested, as shown by the results for discorhabdin C phenol (**24**), 3-dihydrodiscorhabdin C (**16**), and 7,8 dehydrodiscorhabdin C (**20**).

### 2.3. Cell Death Mechanisms

**Figure 5:**
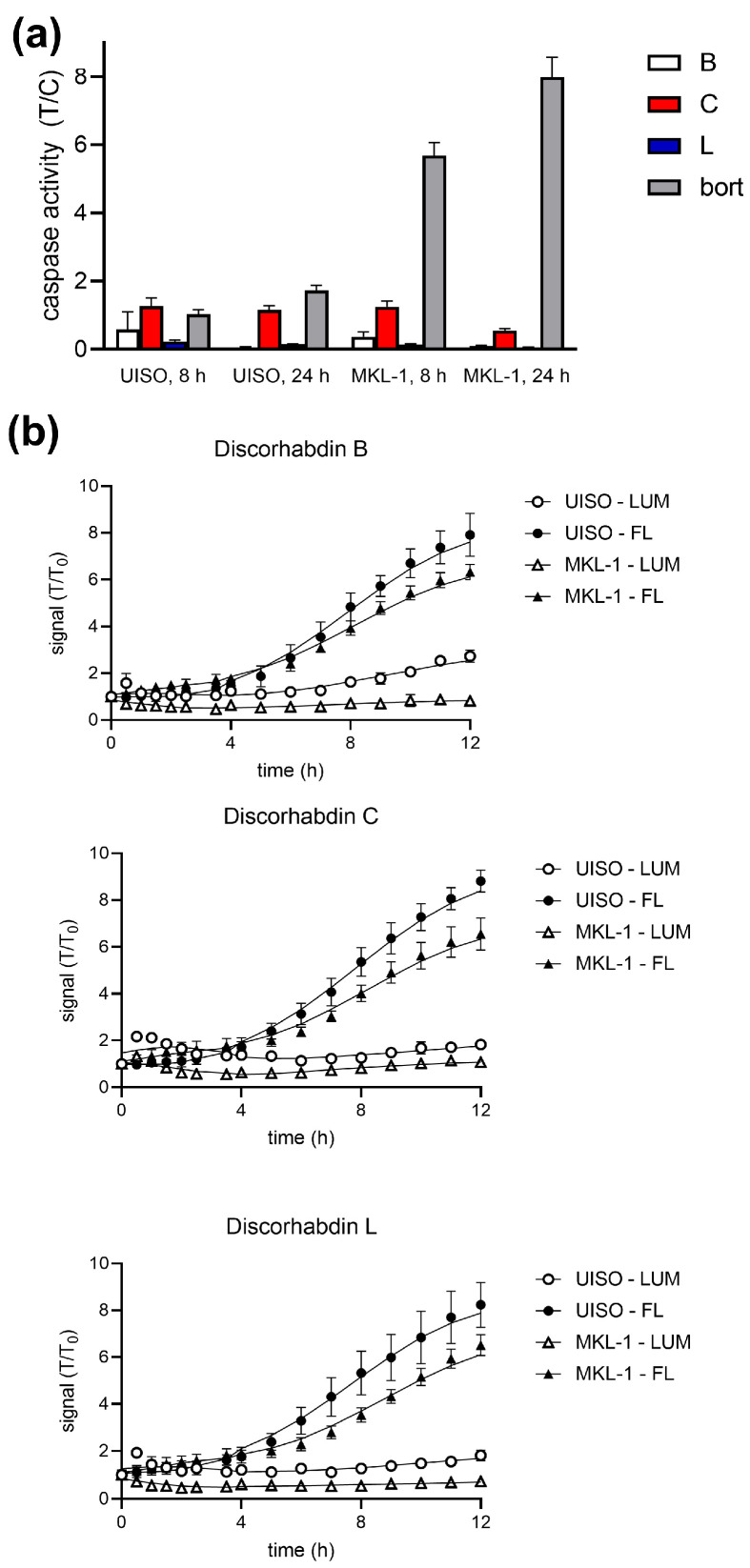
Representative data for effects of pentacyclic (C), hexacyclic (B), and heptacyclic (L) discorhabdins on UISO (VN) and MKL-1 (VP) MCC cells, bort: bortezomib. (**a**) Caspase activation. Cells were treated for 8 or 24 h with indicated discorhabdins or bortezomib. Caspase-3/7 activity was determined (Promega CaspaseGLO-3/7) and normalized to untreated (DMSO) control cells. Error bars represent sd (*n* = 4). (**b**). Apoptosis/necrosis. Cells were treated for up to 24 h with the indicated discorhabdins and assessed for Annexin binding and cell permeability using the Promega RealTime-Glo™ Annexin V Apoptosis and Necrosis Assay kit. Luminescence indicates Annexin binding, fluorescence indicates membrane permeability. Values were normalized to 0 time control. Error bars represent sd (*n* = 4). *p* < 0.01 (vs. DMSO control) for all fluor points at ≥ 6 h, insignificant for luminescence.

Apoptotic cell death was first investigated in MCC cells in response to discorhabdins. Three methods were utilized: activation of effector caspases 3 and/or 7, annexin V binding, and effects of caspase inhibitors. The data in Figure 5a demonstrates lack of caspase-3/7 activation by discorhabdins B (**2**), C (**15**), and L (**12**) against UISO (representative VN-MCC) and MKL-1 (representative of VP-MCC) cells. By comparison, bortezomib treatment robustly activates caspase in these cells (Figure S42). As reported in the literature [39], appreciable caspase activation in MCC cells by bortezomib took up to 24 h. Similarly, discorhabdin treatment did not significantly increase binding of labeled annexin V (Figure 5b, open symbols). Figures S42 and S43 include caspase activation and annexin V-binding/cell permeability results for other discorhabdins and additional MCC cell lines. Pretreatment of cells with the pan-caspase inhibitor Z-VAD-FMK reduced bortezomib-induced, but not discorhabdin-induced MCC cell death (Figure S44). By contrast, rapid increases in cell permeability were observed for discorhabdin-treated MCC cells as measured by accessibility of a cell-impermeant fluorescent DNA-binding compound (closed symbols, Figure 5b). The magnitude of the increase in the fluorescence (i.e., necrotic) signal roughly correlates with the potency of individual discorhabdins against each cell line (Figure S43). Together, these results suggest that discorhabdins induce necrotic rather than apoptotic cell death in MCC cells.

### 2.4. Mitochondrial Dysfunction

**Figure 6:**
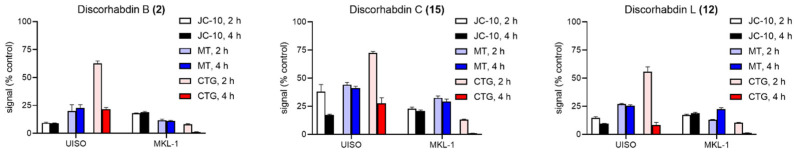
Mitochondrial effects of discorhabdins.

Three aspects of mitochondrial function, mitochondrial membrane potential (MMP), cell reducing potential, and cellular ATP content were investigated (Figure 6). MMP was significantly reduced in UISO and MKL-1 cells after treatment with discorhabdins B (**2**), C (**15**), and L (**12**). Cellular reducing potential also dropped significantly. Similarly, loss of cellular ATP content was dramatic and rapid. The drop in cellular reducing potential was faster and more dramatic than the increase in cell permeability (compare Figure S45 and Figure 5b). The effects of discorhabdins on all three measures of mitochondrial function generally correlated with discorhabdin potency (Figures S45 and S46). As with caspase, the effects of discorhabdins on mitochondrial function were dramatically different to the effects of the apoptotic drug bortezomib (Figures S45 and S46). Induction of mitochondrial dysfunction is often caused by, accompanied by, or followed by generation of reactive oxygen species (ROS) and/or increasing intracellular Ca^2+^. ROS generation and calcium mobilization in MCC cells in response to discorhabdins were assessed using DCFDA and Fluo-4, respectively. None of the discorhabdins tested induced ROS generation or Ca^2+^ mobilization in any of the five MCC cell lines tested although appropriate controls were active in all five cell lines (see Figures S47 and S48, respectively).

Cells were treated with the indicated discorhabdins (10 µM) and assessed for mitochondrial membrane potential (MMP) using JC-10 ratiometric dye. JC-10 was added for the last hour of incubation. Cellular reducing potential was assessed using a RealTimeGlo MT Cell Viability kit, and cellular ATP levels using CellTiterGlo. All values were normalized to DMSO control. Error bars (*n* = 3–4) represent sd. See Figures S45 and S46 for additional cell line/discorhabdin combinations, concentrations, and time points for reducing potential and MMP, respectively.

## 3. Discussion

**Figure 7:**
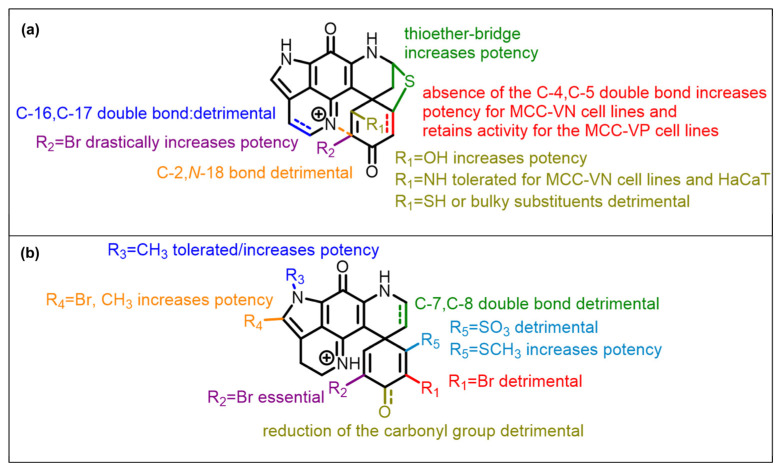
Discorhabdin SAR summary: (**a**) discorhabdin B and L series, (**b**) discorhabdin C series.

The discorhabdin SAR confirmed many of the previously established trends for this group of natural products [27,57,58]. Compounds containing an α-bromo enone moiety were the most potent, while analogues with an additional bridge between C-2 and *N*-14 were less active. Large, bulky substituents such as thiohistidine on position C-1 in both discorhabdin B-type or D-type structures had a detrimental effect on the activity. Changes in the *spiro*-dienone ring E, such as reduction at C-3 or rearrangement to a phenol, had detrimental effects on the activity. Here, we have expanded the discorhabdin SAR (Figure 7) to show that, in MCC cells, methylation of the pyrrole ring A enhances the activity, as does the presence of a thioether ring between C-5 and C-8 positions. Addition of an olefin between C-7 and C-8 was found to have a detrimental effect on the activity of the group, as does the addition of large polar substituents on C-5.

Several pairs of the discorhabdins used in this study may also give some insights into features relevant to effects on cell viability and/or selectivity for MCC-VP cells. Discorhabdin A (**1**) differs from the rest of the discorhabdin B series in that it lacks the C-4–C-5 olefin. It was the most generally toxic compound tested and considerably less selective. Discorhabdin P (**17**) was previously reported to be considerably less cytotoxic than discorhabdin C (**15**) [45,58], whereas, in the present work, it was more potent than discorhabdin C in all seven cell lines, albeit with very similar VP-MCC selectivity. With regard to bromination of the spirodienone ring, discorhabdin G*/I (**4**) had similar potency, but lost selectivity by comparison to discorhabdin B (**2**).

There is very little in the literature regarding cell death mechanisms in response to discorhabdins in susceptible cells. One of the more extensive mechanistic studies involved analysis of discorhabdin A and semi-synthetic discorhabdin analogs. No effects on kinases, HDACs, telomerase, or proteasome were found. NCI-60 cell analysis provided no significant insights [59]. Discorhabdin P has been reported to inhibit caspase-3 [41]. Given this paucity of information related to discorhabdins cell death mechanisms were investigated for discorhabdins and MCC cells.

A range of regulated cell death mechanisms have been described and extensively reviewed in the literature [28,60]. Several of these have been implicated in drug-induced MCC cell death. As noted, many drugs have been reported to induce apoptotic cell death in MCC. Based on multiple assessments of apoptotic cell death (caspase 3/7 activation, annexin V binding, caspase inhibitor effects, and cell permeability), it seems clear that discorhabdin-induced loss of cell viability is not due to the induction of apoptosis. Comparisons to effects of bortezomib, known to induce apoptosis in MCC cells [39], further corroborate this conclusion. Mitochondria are critical regulators of a number of cell death modalities, including apoptosis [2,61]. It has also long been known that oxidative stress and increased intracellular calcium can induce apoptotic cell death [3,62]. However, neither ROS generation nor increased intracellular [Ca^2+^] were observed in discorhabdin-treated MCC cells.

The characteristics of discorhabdin-induced MCC cell death are somewhat consistent with caspase-independent necroptotic cell death [63]. However, pre-treatment of MCC cells with necrostatin-1 did not block discorhabdin effects suggesting a non-necroptotic mechanism [64]. Furthermore, necroptosis and ROS generation typically correlate [65], but ROS was not detected in response to discorhabdins. Examples of necroptosis-inducing drugs described in the literature include aurora kinase inhibitors [63]. Interestingly, aurora kinase inhibition has recently been reported to affect MCC, albeit by induction of apoptosis rather than necroptosis [32].

Ferroptosis is another caspase-independent cell death mechanism observed in drug-treated MCC [37], but is also generally ROS-dependent [66]. Autophagy/autophagic cell death has been observed in drug-treated MCC cells [38,39]. Pretreatment of cells with autophagy inhibitors [67] MRT68921, bafilomycin, or chloroquine did not block discorhabdin-induced MCC cell death. LC3-II, a common marker of autophagy, was not detected by Western blot analysis of discorhabdin-treated cells. Similarly, pretreatment with the commonly used parthanatos inhibitor DPQ [68] did not block the effects of discorhabdins on MCC cells.

Discorhabdin-induced cell death appeared to be equivalent for all of the discorhabdins tested and for both VN-MCC and VP-MCC cell lines. No significant differential other than potency was observed with regard to discorhabdin type, target cells, or cell death mechanistic assays. These results suggest that the effects of discorhabdins on MCC cells are due to the induction of mitochondrial dysfunction. However, the lack of differential activities or mechanisms between classes of discorhabdins suggests that discorhabdin electrophilic reactivity with mitochondrial or other cellular targets may not be the driving factor in their effects on Merkel cell carcinoma cells. The difference in susceptibility between VN-MCC and VP-MCC cells also appears to be subtle. On the other hand, the marked increased activity of discorhabdins against VP-MCC cells may be important. Although VP-MCC disease tends to have a better prognosis, MCPyV, in particular small T antigen, has been reported to enhance metastatic potential [69].

Discorhabdins B, C, and L are highlighted as representative of the three main structural classes of discorhabdins. Discorhabdin L has also been highlighted in this work in part because it is one of the few (if not the only) discorhabdins that has been shown to have minimal in vivo toxicity in animals, in this case in a prostate cancer xenograft model [44]. Further development of this discorhabdin for application in prostate cancer may allow for parallel development for targeting MCC.

## 4. Materials and Methods

### 4.1. General Experimental Procedures

Optical rotations were measured on a Rudolph research analytical AUTOPOL IV automatic polarimeter (Rudolph Research Analytical, Hackettstown, NJ, USA) using a cell of 0.25 dm pathlength and MeOH as the solvent at 20 °C, ECD experiments were recorded on a J-1500 CD spectrophotometer (JASCO Inc., Easton, MD, USA). IR spectra were recorded with a Bruker ALPHA II FT-IR spectrometer (Bruker, Billerica, MA, USA), and UV spectra were measured with an HP 8453 UV-VIS spectrophotometer (Agilent Technologies, Palo Alto, CA, USA) and a Varian Cary 50 Bio spectrophotometer (Agilent, Santa Clara, CA, USA). HPLC separations were performed on a Gilson HPLC system (Middleton, WI, USA) equipped with a 322 pump, a 172-diode array detector, and a GX-281 liquid handler. Samples were dried on a Thermo Savant Explorer-220 speed vacuum system and a SP Genevac vacuum system (SP, Warminster, PA, USA). NMR spectra were obtained with either a 600 MHz Bruker Avance III NMR spectrometer (Bruker, Billerica, MA, USA) equipped with a 3 mm cryogenic probe or a 600 MHz Bruker Avance III HD spectrometer (Bruker, Billerica, MA, USA), equipped with a triple resonance 5 mm CPP TCI cryo-probe, both operating at a frequency of 600.0 MHz for the ^1^H nucleus and 150.9 MHz for the ^13^C nucleus. Spectra were calibrated to residual solvent signals at δ_H_ 2.50 and δ_C_ 39.5 (DMSO-*d*_6_). All 2D NMR experiments were acquired with non-uniform sampling (NUS) set to 50% or 25%. HMBC experiments were run with ^n^*J*_CH_ = 8.0 Hz. HRESIMS data were acquired on a 6230 Accurate-Mass TOF LC/MS system (1260 Infinity II) equipped with a dual AJS ESI source and a 6545 Accurate-mass Q-TOF LC/MS (1260 Infinity II) (Agilent Technologies, Santa Clara, CA, USA), whereas low-resolution mass spectra were measured with an Agilent InfinityLab LC/MSD System comprising an Agilent HPLC 1260 HPLC, equipped with a binary pump, autosampler, column oven, and photodiode array detector Agilent 1260 HPLC (Santa Clara, CA, USA).

### 4.2. Chemicals and Reagents

All chemicals were purchased from Sigma Aldrich (St. Louis, MO, USA) and Merck KGaA (Darmstadt, Germany). All solvents required for isolation and analytical experiments were purchased from Sigma Aldrich (St. Louis, MO, USA). The isolated compounds were dissolved in deuterated DMSO-*d*_6_ and methanol-*d*_4_ from Cambridge Isotope Laboratories (Tewksbury, MA, USA). Ultrapure water was produced with a Hydro^®^ (Hydro, NC, USA) purification system. HP-20 SS, Sephadex LH-20, and C18 silica (55–105 µm, 125 Å) material were purchased from Sigma-Aldrich (St. Louis, MO, USA), GE Healthcare (Chicago, IL, USA), and Waters (Milford, MA, USA), respectively.

### 4.3. Natural Product Isolation

Compound **1**: (+)-(5*R*,6*S*,8*S*) discorhabdin A was isolated from *Latrunculia brevis*, collection and isolation details of which have previously been reported [43].

Compound **2**: (+)-(6*S*,8*S*) and (−)-(6*R*,8*R*) discorhabdins B were isolated from *Latrunculia* sp., collection and isolation details of which have previously been reported [70].

Compound **3**: (+)-(6*S*,8*S*) discorhabdin Q was isolated from *Latrunculia purpurea*, collection and isolation details of which have previously been reported [71].

Compound **4**: (+)-(6*S*,8*S*) discorhabdin G*/I was isolated from *Latrunculia brevis*, collection and isolation details of which have previously been reported [43].

Compound **5**: (+)-(6*S*,8*S*,7′S*) discorhabdin K was isolated from *Latrunculia kaakaariki,* collection and isolation details of which are reported in the SI.

Compound **6**: (+)-(6*S*,8*S*,7′S*) discorhabdin K methyl ester was isolated from *Latrunculia kaakaariki*, collection and isolation details of which are reported in the SI.

Compound **7**: (+)-(6*R*,8*S*) thiomethyldiscorhabdin G*/I was isolated from *Latrunculia purpurea*, collection and isolation details of which are reported in the SI.

Compound **8**: (+)-(6*S*) *N*-13-demethyldiscorhabdin U was semi-synthesized from (+)-(6*S*,8*S*) discorhabdin B details of which are reported below.

Compound **9**: (+)-(6*S*) sulphonyl-7,8-dehydrodiscorhabdin E was isolated from *Latrunculia brevis*, collection and isolation details of which are reported in the SI.

Compound **10**: (−)-(6*S*,6′*S*) discorhabdin W from *Latrunculia* sp., collection and isolation details of which have previously been reported [70].

Compound **11**: (+)-(2*S*,6*R*,8*S*) discorhabdin D was isolated from *Latrunculia brevis*, collection and isolation details of which have previously been reported [43].

Compound **12**: (−)-(1*R*,2*S*,6*R*,8*S*) discorhabdin L was isolated from *Latrunculia brevis*, collection and isolation details of which have previously been reported [43].

Compound **13**: (−)-(1*R*,2*S*,6*R*,8*S*) discorhabdin N was isolated from *Latrunculia brevis*, collection and isolation details of which have previously been reported [43].

Compound **14**: (−)-(1*R*,2*R*,6*R*,8*S,*7′S*) discorhabdin H was isolated from *Latrunculia brevis*, collection and isolation details of which have previously been reported [43].

Compound **15**: discorhabdin C was isolated from *Latrunculia* sp., collection and isolation details of which have previously been reported [45].

Compound **16**: 3-dihydrodiscorhabdin C was isolated from *Latrunculia brevis*, collection and isolation details of which have previously been reported [43].

Compound **17**: discorhabdin P was semi-synthesized from discorhabdin C details of which have previously been reported [45].

Compound **18**: 14-methyldiscorhabdin C was semi-synthesized from discorhabdin C details of which are reported below.

Compound **19**: 14-bromodiscorhabdin C was isolated from *Tsitsikamma pedunculata* collection and isolation details of which are reported in the SI.

Compound **20**: 7,8-dehydrodiscorhabdin C was isolated from *Latrunculia brevis* collection and isolation details of which are reported in the SI.

Compound **21**: didebromodiscorhabdin C was isolated from *Latrunculia brevis* collection and isolation details of which are reported in the SI.

Compound **22**: (+)-(6*S*)-discorhabdin E was isolated from *Latrunculia brevis* collection and isolation details of which are reported in the SI.

Compound **23**: (+)-(6*S*)-*N*-13-methyldiscorhabdin E was semi-synthesized from discorhabdin E details of which are reported below.

Compound **24**: discorhabdin C phenol was semi-synthesized from discorhabdin C details of which are reported below.

### 4.4. Preparation of Semi-Synthetic Derivatives

Compound **8** was prepared using a previously established semi-synthetic route [45]. Discorhabdin B trifluoroacetate salt (**2**) (3.0 mg, 7.2 μmol) was dissolved in dry acetone (3 mL) to which CH_3_I (1 μL, 16.2 μmol) and K_2_CO_3_ (8 mg) were added. The reaction mixture was kept under N_2_ at reflux at 80 °C for 2 h. After the end of the reaction, which was indicated by LC-MS analysis, the products were loaded onto a pre-column cartridge filled with dental cotton, dried overnight, and purified by C_18_ chromatography with a Luna C_18_ (10 µm, 150 × 21.2 mm) column (water acidified with 0.1% TFA/methanol from 98:2 to 6:4 over 70 min) yielding *N*-13-demethyldiscorhabdin U **8** (1.4 mg, 46% yield).

Compound **18**: Discorhabdin C trifluoroacetate salt (**15**) (5.9 mg, 12.8 μmol) was dissolved in DMSO (200 μL) to which FeCl_2_ (0.36 mg, 2.8 μmol) and H_2_O_2_ (2 μL, 85.2 μmol) were added. The reaction mixture was kept under N_2_ at reflux at 25 °C for five hours. After the end of the reaction which was indicated by LC-MS analysis, the products were loaded onto a pre-column cartridge filled with dental cotton, dried overnight, and purified with C_8_ chromatography with a Phenomenex Kinetex C_8_ (5 µm, 150 × 21.2 mm) column (water acidified with 0.1% TFA/methanol from 98:2 to 6:4 over 50 min) yielding 14-methyldiscorhabdin C **18** (1.5 mg, 25% yield).

Compound **23**: Discorhabdin E trifluoroacetate salt (**22**) (2.4 mg, 8.9 μmol) was dissolved in dry acetone (2 mL), to which CH_3_I (8.3 μL, 133.0 μmol) and K_2_CO_3_ (10.4 mg) were added. The reaction mixture was kept under N_2_ at reflux at 80 °C for three hours. After the end of the reaction which was indicated by LC-MS analysis, the products were loaded onto a pre-column cartridge filled with dental cotton, dried overnight and purified using C_18_ chromatography with a Luna C_18_ (10 µm, 150 × 21.2 mm) column (water acidified with 0.1% TFA/methanol from 98:2 to 75:25 over 50 min) yielding 13-methyl-discorhabdin E **23** (0.3 mg, 12.5% yield).

Compound **24** was prepared using a previously established semi-synthetic route [72]. Discorhabdin C trifluoroacetate salt (**15**) (4.5 mg, 9.7 μmol) was dissolved in concentrated H_2_SO_4_ (3 mL) and left at room temperature for 5 min. The reaction mixture was neutralized by the addition of solid NaHCO_3_. The products were loaded onto a pre-column cartridge filled with dental cotton and purified by C_4_ chromatography with a Luna C_4_ (10 µm, 250 × 10 mm) column (water acidified with 0.1% TFA/methanol from 98:2 to 7:3 over 70 min) yielding discorhabdin C phenol **24** (0.9 mg, 25% yield).

### 4.5. Compound Characterization

Compound **5**: (+)-(6*S*,8*S*,7′S*) discorhabdin K TFA salt, brown amorphous powder; [α]_D_ = +177.8, [α]_546_ = −50.8, [α]_633_ = +311.1 (*c* 0.06, MeOH); NMR spectroscopic and chiroptical data in agreement with those previously reported [26]; (+)-HRESIMS *m*/*z* [M + H − CF_3_COO^−^]^+^ 535.1219 (calculated for C_25_H_23_N_6_O_4_S_2_^+^, 535.1217).

Compound **6**: (+)-(6*S*,8*S*,7′S*) discorhabdin K methyl ester TFA salt, brown amorphous powder; [α]_D_ = +407.2, [α]_546_ = −142.0, [α]_633_ = +688.0 (*c* 0.1, MeOH); UV (MeOH) λ_max_ (log ε) 242 (3.76), 279 (3.97), 332 (3.87), 409 (3.65) nm; ECD (MeOH) λ (∆ε) 212 (−13.2), 240 (−4.4), 262 (−15.8), 306 (−7.6), 332 (0), 363 (+14.2), 433 (0) nm; IR (film) 3006, 1677, 1634, 1529, 1436, 1418, 1364, 1331, 1308, 1203, 1132, 1030, 1004, 832, 801, 728 cm^−1^; ^1^ H NMR (DMSO-*d*_6_, 600 MHz) and ^13^C NMR (DMSO-*d*_6_, 151 MHz) data, see Table 1; (+)-HRESIMS *m*/*z* [M + H − CF_3_COO^−^]^+^ 549.1374 (calculated for C_26_H_25_N_6_O_4_S_2_^+^, 549.1374).

Compound **7**: (+)-(6*R*,8*S*) thiomethyldiscorhabdin G*/I TFA salt, green-brown amorphous powder; [α]_D_ = +53.6, [α]_546_ = −31.6, [α]_633_ = +110.0 (*c* 0.1, MeOH); NMR spectroscopic and chiroptical data in agreement with those previously reported [73]; (+)-HRESIMS *m*/*z* [M + H − CF_3_COO^−^]^+^ 382.0681 (calculated for C_19_H_16_N_3_O_2_S_2_^+^, 382.0679).

Compound **8**: (+)-(6*S*) *N*-13-demethyldiscorhabdin U TFA salt, green amorphous powder; [α]_D_ = +177.8, [α]_546_ = −50.8, [α]_633_ = +311.1 (*c* 0.06, MeOH); NMR spectroscopic and chiroptical data in agreement with those previously reported [45]; (+)-HRESIMS *m*/*z* [M + H − CF_3_COO^−^]^+^ 428.0066 (calculated for C_19_H_15_^79^BrN_3_O_2_S^+^, 428.0063).

Compound **9**: (+)-(6*S*) 5-sulphonyl-7,8-dehydro-discorhabdin E TFA salt, green amorphous powder; [α]_D_ +29.9, [α]_546_ = +41.8, [α]_633_ = +23.9 (*c* 0.066, MeOH); UV (MeOH) λ_max_ (log ε) 246 (3.45), 307 (3.25), 433 (3.13) nm; ECD (MeOH) λ (∆ε) 208 (+0.6), 227 (−3.7), 243 (0), 258 (+4.2), 274 (0), 372 (+0.4) nm; IR (film) 1683, 1442, 1202, 1143, 846, 803, 725 cm^−1^; ^1^H NMR (DMSO-*d*_6_, 600 MHz) and ^13^C NMR (DMSO- *d*_6_, 151 MHz) data, see Table 1; (+)-HRESIMS *m*/*z* [M + H − CF_3_COO^−^]^+^ 461.9751 (calculated for C_18_H_13_^79^BrN_3_O_5_S^+^, 461.9754), 463.9730 (calculated for C_18_H_13_^81^BrN_3_O_5_S^+^, 463.9734).

Compound **18**: 14-methyl-discorhabdin C TFA salt, brown amorphous powder; IR (film) 3119, 1674, 1589, 1551, 1518, 1324, 1202, 1137, 1024, 833, 799, 724, 700 cm^−1^; ^1^H NMR (DMSO-*d*_6_, 600 MHz) 13.05 (s, H-13), 10.17 (s, H-9), 7.71 (s, H-1/H-5), 3.67 (m, H-17), 3.61 (m, H-8), 2.73, (t, *J* = 7.4 Hz, H-16), 2.25 (s, H-22), 2.00 (t, *J* = 5.6 Hz, H-7), ^13^C NMR (DMSO- *d*_6_, 151 MHz) 171.5 (C, C-3), 163.7 (C, C-11), 153.0 (C, C-19), 152.3 (C, C-10), 151.2 (CH, C-1, C-5), 139.3 (C, C-14), 124.5 (C, C-21), 122.6 (C, C-2, C-4), 121.1 (C, C-12), 118.1 (C, C-15), 91.4 (C, C-20), 44.7 (C, C-6), 43.7 (CH_2_, C-17), 38.3 (CH_2_, C-8), 33.6 (CH_2_, C-7), 17.3 (CH_2_, C-16), 11.1 (CH_3_, C-22); (+)-HRESIMS *m*/*z* [M + H − CF_3_COO^−^]^+^ 475.9600 (calculated for C_19_H_16_^79^Br_2_N_3_O_2_^+^, 475.9604).

Compound **19**: 14-bromodiscorhabdin C TFA salt, dark purple oil; NMR spectroscopic data in agreement with those previously reported [54]; HRESIMS *m*/*z* [M + H − CF_3_COO^−^]^+^ 539.85562 (calculated for C_18_H_13_Br_3_N_3_O_2_^+^, 539.85524).

Compound **20**: 7,8-dehydro-discorhabdin C TFA salt, green amorphous powder; UV (MeOH) λ_max_ 220, 262, 310, 440, 595 nm; IR (film) 3423, 1679, 1211, 1136, 1024, 1006, 843, 802, 727 cm^−1^; ^1^H NMR (DMSO-*d*_6_, 600 MHz) and ^13^C NMR (DMSO-*d*_6_, 151 MHz) data, see Table 1; (+)-HRESIMS *m*/*z* [M + H − CF_3_COO^−^]^+^ 459.93296 (calculated for C_18_H_12_^79^Br_2_N_3_O_2_^+^, 459.9291), 461.9276 (calculated for C_18_H_12_^79^Br^81^BrN_3_O_2_^+^, 461.9271), 463.9260 (calculated for C_18_H_12_^81^Br_2_N_3_O_2_^+^, 463.9250).

Compound **21**: didebromodiscorhabdin C TFA salt, light pink amorphous powder; NMR spectroscopic data in agreement with those previously reported [74]; (+)-HRESIMS *m*/*z* [M + H − CF_3_COO^−^]^+^ 306.1236 (calculated for C_18_H_16_N_3_O_2_^+^, 306.1237).

Compound **22**: (+)-(6*S*) discorhabdin E TFA salt, purple amorphous powder; [α]_D_ = +17.2, [α]_546_ = +6.8, [α]_633_ = +28.0 (*c* 0.1, MeOH); ECD (MeOH) λ (∆ε) 207 (−5.9), 218 (−0.6), 230 (−1.4), 240 (0), 248 (+2.1), 261 (0), 274 (−0.7), 337 (0), 368 (+0.6), 445 (0), nm; NMR spectroscopic and chiroptical data in agreement with those previously reported [72]; (+)-HRESIMS *m*/*z* [M + H − CF_3_COO^−^]^+^ 384.0341 (calculated for C_18_H_15_^79^BrN_3_O_2_^+^, 384.0343).

Compound **23**: (+)-(6*S*) *N*-13-methyldiscorhabdin E TFA salt, pink amorphous powder; [α]_D_ = +177.8, [α]_546_ = −50.8, [α]_633_ = +311.1 (*c* 0.06, MeOH); IR (film) 3255, 1681, 1447, 1207, 1135, 839, 804, 723; ^1^ H NMR (DMSO-*d*_6_, 600 MHz) 10.08 (br s, H-9), 7.70 (d, *J* = 2.8, H-1), 7.39 (s, H-14), 7.12 (dd, *J* = 2.8, 9.8 Hz, H-5), 6.47 (d, *J* = 9.8 Hz, H-4), 3.92 (s, H-22), 3.66 (m, H-17), 3.62 (m, H-8), 2.78 (m, H-16), 1.90 (m, H-7), 1.96 (m, H-7); ^13^C NMR (DMSO-*d*_6_, 151 MHz) 178.1 (C, C-3), 154.2 (C, C-19), 152.0 (C, C-10), 151.8 (CH, C-5), 151.6 (CH, C-1), 132.0 (C, C-14), 129.6 (C, C-4), 125.5 (C, C-2), 124.8 (C, C-12), 123.1 (C, C-21), 119.4 (C, C-15), 92.6 (C, C-20), 44.1 (CH_2_, C-17), 43.0 (C, C-6), 39.8 (CH_2_, C-8), 36.5 (CH_3_, C-22), 34.8 (CH_2_, C-7), 18.9 (CH_2_, C-16); (+)-HRESIMS *m*/*z* [M + H − CF_3_COO^−^]^+^ 398.0499 (calculated for C_19_H_17_^79^BrN_3_O_2_^+^, 398.0499).

Compound **24**: discorhabdin C phenol TFA salt, purple amorphous powder; NMR spectroscopic data in agreement with those previously reported [72]; (+)-HRESIMS *m*/*z* [M + H − CF_3_COO^−^]^+^ 461.9450 (calculated for C_18_H_14_^79^Br_2_N_3_O_2_^+^, 461.9448).

### 4.6. Computational Methods

Three-dimensional structures of the molecules were drawn and subjected to conformational analysis in ComputeVOA (BioTools Inc., Jupiter, FL, USA) using MMFF94 as a force field and the GMMX methodology on a Windows operating system machine. Geometrical optimization and energy calculation of conformers occurring in an energy window (ΔΕ) of 0–3 Kcal/mol were done by implementation of B3LYP/DGDZVP using the COSMO solvation algorithm in Gaussian 16 software. The optimized structures (Tables S1 and S2) were used to calculate the thermochemical parameters estimated at 298 K and 1 atm. Calculations taking into account the solvent (MeOH) were carried out starting from DFT-optimized structures. Optimized conformers were then subjected to TDDFT calculations in MeOH on Gaussian 16 using B3LYP/DGDZVP to obtain the ECD spectra. All quantum mechanical calculations were carried out using the Gaussian 16 software on a Linux operating system in the Biowulf cluster. Obtained ECD spectra with half-band of 0.25 eV and UV shift of 0 nm were Boltzmann-averaged and scaled using the SpecDis program spectra and compared with experimental spectra obtained in MeOH.

### 4.7. Cell lines Used

Three VN-MCC cell lines (MCC13 and MCC26 [75], and UISO [76]), along with three VP-MCC cell lines (MKL-1 [77], MKL-2 [78], and Waga [79]) and HaCaT immortalized keratinocytes [80] (ThermoFisher, Waltham, MA, USA) were used. Cell identity was confirmed, and cells were maintained and utilized as previously described [22].

### 4.8. Cell Viability and Analysis of Cell Death Mechanisms

Three methods were employed for assessment of MCC and control cell survival/viability after discorhabdin treatment, two metabolic assays and a cell permeability assay. For all three methods, cells were plated in 384-well plates at 2500 cells/well and treated with various concentrations of discorhabdins for various time periods. CellTiterGlo^TM^ (Promega, Madison, WI, USA) measures cellular ATP content and was used to estimate potency of discorhabdins in cell viability assays as previously described [22]. Continuous monitoring of metabolic activity was accomplished using a cellular reducing potential luminescence assay [81], the RealTime-Glo^TM^ MT Cell Viability Assay (Promega, Madison, WI, USA) according to manufacturer’s instructions. Loss of cell plasma membrane integrity was assessed using a cell-impermeant molecule that becomes fluorescent upon binding DNA [82] in parallel with apoptosis detection [83] per manufacturer’s instructions (RealTimeGlo^TM^ Annexin V Apoptosis and Necrosis Assay-Promega). Two additional assays of apoptotic cell death were also used. Caspase activation in response to treatment of MCC cells by discorhabdins or bortezomib (as a positive control) was measured using the CaspaseGlo^®^ 3/7 Assay (Promega, Madison, WI, USA) according to the manufacturer’s instructions at multiple time points. Inhibition of apoptotic cell death was investigated using pre-treatment of cells with for 1 h with the pan-caspase inhibitor Z-VAD-FMK (Enzo Life Sciences, Farmingdale, NY, USA) followed by treatment with discorhabdins or bortezomib and estimation of cell viability as outlined above.

Mitochondrial membrane potential (MMP) was assessed using the JC-10 ratiometric dye (Sigma, St. Louis, MO, USA). UISO or MKL-1 cells were treated in black-walled, clear-bottom 384-well plates (Corning Life Sciences, Durham, NC, USA) with discorhabdins or doxorubicin (Sigma, St. Louis, MO, USA) as a positive control for up to 24 h with JC-10 for the last 1 h of incubation. Red/green fluorescence signals were measured, the red/green ratio for each well was calculated and normalized to untreated (DMSO) control wells for the same cell line and incubation time to determine relative MMP.

For the detection of reactive oxygen species (ROS), cells in black-walled clear-bottom 384-well plates were preloaded with DCFDA per manufacturer’s protocol (ThermoFisher, Waltham, MA, USA), washed, then treated with discorhabdins or TBHP (Sigma, St. Louis, MO, USA) as a positive control. Fluorescence intensity (485 nm excitation, 535 nm emission) was measured at multiple time intervals up to 4 h and normalized to untreated (DMSO) controls for each cell line at the same time point.

Calcium mobilization was assessed with Fluo-4 (Fluo-4 NW Calcium Assay Kit—ThermoFisher) per manufacturer’s protocols. Cells in black-wall, clear-bottom 384-well plates were pre-loaded with Fluo-4 followed by treatment with 10 µM discorhabdins or 20 µM thapsigargin (Sigma, St. Louis, MO, USA) as a positive control to monitor fluorescence. Fluorescence signals were normalized to DMSO controls at the same time/cell line.

### 4.9. Data Analysis

In order to compare responses among all of the discorhabdins and cell lines (98 combinations), dose–response curves were generated using identical discorhabdin dilution series. GraphPad Prism 8 (San Diego, CA, USA) software was utilized to graph dose–response data and to calculate IC_50_ values and area under the dose–response curves (AUC) along with 95% confidence interval estimates using four-parameter logistic nonlinear regression analysis. For further analysis and comparisons, AUC for each cell line/discorhabdin pair was visualized in a heatmap generated in R version 1.3.1073 (R Core Team (2013). R: A language and environment for statistical computing. R Foundation for Statistical Computing, Vienna, Austria, http://www.R-project.org/ (accessed on 12 January 2023).

## Figures and Tables

**Table 1:** ^1^H and ^13^C NMR (600/151 MHz, DMSO-d_6_) spectroscopic data of compounds **6**, **9**, and **20**.

No.	Compound 6		Compound 9		Compound 20	
	*δ*_H_ Mult. (*J* Hz)	*δ*_C_ Mult.	*δ*_H_ Mult. (*J* Hz)	*δ*_C_ Mult.	*δ*_H_ Mult. (*J* Hz)	*δ*_C_ Mult.
1	-	160.1, C	7.77 s	150.1, CH	7.86 s	150.0, CH
2	5.75 s	125.3, CH	-	122.0, C	-	121.1, C
3	-	179.3, C	-	178.6, C	-	170.9, C
4	6.14 s	118.5, CH	6.61 s	124.2, CH	-	121.1, C
5	-	167.8, C	-	163.9, C	7.86 s	150.0, CH
6	-	50.7, C	-	46.3, C	-	47.1 *, C
7	2.59 dd (3.6,12.0) 2.93 m	42.1, CH_2_	4.51 d (7.4)	113.5, CH	4.71 d (7.4)	109.8, CH
8	5.69 d (3.6)	59.2, CH	6.33 dd (5.0,7.4)	124.6, CH	6.53 dd (5.0,7.4)	125.6, CH
9	10.85 s	-	10.38 d (5.0)	-	10.71 d (5.0)	-
10	-	151.5, C	-	143.8, C	-	145.7 *, C
11	-	164.9, C	-	166.7, C	-	167.0, C
12	-	123.7, C	-	123.6, C	-	123.7, C
13	13.36 br d (2.0)	-	13.22 br d (2.2)	-	13.33 br d (2.0)	-
14	7.42 d (2.0)	127.6, CH	7.37 d (2.8)	126.9, CH	7.41 d (2.8)	127.2, CH
15	-	120.5, C	-	119.3, C	-	119.6, C
16	2.86 m	17.7, CH_2_	2.82, m	18.0, CH_2_	2.87, t (8.0)	17.8, CH_2_
17	3.87 m 3.95 m	44.9, CH_2_	3.61 m 3.75 m	44.4, CH_2_	3.78 t (8.0)	44.5, CH_2_
19	-	153.6, C	-	158.0, C	-	157.7, C
20	-	97.6, C	-	97.2, C	-	95.4 *, C
21	-	122.8, C	-	122.7, C	-	122.2, C
1′	-	123.1, C	-	-	-	-
3′	8.01 s	141.2, CH	-	-	-	-
5′	-	132.6, C	-	-	-	-
6′	3.19 dd (9.6,15.2) 3.26 dd (6.0,15.2)	24.2, CH_2_	-	-	-	-
7′	4.20 m	50.9, CH	-	-	-	-
8′	-	168.7, C	-	-	-	-
9′	3.69 s	32.3, CH_3_	-	-	-	-
10′	3.61 s	53.0, CH_3_	-	-	-	-
11′	8.74 br s	-	-	-	-	-

[*] Chemical shift determined from the ^1^H–^13^C HMBC spectrum.

## Data Availability

All data are contained in this article and associated Supplementary Materials.

## Supplementary Materials

The following supporting information can be downloaded at: https://www.mdpi.com/article/10.3390/md21090474/s1, collection, extraction, and isolation procedures, HRMS, 1D and 2D NMR, IR spectra of compounds **6**, **9**, **18**, **20**, **23**, population of Boltzmann averaged conformers of compounds **9** and **22**, calculated and experimental ECD spectra for compound **6** and **22**, physicochemical properties of discorhabdins and effects of discorhabdins on MCC cells.
