# Supplementary material for: An Investigation of Structure–Activity Relationships and Cell Death Mechanisms of the Marine Alkaloids Discorhabdins in Merkel Cell Carcinoma Cells
Source: Mar Drugs. 2023 Aug 29;21(9):474. doi: 10.3390/md21090474 (PMC10532587; doi:10.3390/md21090474)

Supplementary material for

An Investigation of Structure-Activity Relationships and Cell Death Mechanisms of the Marine Alkaloids Discorhabdins in Merkel Cell Carcinoma Cells

Maria Orfanoudaki,[1,†] Emily A. Smith,[1,2,†] Natasha Hill,[3] Khalid A. Garman,[3] Isaac Brownell,[3] Brent R. Copp,[4] Tanja Grkovic,*[1,5] Curtis J. Henrich*[1,2]

[1]Molecular Targets Program, Center for Cancer Research, National Cancer Institute, Frederick, Maryland 21702

[2]Basic Science Program, Frederick National Laboratory for Cancer Research, Frederick, Maryland 21702

[3]Dermatology Branch, National Institute of Arthritis and Musculoskeletal and Skin Diseases, Bethesda, Maryland 20891

[4]School of Chemical Sciences, University of Auckland, Auckland, New Zealand

[5]Natural Products Branch, Division of Cancer Treatment and Diagnosis, National Cancer Institute, Frederick, Maryland 21702

[†]These authors contributed equally to this work.

*correspondence:

tanja.grkovic@nih.gov, henrichcj@mail.nih.gov

Table of Contents

Collection, extraction and isolation 5

Table S1. Population of Boltzmann averaged conformers of compound **9**. 10

Table S2. Population of Boltzmann averaged conformers of compound **22.** 10

Figure S1. Calculated ECD spectra (blue line) for compound **22** compared to the experimental spectrum observed for compound **22** (red line). 11

Figure S2: Physicochemical properties of discorhabdins 11

Figure S3. Experimental ECD spectra of compound **5** (red line) and compound **6** (blue line). 12

Figure S4. Product-ion spectrum of compound **9** *m/z* $[M-H]^-$ 459.9607 at collision energy setting of 10 eV under negative ion ESI mode. 12

Figure S5. ^1H NMR spectrum (600.0 MHz, DMSO-d_6) for compound **6**. The spectrum was acquired over 4 scans. 13

Figure S6. ^{13}C NMR spectrum (150.9 MHz, DMSO-d_6) for compound **6**. The spectrum was acquired over 13000 scans. 14

Figure S7. ^1H – ^1H COSY NMR spectrum (600.0 MHz, DMSO-d_6) for compound **6**. The spectrum was acquired over 32 scans at 256 t1 increments. 15

Figure S8. Multiplicity edited ^1H – ^{13}C HSQC NMR spectrum (600.0 MHz, DMSO-d_6) for compound **6**. The spectrum was acquired over 32 scans at 256 t1 increments. 16

Figure S9. ^1H – ^{13}C HMBC NMR spectrum (600.0 MHz, DMSO-d_6) for compound **6**. The spectrum was acquired over 520 scans at 256 t1 increments. 17

Figure S10. HRESIMS spectrum for compound **6**. 18

Figure S11. IR spectrum for compound **6**. 19

Figure S12. ^1H NMR spectrum (600.0 MHz, DMSO-d_6) for compound **9**. The spectrum was acquired over 4 scans. 20

Figure S13. ^{13}C NMR spectrum (150.9 MHz, DMSO-d_6) for compound **9**. The spectrum was acquired over 96000 scans. 21

Figure S14. ^1H – ^1H COSY NMR spectrum (600.0 MHz, DMSO-d_6) for compound **9**. The spectrum was acquired over 32 scans at 256 t1 increments. 22

Figure S15. Multiplicity edited ^1H – ^{13}C HSQC NMR spectrum (600.0 MHz, DMSO-d_6) for compound **9**. The spectrum was acquired over 64 scans at 256 t1 increments. 23

Figure S16. ^1H – ^{13}C HMBC NMR spectrum (600.0 MHz, DMSO-d_6) for compound **9**. The spectrum was acquired over 1400 scans at 256 t1 increments. 24

Figure S17. HRESIMS spectrum for compound **9**. 25

Figure S18. IR spectrum for compound **9**. 26

Figure S19. ^{1}H NMR spectrum (600.0 MHz, DMSO-d_6) for compound **20**. The spectrum was acquired over 4 scans. 27

Figure S20. ^{13}C NMR spectrum (150.9 MHz, DMSO-d_6) for compound **20**. The spectrum was acquired over 27000 scans. 28

Figure S21. ^{1}H – ^{1}H COSY NMR spectrum (600.0 MHz, DMSO-d_6) compound **20**. The spectrum was acquired over 64 scans at 256 t1 increments. 29

Figure S22. Multiplicity edited ^{1}H – ^{13}C HSQC NMR spectrum (600.0 MHz, DMSO-d_6) for compound **20**. The spectrum was acquired over 32 scans at 256 t1 increments. 30

Figure S23. ^{1}H – ^{13}C HMBC NMR spectrum (600.0 MHz, DMSO-d_6) for compound **20**. The spectrum was acquired over 1400 scans at 256 t1 increments. 31

Figure S24. HRESIMS spectrum for compound **20**. 32

Figure S25. IR spectrum for compound **20**. 33

Figure S26. ^{1}H NMR spectrum (600.0 MHz, DMSO-d_6) for compound **18**. The spectrum was acquired over 4 scans. 34

Figure S27. ^{13}C NMR spectrum (150.9 MHz, DMSO-d_6) for compound **18**. The spectrum was acquired over 10000 scans. 35

Figure S28. ^{1}H – ^{1}H COSY NMR spectrum (600.0 MHz, DMSO-d_6) for compound **18**. The spectrum was acquired over 8 scans at 256 t1 increments. 36

Figure S29. Multiplicity edited ^{1}H – ^{13}C HSQC NMR spectrum (600.0 MHz, DMSO-d_6) for compound **18**. The spectrum was acquired over 16 scans at 256 t1 increments. 37

Figure S30. ^{1}H – ^{13}C HMBC NMR spectrum (600.0 MHz, DMSO-d_6) for compound **18**. The spectrum was acquired over 600 scans at 256 t1 increments. 38

Figure S31. HRESIMS spectrum for compound **18**. 39

Figure S32. IR spectrum for compound **18**. 40

Figure S33. ^{1}H NMR spectrum (600.0 MHz, DMSO-d_6) for compound **23**. The spectrum was acquired over 4 scans. 41

Figure S34. ^{1}H – ^{1}H COSY NMR spectrum (600.0 MHz, DMSO-d_6) for compound **23**. The spectrum was acquired over 8 scans at 256 t1 increments. 42

Figure S35. Multiplicity edited ^{1}H – ^{13}C HSQC NMR spectrum (600.0 MHz, DMSO-d_6) for compound **23**. The spectrum was acquired over 16 scans at 256 t1 increments. 43

Figure S36. ^{1}H – ^{13}C HMBC NMR spectrum (600.0 MHz, DMSO-d_6) for compound **23**. The spectrum was acquired over 4000 scans at 128 t1 increments. 44

Figure S37. HRESIMS spectrum for compound **23**. 45

Figure S38. IR spectrum for compound **23**. 46

Figure S39: Activity of discorhabdins against MCC cell lines. 47

Table S3: Activity of discorhabdins against MCC cell lines (area under the curve). 54

Figure S40: Activity of discorhabdins against MCC cell lines – comparison of IC_{50} and AUC 56

Figure S41: Discorhabdin selectivity 57

Figure S42: Caspase activation in response to discorhabdin treatment. 58

Figure S43: Apoptotic/necrotic signals in MCC cells in response to discorhabdins 59

Figure S44: Effect of caspase inhibitor on MCC cell response to discorhabdins 60

Figure S45: Effects of discorhabdins and bortezomib on MCC cell reductive potential 61

Figure S46: Effects of discorhabdins, doxorubicin (DOX) and bortezomib on MCC cell mitochondrial potential 62

Figure S47: Discorhabdins do not induce ROS generation in MCC cells 63

Figure S48: Discorhabdins do not increase intracellular calcium in MCC cells 64

Collection, extraction and isolation

The sponge *Tsitsikamma pedunculata* was collected by SCUBA at a depth of 40 m in the Republic of South Africa in March 2000 under contract through the Coral Reef Research Foundation for the National Cancer Institute. The specimen was taxonomically identified by Lori Bell of the Coral Reef Research Foundation, and a voucher specimen (0CDN7414) was deposited at the Smithsonian Institution. The sponge (wet weight: 47.3 g) was extracted in water, followed by a MeOH/DCM overnight soak according to the National Cancer Institute's standard marine extraction procedure detailed in McCloud [1] to give the organic solvent extract C20693 (2.1 g). A portion of the organic extract C20693 (250 mg) was pre-fractionated on a flash C_8 column (2 g) eluting sequentially with: water/methanol (95:5) to yield 34.1 mg of fraction 1, water/methanol (80:20) to yield 2.1 mg of fraction 2, water/methanol (60:40) to yield 4.4 mg of fraction 3, water/methanol (40:60) to yield 16.6 mg of fraction 4, water/methanol (20:80) to yield 17.2 mg of fraction 5, methanol 100% to yield 15.3 mg of fraction 6, and methanol acidified with 0.1% TFA to yield 32.9 mg of fraction 7. Fractions 3, 4, and 5 eluting with 40, 60, and 80% methanol respectively, were further purified on C_8 preparative HPLC using a Phenomenex Kinetex C_8 [5 µm, 150 × 21.2 mm] column at a flow rate of 10 mL/min eluting with a steep gradient from water/acetonitrile (9:1) to water/acetonitrile (1:1) over 60 minutes. The conditions were isocratic at water/acetonitrile (9:1) for 5 minutes followed by a gradient from water/acetonitrile (9:1) to water/acetonitrile (1:1) over 45 minutes, followed by isocratic water/acetonitrile (1:1) conditions over 10 minutes. Subfraction 24 yielded 14-bromo discorhabdin C (**19**) 17.5 mg (7.0 % crude organic extract weight). For analytical data acquisition, compound **19** was converted to the trifluoroacetate salt using methanol [0.1% TFA].

The sponge *Latrunculia purpurea* Carter, 1881 was collected by SCUBA at a depth of 16 meters in South-eastern Australia in February 1990 under contract through SeaPharm for the National Cancer Institute. The specimen was taxonomically identified by Michelle Kelly, and a voucher specimen (Q66C3468) was deposited at the Smithsonian Institution. The sponge (wet weight 203.8 g) was extracted in water, followed by a MeOH/DCM overnight soak according to the National Cancer Institute's standard marine extraction procedure detailed in McCloud [1] to give the organic solvent extract C3877 (9.5 g). A portion of the organic extract C3877 (253 mg) was pre-fractionated on a flash C_8 column (2 g) eluting sequentially with: water/methanol (95:5) to yield

19.2 mg of fraction 1, water/methanol (80:20) to yield 10.9 mg of fraction 2, water/methanol (60:40) to yield 7.3 mg of fraction 3, water/methanol (40:60) to yield 18.0 mg of fraction 4, water/methanol (20:80) to yield 30.3 mg of fraction 5, methanol 100% to yield 27.0 mg of fraction 6, and methanol/ CH_3CN (1/1) to yield 40.8 mg of fraction 7. Fractions 3, 4, 5 and 6 eluting with 40, 60, 80 and 100% methanol respectively, were further purified on C_{18} preparative HPLC using a Luna C_{18} [10 µm, 150 × 21.2 mm] column at a flow rate of 16 mL/min eluting with a gradient from water acidified with 0.1% TFA /methanol (98:2) to water acidified with 0.1% TFA /methanol (2:8) over 50 minutes. The conditions were isocratic at water acidified with 0.1% TFA /methanol (98:2) for 5 minutes followed by a gradient from water acidified with 0.1% TFA /methanol (98:2) to water acidified with 0.1% TFA /methanol (2:8) over 50 minutes, followed by methanol 100% over 10 minutes. Subfractions 48-50 were further purified with C_4 preparative HPLC using a Luna C_4 [10 µm, 250 × 10 mm] column at a flow rate of 6 mL/min eluting with a gradient from water acidified with 0.1% TFA /methanol (98:2) to water acidified with 0.1% TFA /methanol (75:25) over 70 minutes to give compound (+)-(6*R*,8*S*)-thiomethyl-discorhabdin I (**7**) 0.9 mg (0.4 % crude organic extract weight).

The sponge *Latrunculia brevis* Ridley & Dendy, 1886 was collected by SCUBA at a depth of 12 meters in Northern New Zealand in August 1991 under contract through SeaPharm for the National Cancer Institute. The specimen was taxonomically identified by by Chris Battershill, and voucher specimen (Q66C2025) was deposited at the Smithsonian Institution. The sponge (wet weight 142.8 g) was extracted in water, followed by a MeOH/DCM overnight soak according to the National Cancer Institute's standard marine extraction procedure detailed in McCloud [1] to give the aqueous solvent extract the aqueous solvent extract C5076 (36.8 g) and the organic solvent extract C5077 (5.6 g). A portion of the organic extract C5077 (495.7 mg) was pre-fractionated on a flash C_8 column (2 g) eluting sequentially with: water/methanol (95:5) to yield 83.6 mg of fraction 1, water/methanol (80:20) to yield 25.7 mg of fraction 2, water/methanol (60:40) to yield 16.3 mg of fraction 3, water/methanol (40:60) to yield 48.8 mg of fraction 4, water/methanol (20:80) to yield 41.0 mg of fraction 5, methanol 100% to yield 67.5 mg of fraction 6, and methanol/ CH_3CN (1/1) to yield 179.7 mg of fraction 7. Fractions 2-6 were further purified on C_{18} preparative HPLC using a Luna C_{18} [10 µm, 150 × 21.2 mm] column at a flow rate of 16 mL/min eluting with a gradient from water acidified with 0.1% TFA /methanol (98:2) to water acidified with 0.1% TFA /methanol

(2:8) over 50 minutes. The conditions were isocratic at water acidified with 0.1% TFA /methanol (98:2) for 5 minutes followed by a gradient from water acidified with 0.1% TFA /methanol (98:2) to water acidified with 0.1% TFA /methanol (2:8) over 50 minutes, followed by methanol 100% over 10 minutes. Subfraction 54 was further purified with C_4 preparative HPLC using a Luna C_4 [10 µm, 250 × 10 mm] column at a flow rate of 6 mL/min eluting with a gradient from water acidified with 0.1% TFA /methanol (98:2) to water acidified with 0.1% TFA /methanol (75:25) over 70 minutes, followed by a Sephadex LH-20 (28 × 1 cm) with methanol acidified with 0.1% TFA to give compound (+)-(6*S*)-5-sulphonyl-7,8-dehydro-discorhabdin E (**9**) 0.6 mg (0.12 % crude organic extract weight). Subfractions 55 and 56 were fractionated under the same conditions as subfraction 54 to give compound 7,8-dehydro-discorhabdin C (**20**) 0.3 mg (0.06 % crude organic extract weight). A portion of the aqueous extract C5076 (9.5 g) was pre-fractionated on HP-20SS column eluting sequentially with: water 100% to yield 7.2 g of fraction 1, water/methanol (75:25) to yield 1.3 g of fraction 2, water/methanol (1:1) to yield 563.3 mg of fraction 3, water/methanol (25:75) to yield 291.7 mg of fraction 4, methanol 100% to yield 315.2 mg of fraction 5. Fractions 3 and 4 were further purified on C_{18} preparative HPLC using a Luna C_{18} [10 µm, 150 × 21.2 mm] column at a flow rate of 16 mL/min eluting with a gradient from water acidified with 0.1% TFA /methanol (98:2) to water acidified with 0.1% TFA /methanol (55:45) over 50 minutes. The conditions were isocratic at water acidified with 0.1% TFA /methanol (98:2) for 5 minutes followed by a gradient from water acidified with 0.1% TFA /methanol (98:2) to water acidified with 0.1% TFA /methanol (55:45) over 50 minutes, followed by a gradient from water acidified with 0.1% TFA /methanol (55:45) to water acidified with 0.1% TFA /methanol (20:80) over 10 minutes, followed by methanol 100% over 10 minutes. Subfractions 51-58 were further purified with C_{18} preparative HPLC using a Luna C_{18} [10 µm, 150 × 21.2 mm] column at a flow rate of 16 mL/min eluting with a gradient from water acidified with 0.1% TFA /methanol (98:2) to water acidified with 0.1% TFA /methanol (78:22) over 55 minutes, followed by a gradient from water acidified with 0.1% TFA /methanol (78:22) to water acidified with 0.1% TFA /methanol (20:80) over 5 minutes to give fraction 90 which was subjected to C_{18} preparative HPLC using a Phenomenex Kinetex C_{18} [5 µm, 250 × 10 mm] column at a flow rate of 4.5 mL/min eluting with a gradient from water acidified with 0.1% TFA /methanol (98:2) to water acidified with 0.1% TFA /methanol (7:3) over 30 minutes to give compound didebromodiscorhabdin C (**21**) 1.8 mg (0.019 % crude organic extract weight). Subfractions 62-

68 were further purified with C_{18} preparative HPLC using a Luna C_{18} [10 μm, 150 × 21.2 mm] column at a flow rate of 16 mL/min eluting with a gradient from water acidified with 0.1% TFA /methanol (98:2) to water acidified with 0.1% TFA /methanol (7:3) over 50 minutes, followed by isocratic water acidified with 0.1% TFA /methanol (7:3) conditions over 10 minutes and methanol 100% to yield a fraction which was subjected to Sephadex LH-20 (100 × 1.7 cm) with methanol acidified with 0.1% TFA to give compound (+)-(6*S*)-discorhabdin E (**22**) 1.6 mg (0.017 % crude organic extract weight).

The sponge *Latrunculia kaakaariki* Alvarez, Bergquist & Battershill, 2002 was collected by SCUBA at a depth of 20 meters, respectively in Northern New Zealand in August 1991 under contract through SeaPharm for the National Cancer Institute. The specimens were taxonomically identified by Michelle Kelly, and voucher specimens (Q66C2057) were deposited at the Smithsonian Institution. The sponge (wet weight 193.0 g) was extracted in water, followed by a MeOH/DCM overnight soak according to the National Cancer Institute's standard marine extraction procedure detailed in McCloud [1] to give the aqueous solvent extract C5118 (50.0 g). A portion of the aqueous extract C5118 (40.0 g) was pre-fractionated on HP-20SS column eluting sequentially with: water 100% to yield 12.1 g of fraction 1, water/methanol (75:25) to yield 16.7 g of fraction 2, water/methanol (1:1) to yield 2.3 g of fraction 3, water/methanol (25:75) to yield 1.4 g of fraction 4, methanol 100% to yield 950.8 mg of fraction 5. Fraction 3 was further purified on a HP-C_{18} silica gel column (150 g, 55-105 μm, 125Å) at a flow rate of 85 mL/min eluting with a gradient from water acidified with 0.1% TFA /methanol (98:2) to water acidified with 0.1% TFA /methanol (45:55) over 7 column volumes. The conditions were isocratic at water acidified with 0.1% TFA /methanol (98:2) for 1 column volume followed by a gradient from water acidified with 0.1% TFA /methanol (98:2) to water acidified with 0.1% TFA /methanol (45:55) over 7 column volumes, followed by a gradient water acidified with 0.1% TFA /methanol (45:55) to water acidified with 0.1% TFA /methanol (2:98) over 3 column volumes. Subfractions 51-59 were further purified with C_{18} preparative HPLC using a Luna C_{18} [10 μm, 150 × 21.2 mm] column at a flow rate of 10 mL/min eluting with a gradient from water acidified with 0.1% TFA /methanol (98:2) to water acidified with 0.1% TFA /methanol (6:4) over 70 minutes, followed by a gradient from water acidified with 0.1% TFA /methanol (6:4) to water acidified with 0.1% TFA /methanol (4:6) over 10 minutes to give fraction 62 which was subjected to Sephadex LH-20 (100 × 1.7 cm) with methanol acidified with 0.1% TFA and C_4 preparative HPLC using a Luna C_4 [10 μm, 250 ×

10 mm] column at a flow rate of 6 mL/min eluting with a gradient from water acidified with 0.1% TFA /methanol (98:2) to water acidified with 0.1% TFA /methanol (80:20) over 70 minutes, to give (+)-(6*S*,8*S*,7′*S*)-discorhabdin K (**5**) 40.6 mg (0.10 % crude organic extract weight). Fraction 76 from the last-mentioned C_4 fractionation was further fractionated with C_4 preparative HPLC using a Luna C_4 [10 µm, 250 × 10 mm] column at a flow rate of 6 mL/min eluting with a gradient from water acidified with 0.1% TFA /methanol (98:2) to water acidified with 0.1% TFA /methanol (80:10) over 70 minutes, followed by Sephadex LH-20 (50 × 1.7 cm) with methanol acidified with 0.1% TFA to give (+)-(6*S*,8*S*,7′*S*)-O-methyldiscorhabdin K (**6**) 4.5 mg (0.01 % crude organic extract weight).

Table S1. Population of Boltzmann averaged conformers of compound **9**.

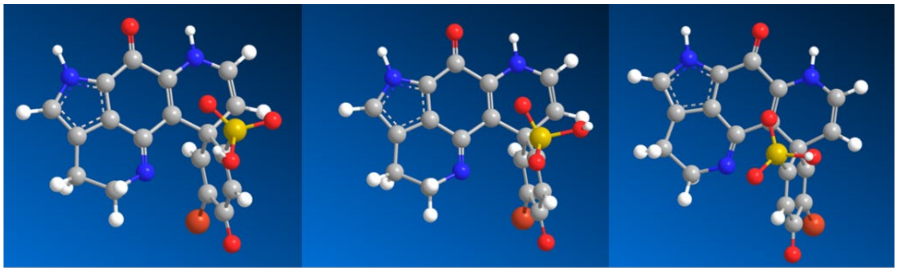

Conformer 1 Conformer 2 Conformer 3

Index	E(au)	E(kcal/mol)	DeltaE(kcal/mol)	Qi(Relat)	Population
Conformer 1	-4205.416687	-2638938.922	0.764306571	0.27503712	20.51%
Conformer 2	-4205.415339	-2638938.077	1.609812872	0.065951352	4.92%
Conformer 3	-4205.417905	-2638939.687	0	1	74.57%

Table S2. Population of Boltzmann averaged conformers of compound **22.**

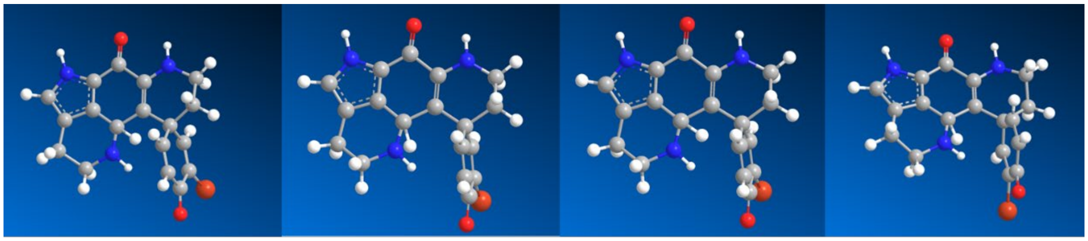

Conformer 1 Conformer 2 Conformer 3 Conformer 4

Index	E(au)	E(kcal/mol)	DeltaE(kcal/mol)	Qi(Relat)	Population
Conformer 1	-3582.8275	-2248258.293	0.361821978	0.542760706	17.71%
Conformer 2	-3582.827892	-2248258.539	0.115901005	0.822220228	26.83%
Conformer 3	-3582.82774	-2248258.444	0.211219698	0.699959908	22.84%
Conformer 4	-3582.828077	-2248258.655	0	1	32.63%

Figure S1. Calculated ECD spectra (blue line) for compound **22** compared to the experimental spectrum observed for compound **22** (red line).

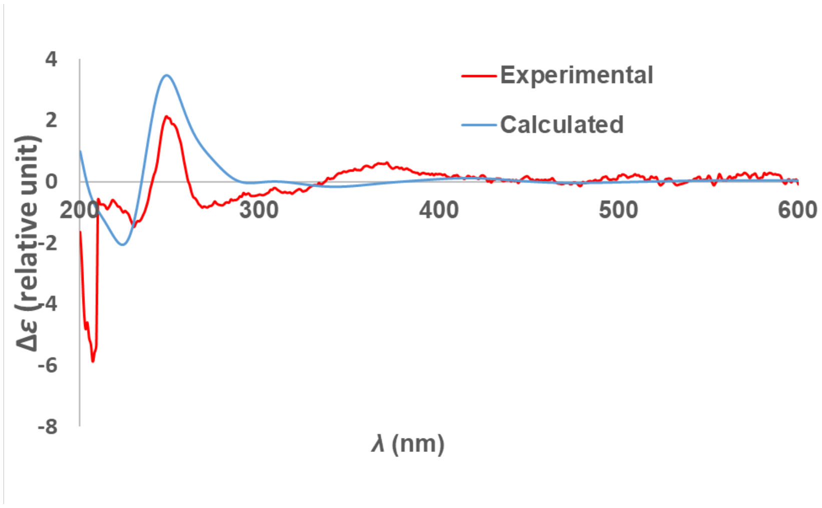

Figure S2: Physicochemical properties of discorhabdins

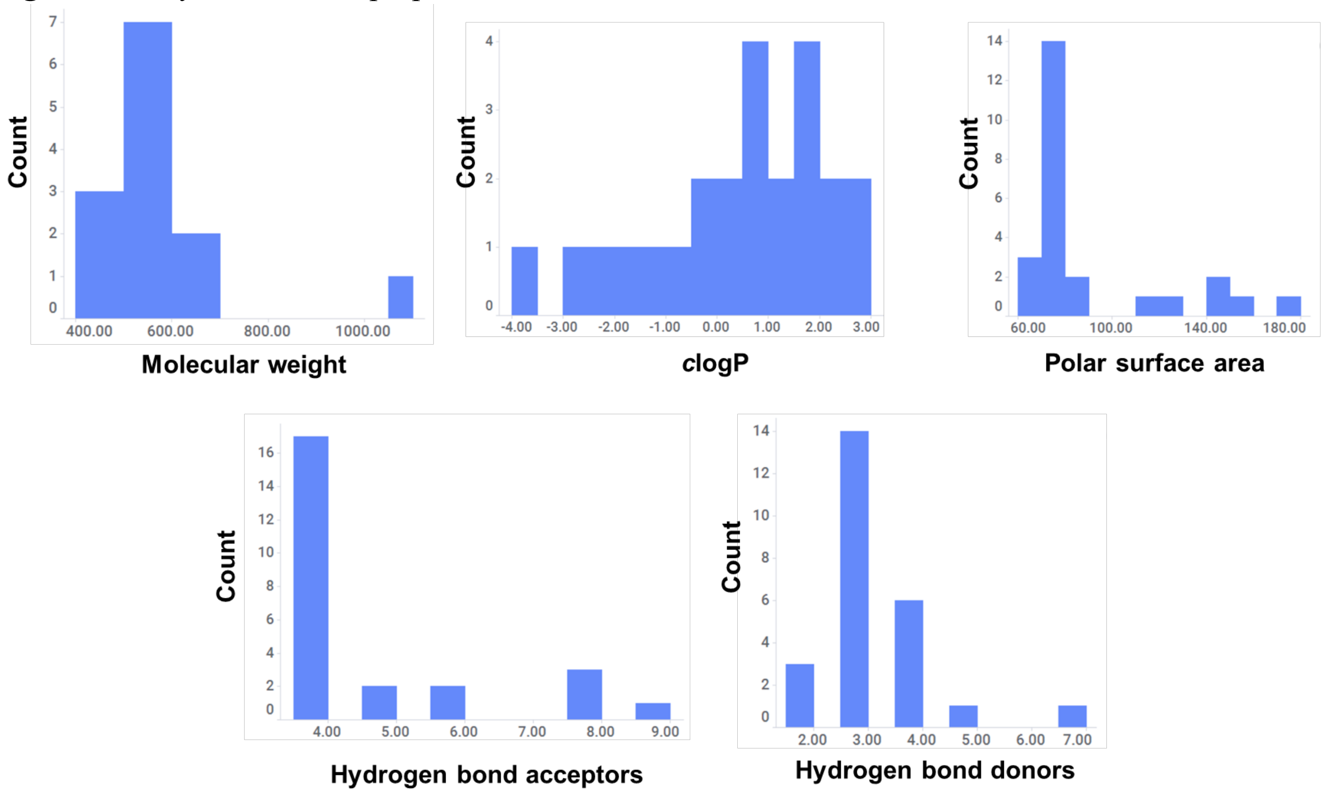

Figure S3. Experimental ECD spectra of compound **5** (red line) and compound **6** (blue line).

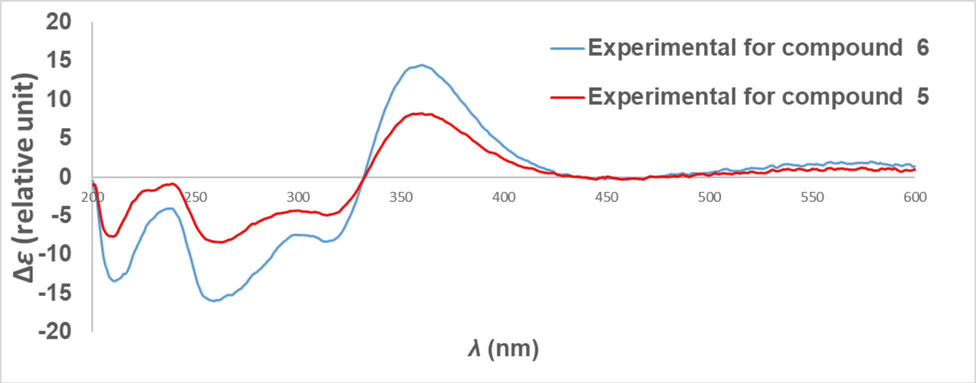

Figure S4. Product-ion spectrum of compound **9** *m/z* [M-H]$^-$ 459.9607 at collision energy setting of 10 eV under negative ion ESI mode.

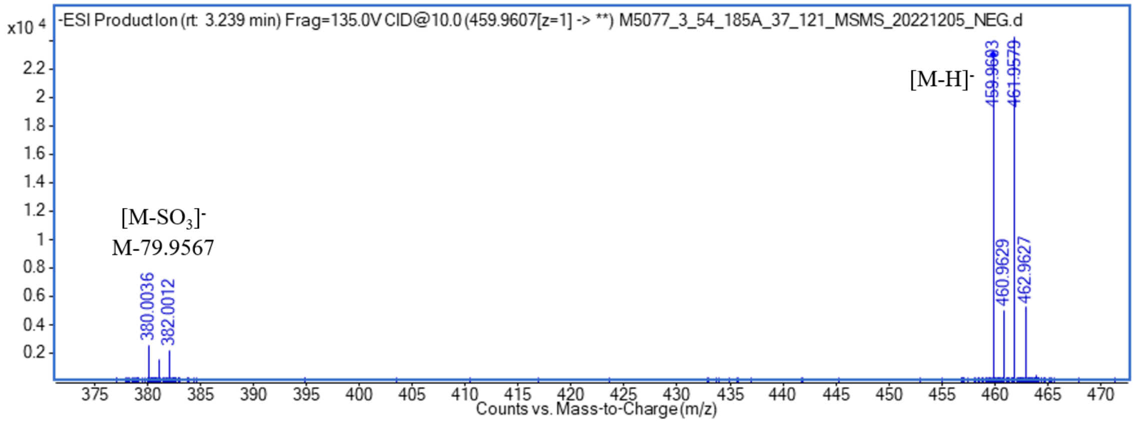

Figure S5. ^{1}H NMR spectrum (600.0 MHz, DMSO-d_6) for compound **6**. The spectrum was acquired over 4 scans.

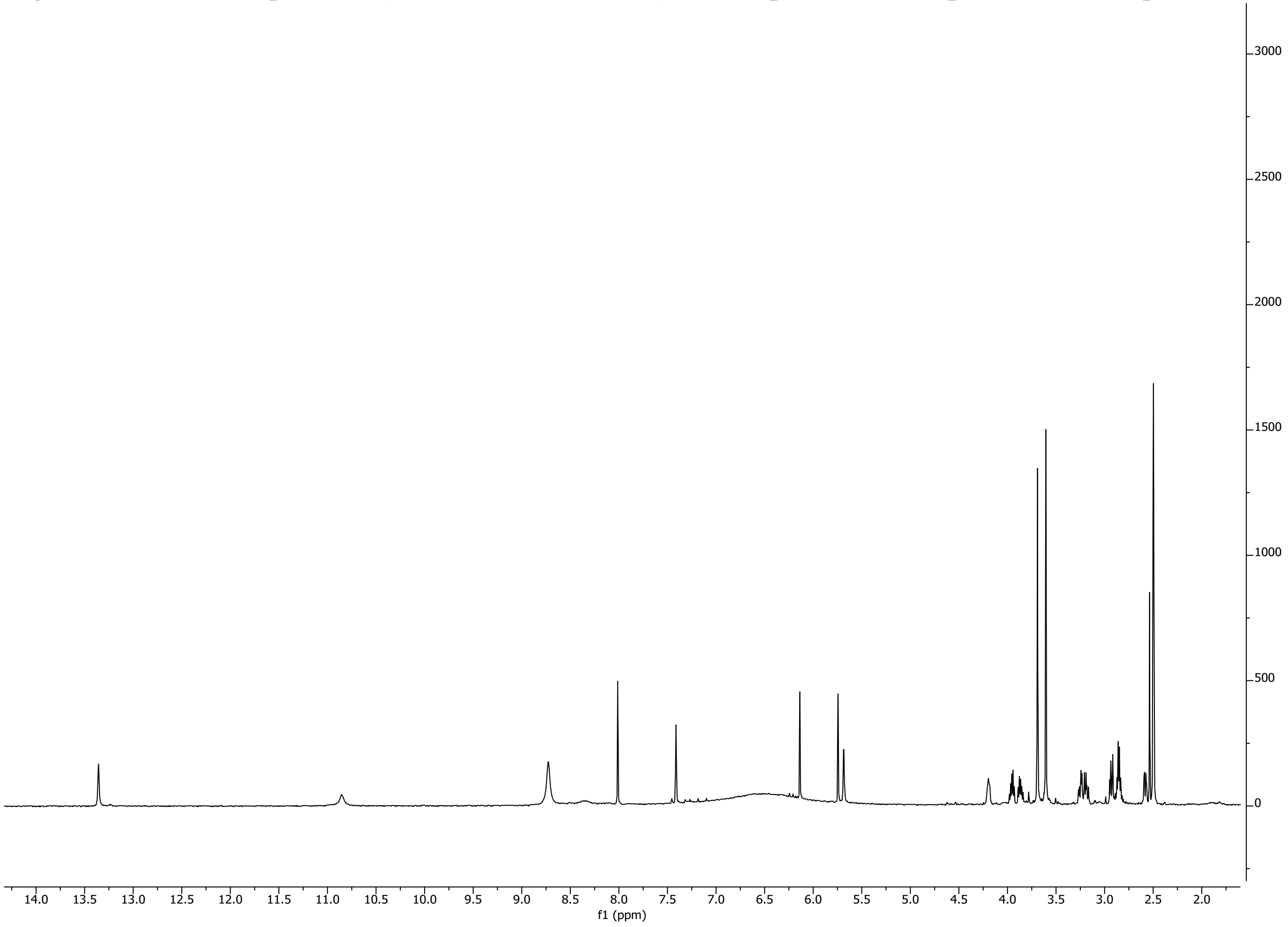

Figure S6. ^{13}C NMR spectrum (150.9 MHz, DMSO-d_6) for compound **6**. The spectrum was acquired over 13000 scans.

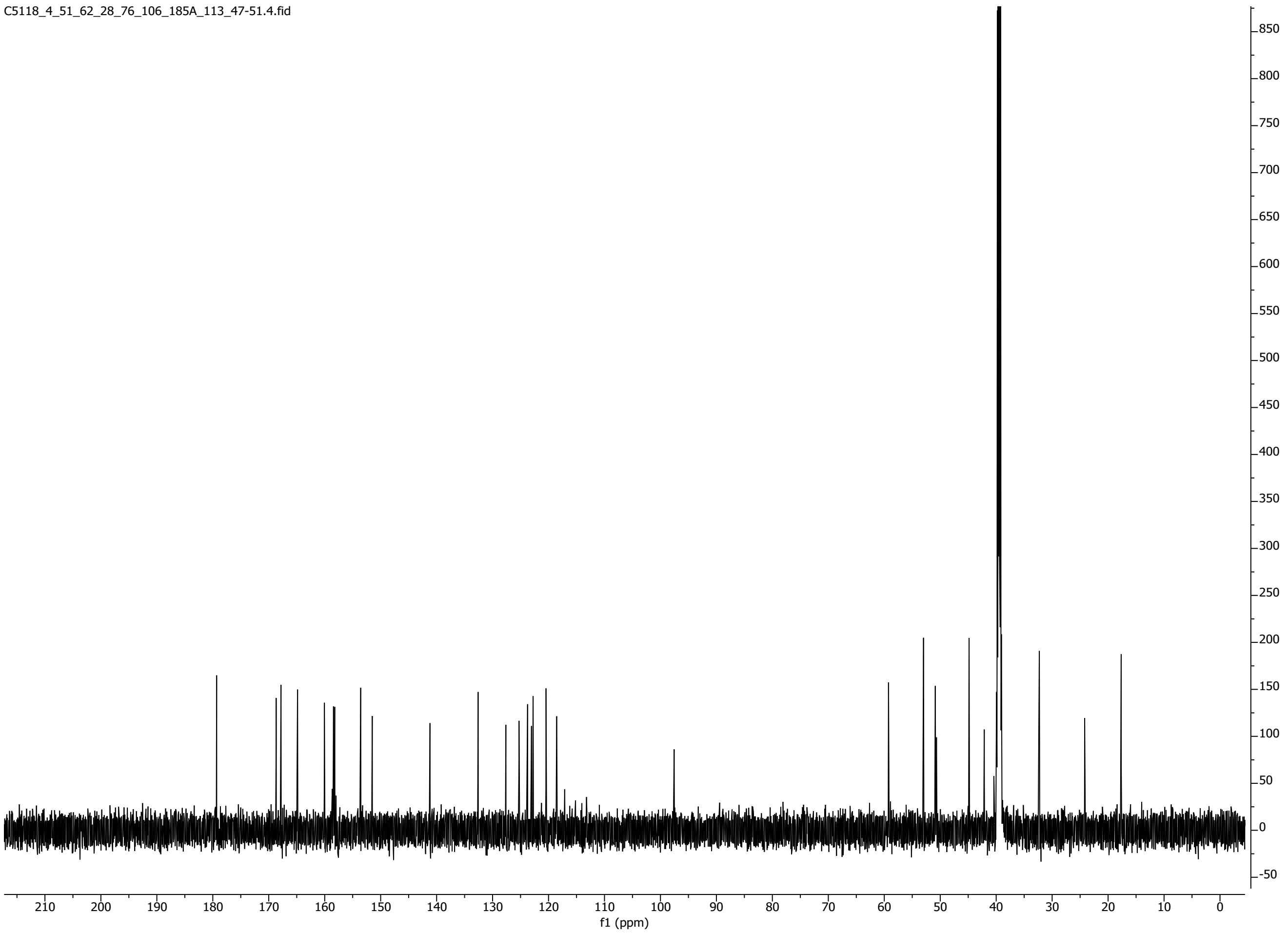

Figure S7. ^{1}H – ^{1}H COSY NMR spectrum (600.0 MHz, DMSO-d_6) for compound **6**. The spectrum was acquired over 32 scans at 256 t1 increments.

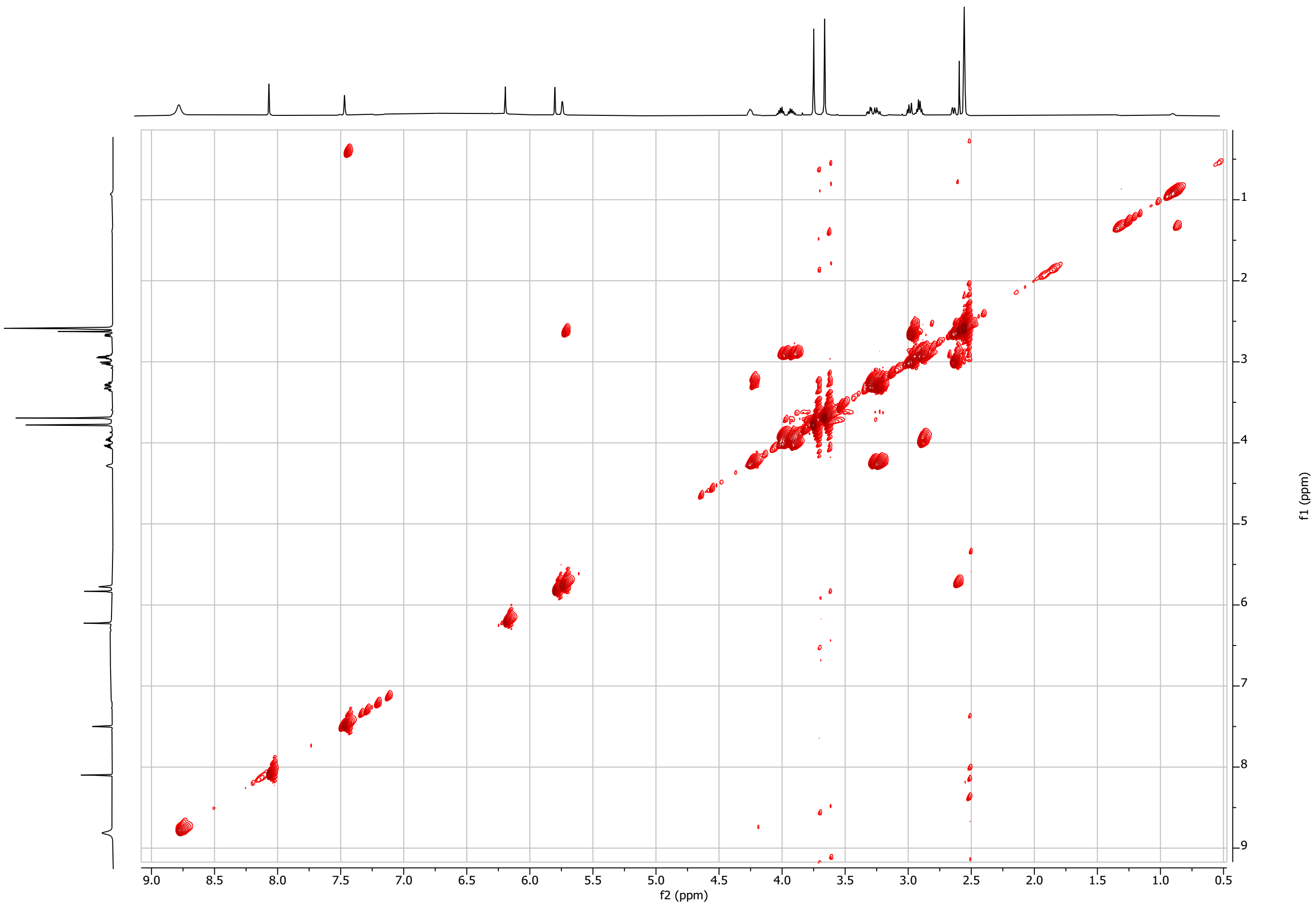

Figure S8. Multiplicity edited ^{1}H – ^{13}C HSQC NMR spectrum (600.0 MHz, DMSO-d_6) for compound **6**. The spectrum was acquired over 32 scans at 256 t1 increments.

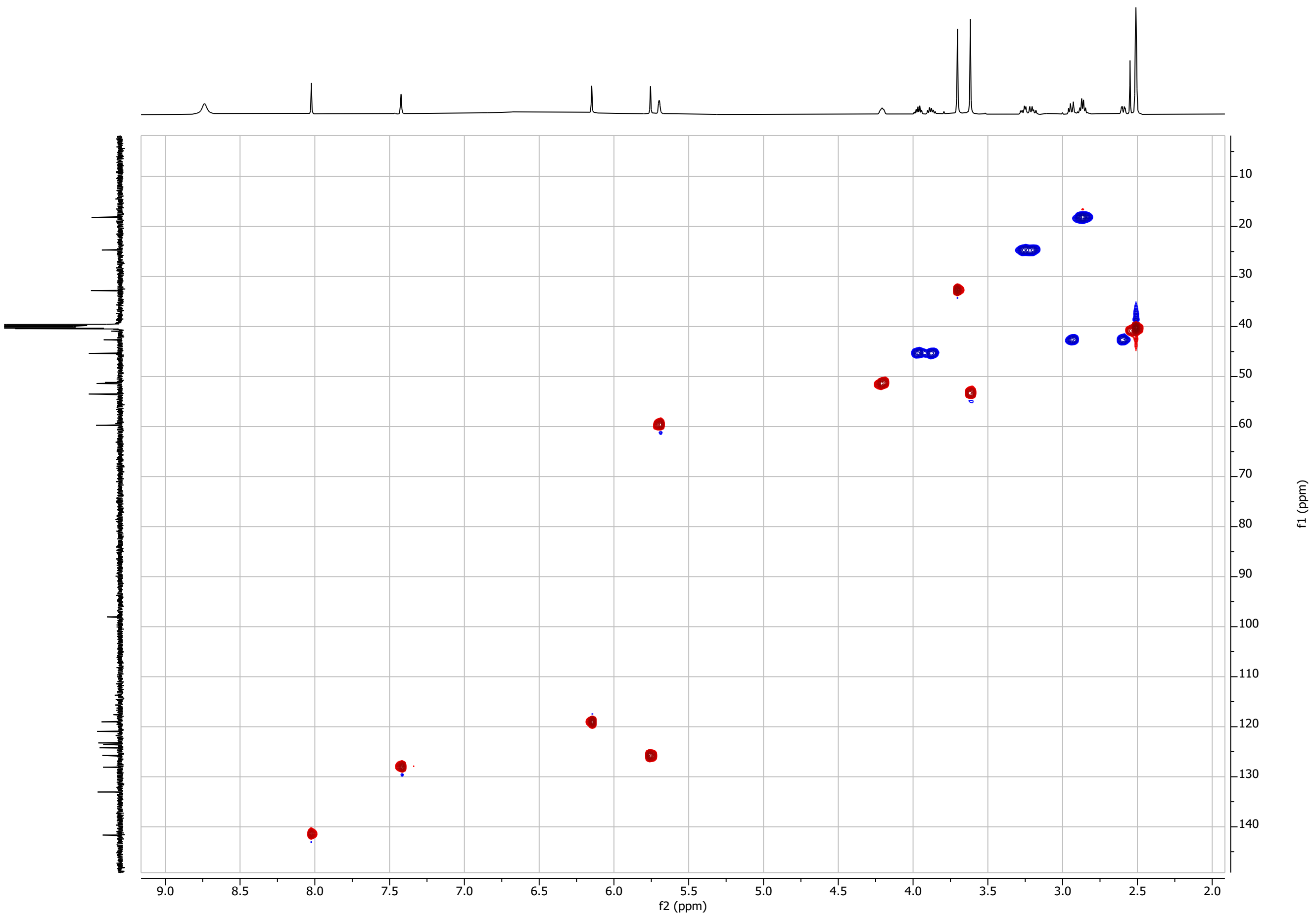

Figure S9. ^{1}H – ^{13}C HMBC NMR spectrum (600.0 MHz, DMSO-d_6) for compound **6**. The spectrum was acquired over 520 scans at 256 t1 increments.

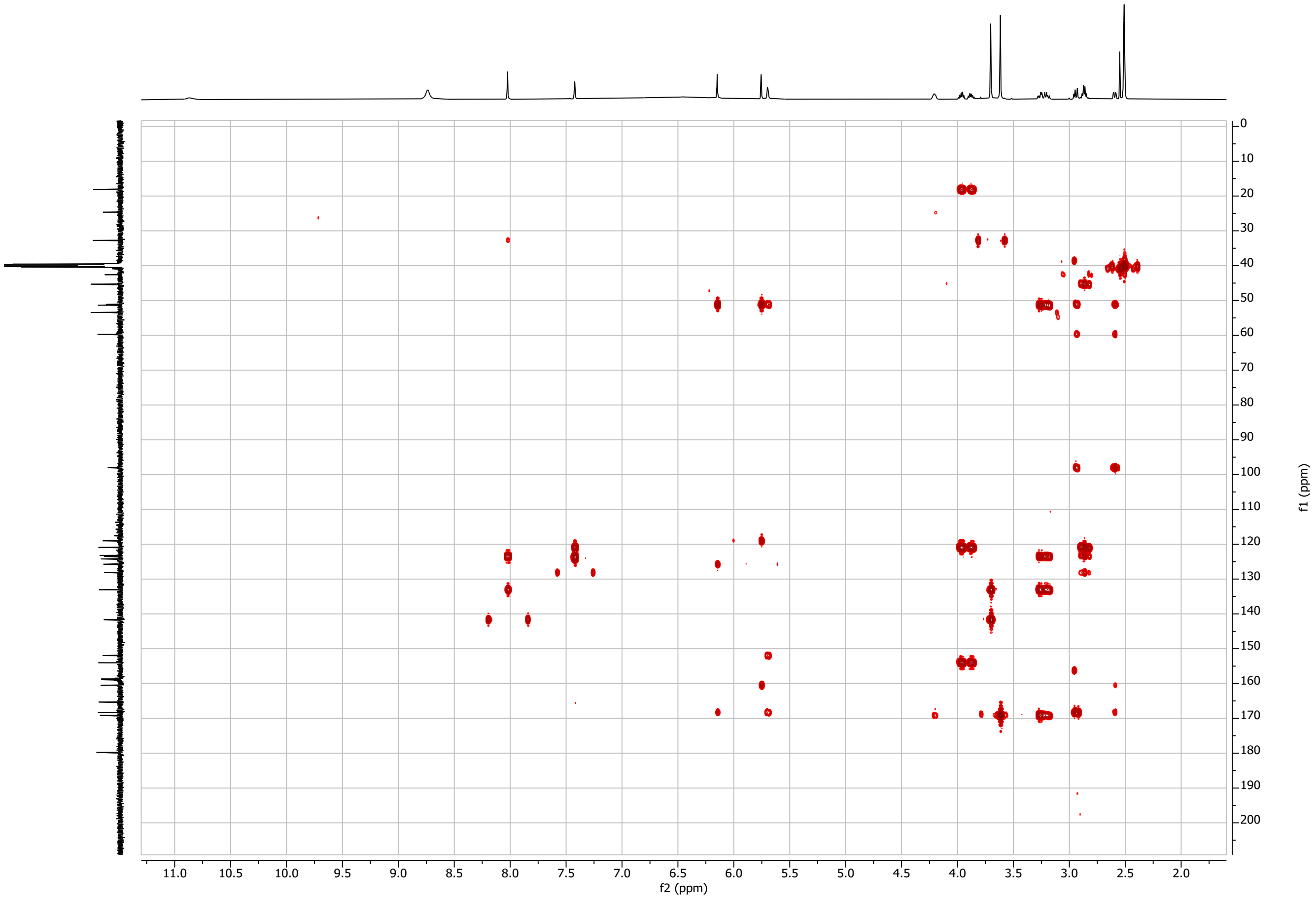

Figure S10. HRESIMS spectrum for compound **6**.

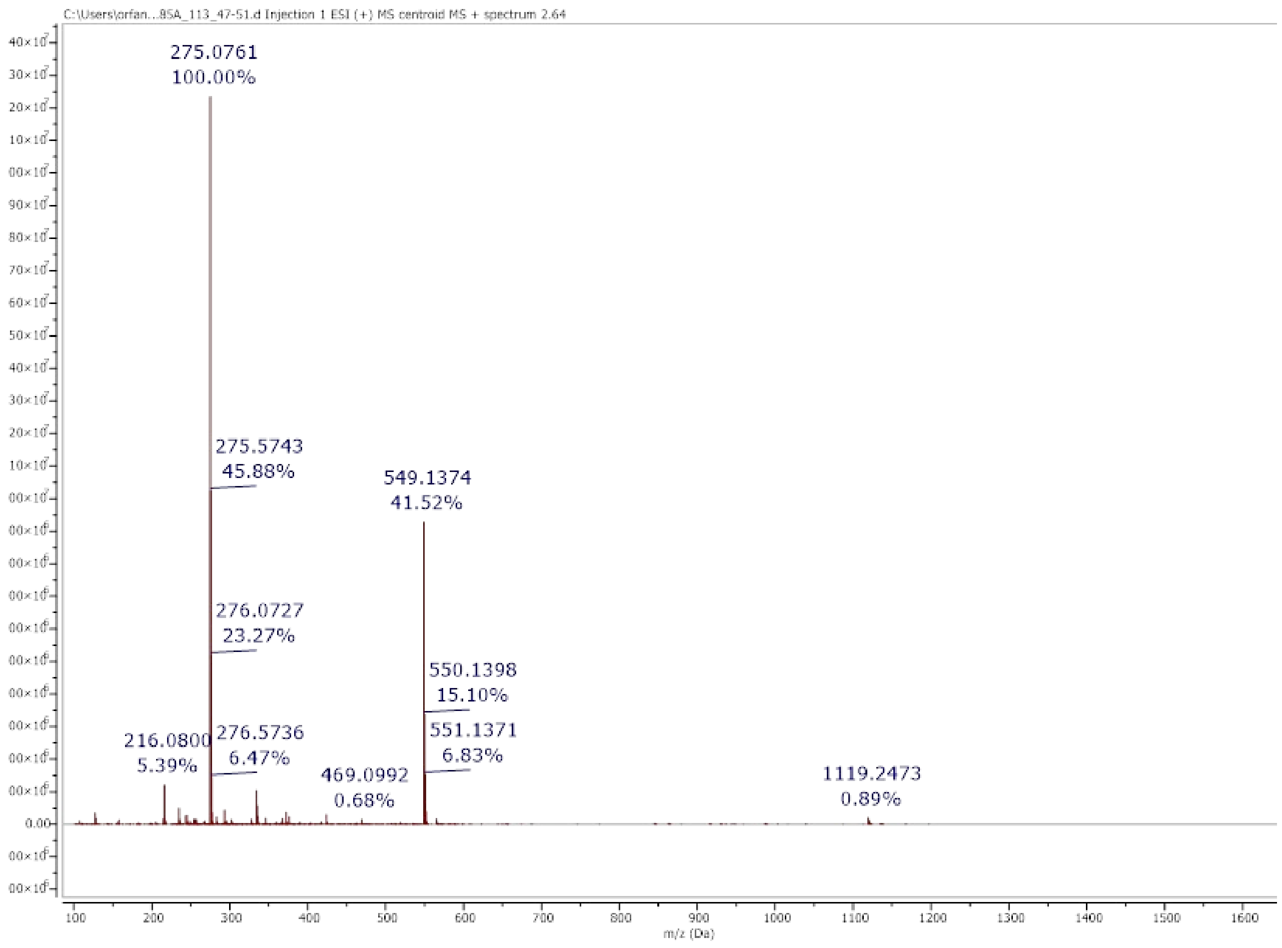

Figure S11. IR spectrum for compound **6**.

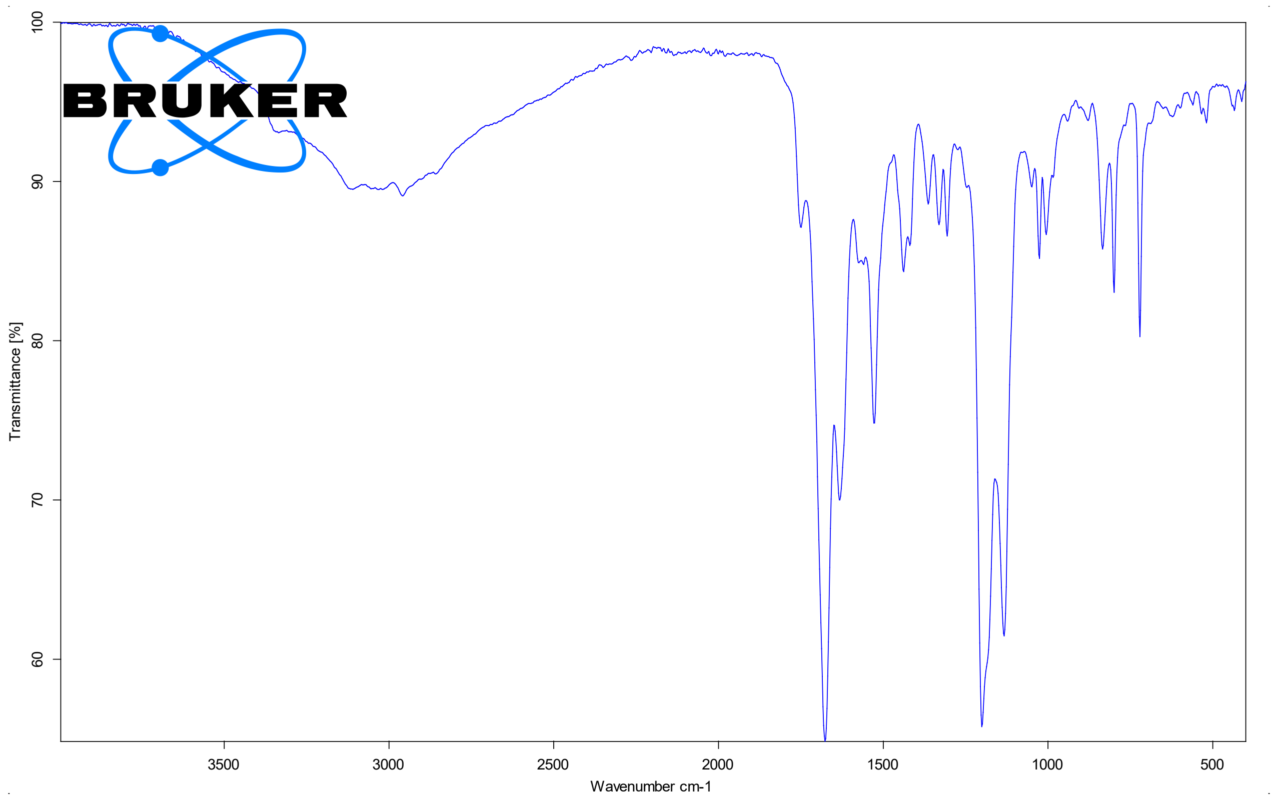

C:\Users\orfanoudakim2\Desktop\Postdoc Frederick\IR\Discos\Discos 1\C5118_4_51_185A_113_47-51.0	C5118_4_51_185A_113_47-51	C5118_4_51_185A_113_47-51	5/11/2022

Page 1/1

Figure S12. ^{1}H NMR spectrum (600.0 MHz, DMSO-d_6) for compound **9**. The spectrum was acquired over 4 scans.

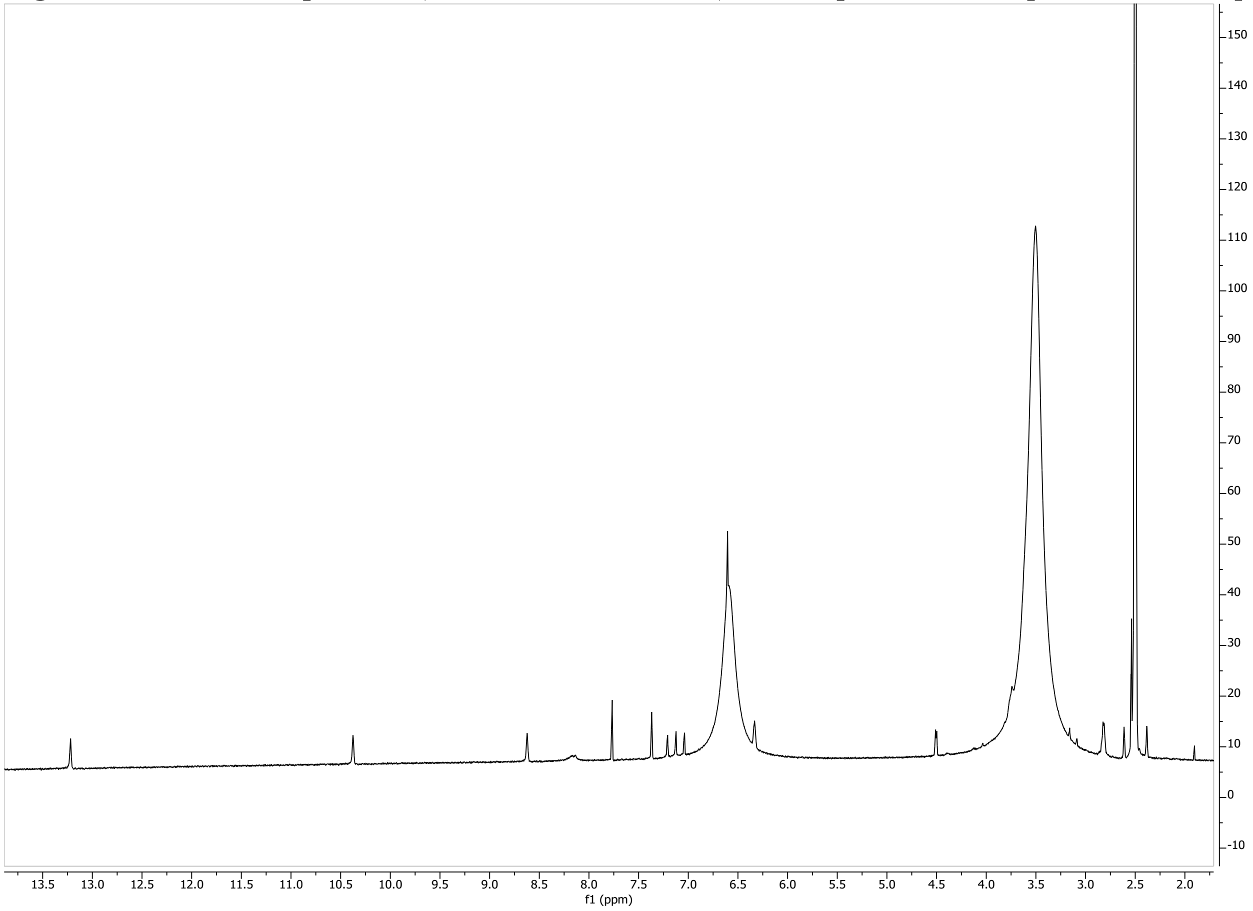

Figure S13. ^{13}C NMR spectrum (150.9 MHz, DMSO-d_6) for compound **9**. The spectrum was acquired over 96000 scans.

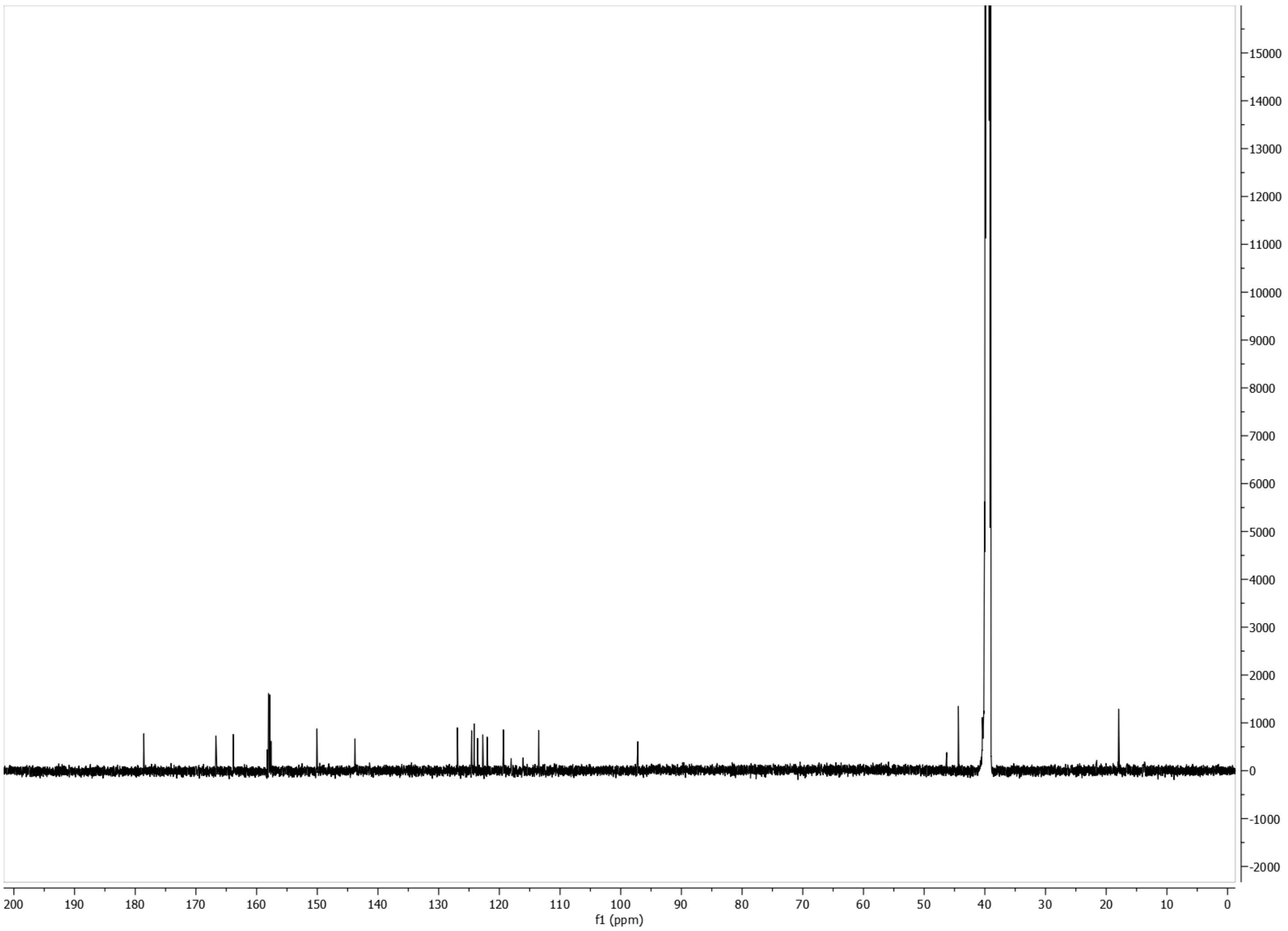

Figure S14. ^{1}H – ^{1}H COSY NMR spectrum (600.0 MHz, DMSO-d_6) for compound **9**. The spectrum was acquired over 32 scans at 256 t1 increments.

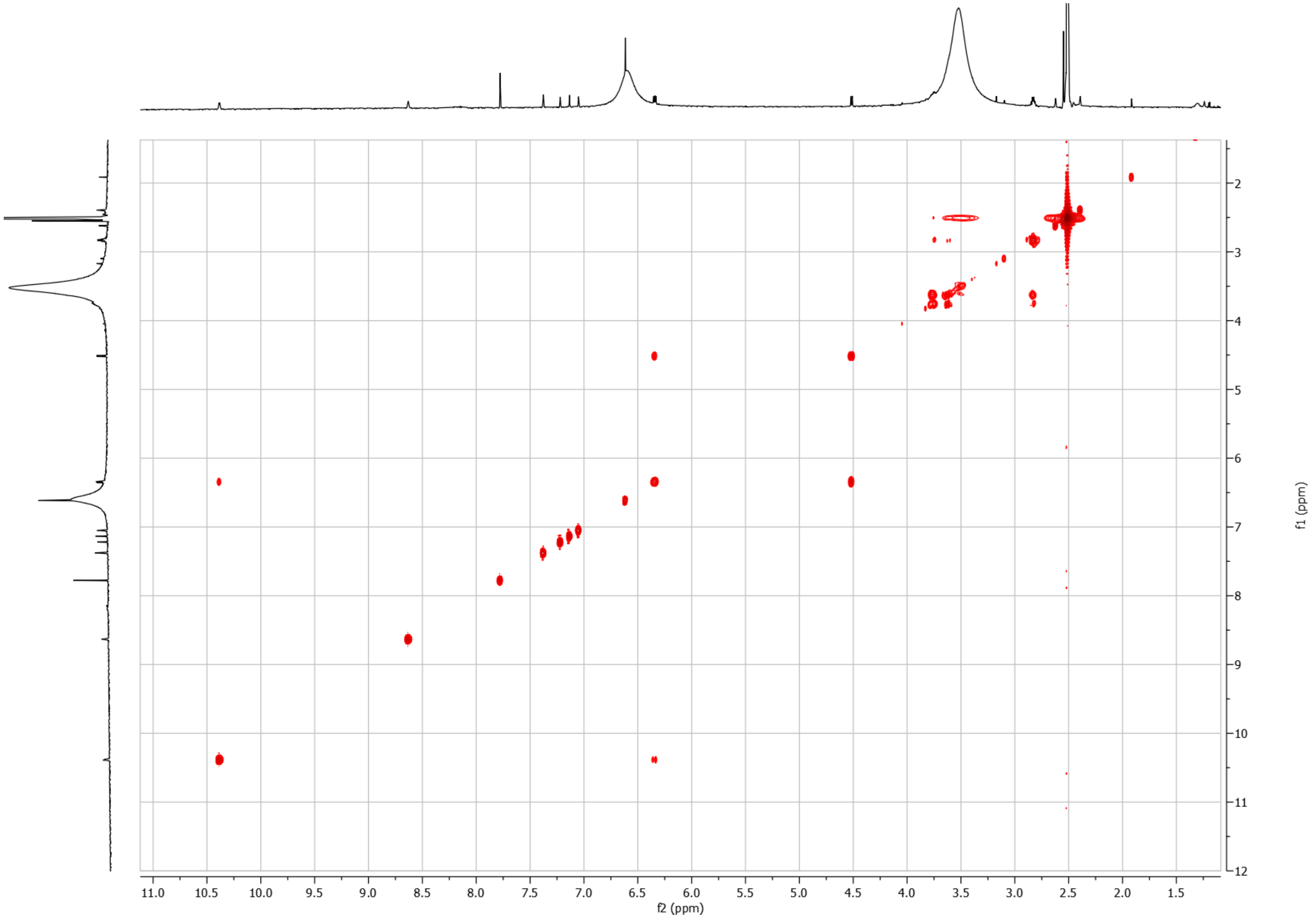

Figure S15. Multiplicity edited ^1H – ^{13}C HSQC NMR spectrum (600.0 MHz, DMSO-d_6) for compound **9**. The spectrum was acquired over 64 scans at 256 t1 increments.

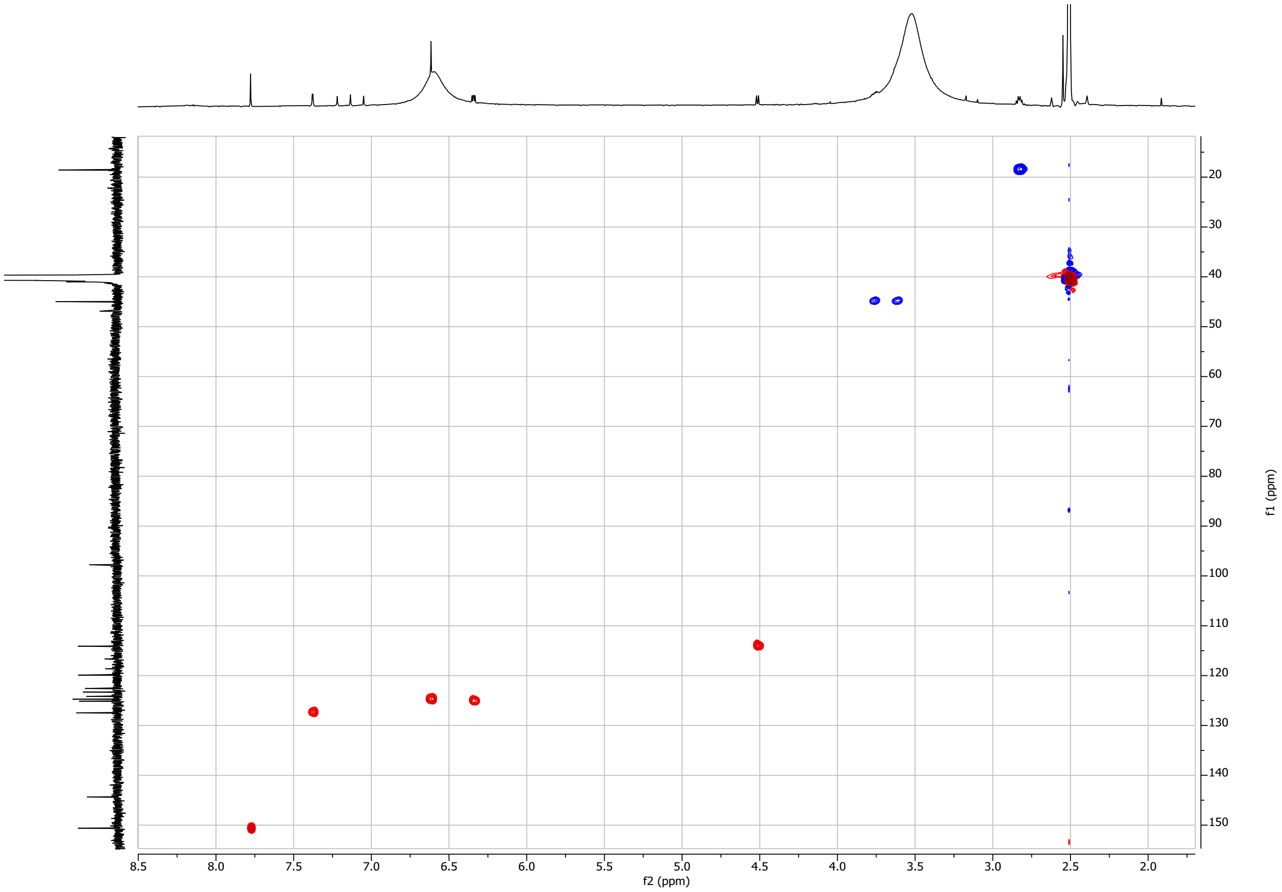

Figure S16. ^{1}H – ^{13}C HMBC NMR spectrum (600.0 MHz, DMSO-d_6) for compound **9**. The spectrum was acquired over 1400 scans at 256 t1 increments.

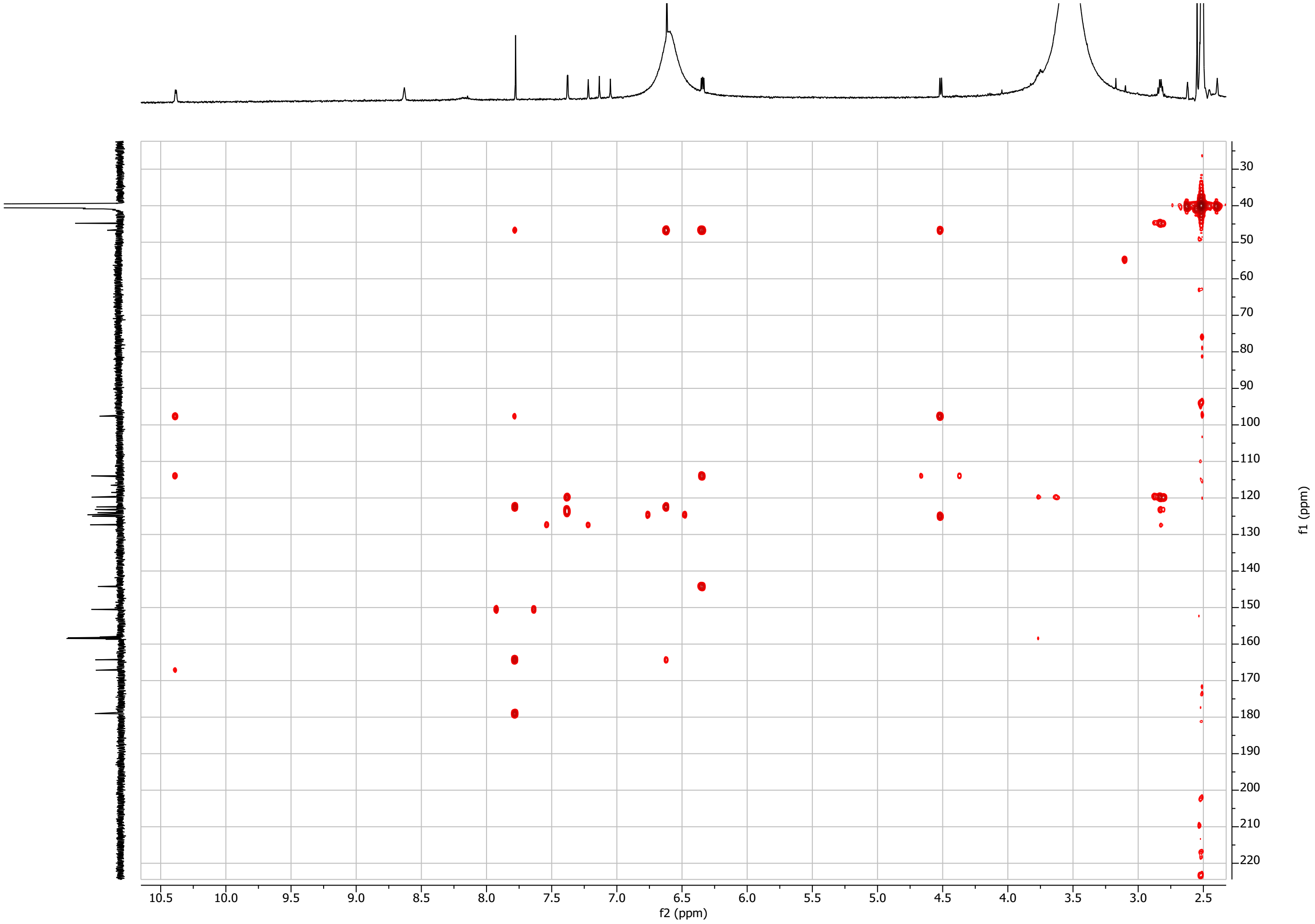

Figure S17. HRESIMS spectrum for compound **9**.

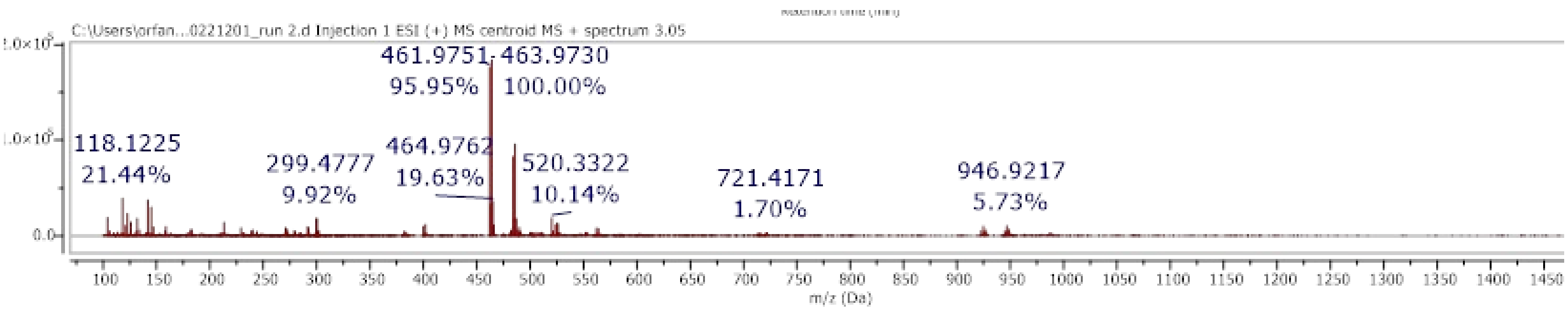

Figure S18. IR spectrum for compound **9**.

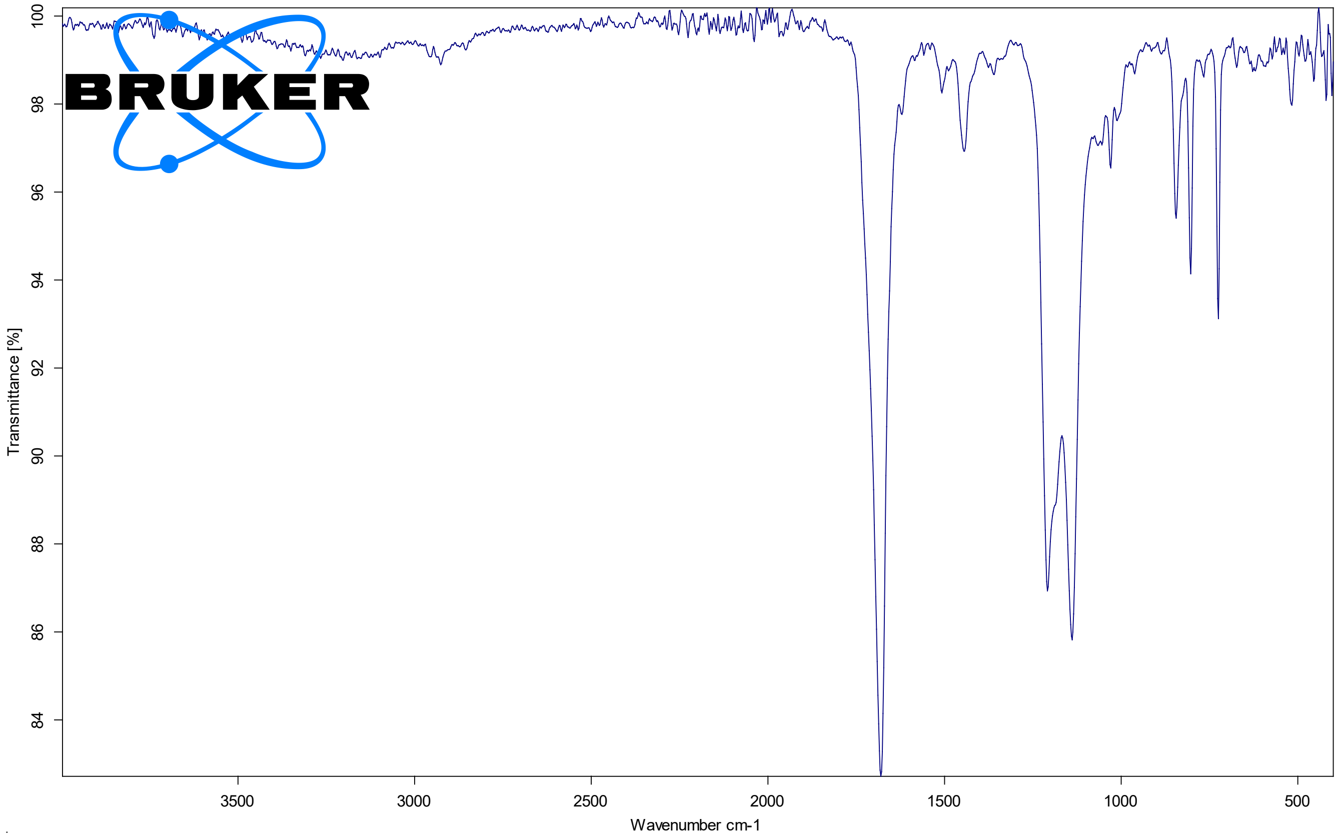

C:\Users\orfanoudakim2\Desktop\Postdoc Frederick\IR\Discos\Discos 0\M5077_3_54_185a_37_121-127conc.0	M5076_3_82_87_185A_159_166conc	Bruker Alpha II		1/29/2022

Page 1/1

Figure S19. ^{1}H NMR spectrum (600.0 MHz, DMSO-d_6) for compound **20**. The spectrum was acquired over 4 scans.

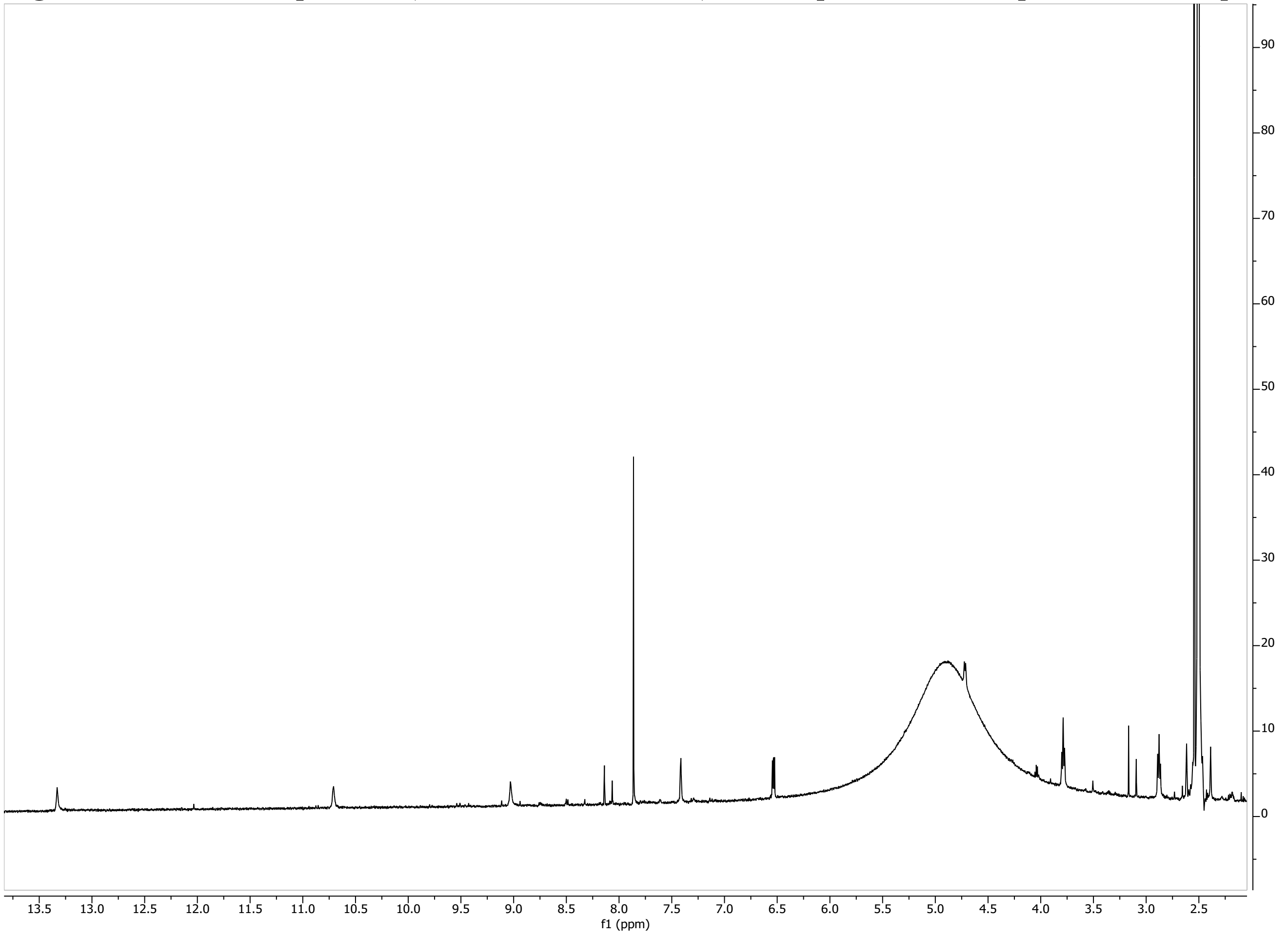

Figure S20. ^{13}C NMR spectrum (150.9 MHz, DMSO-d_6) for compound **20**. The spectrum was acquired over 27000 scans.

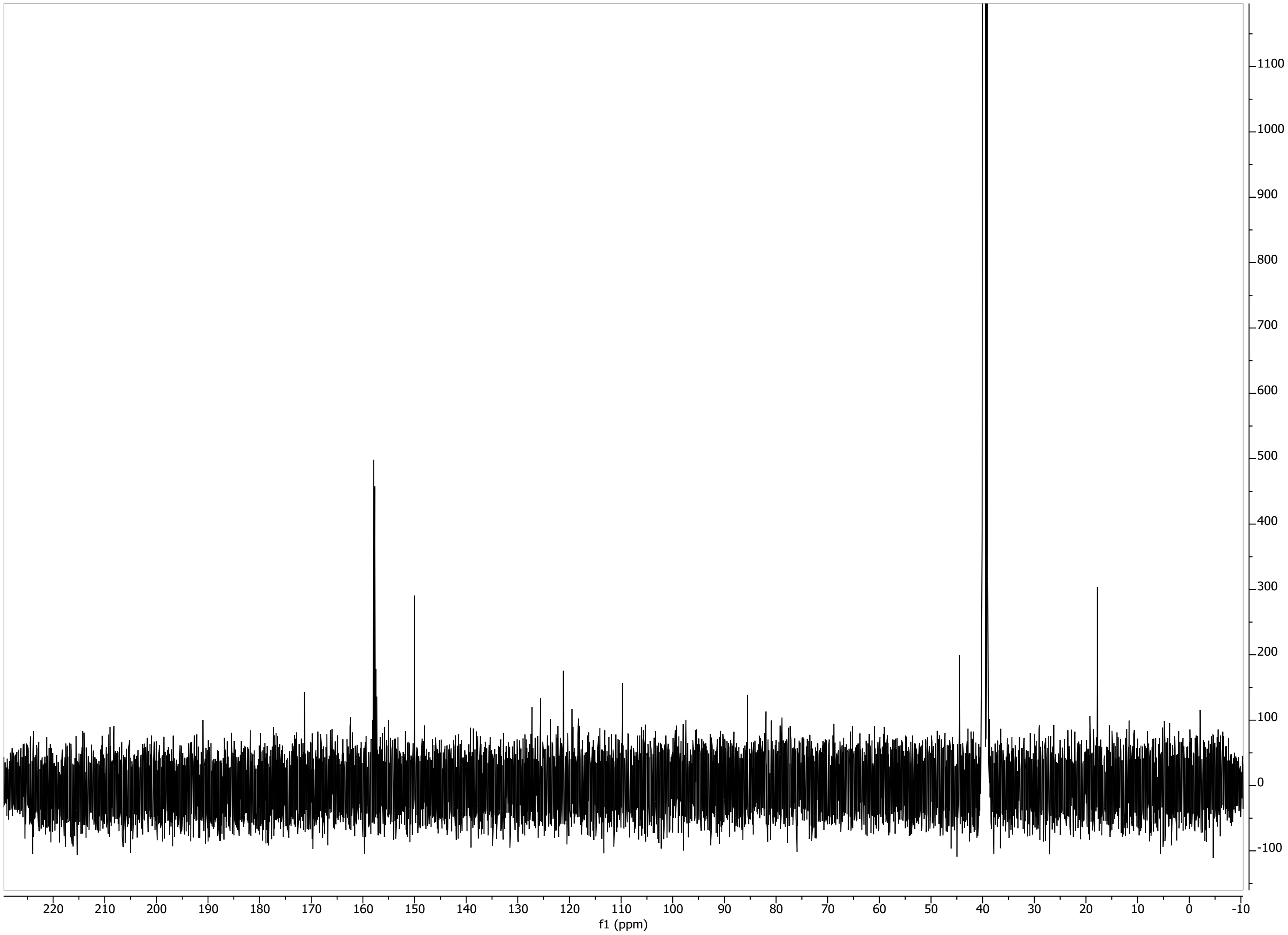

Figure S21. $^{1}H - ^{1}H$ COSY NMR spectrum (600.0 MHz, DMSO-d_6) compound **20**. The spectrum was acquired over 64 scans at 256 t1 increments.

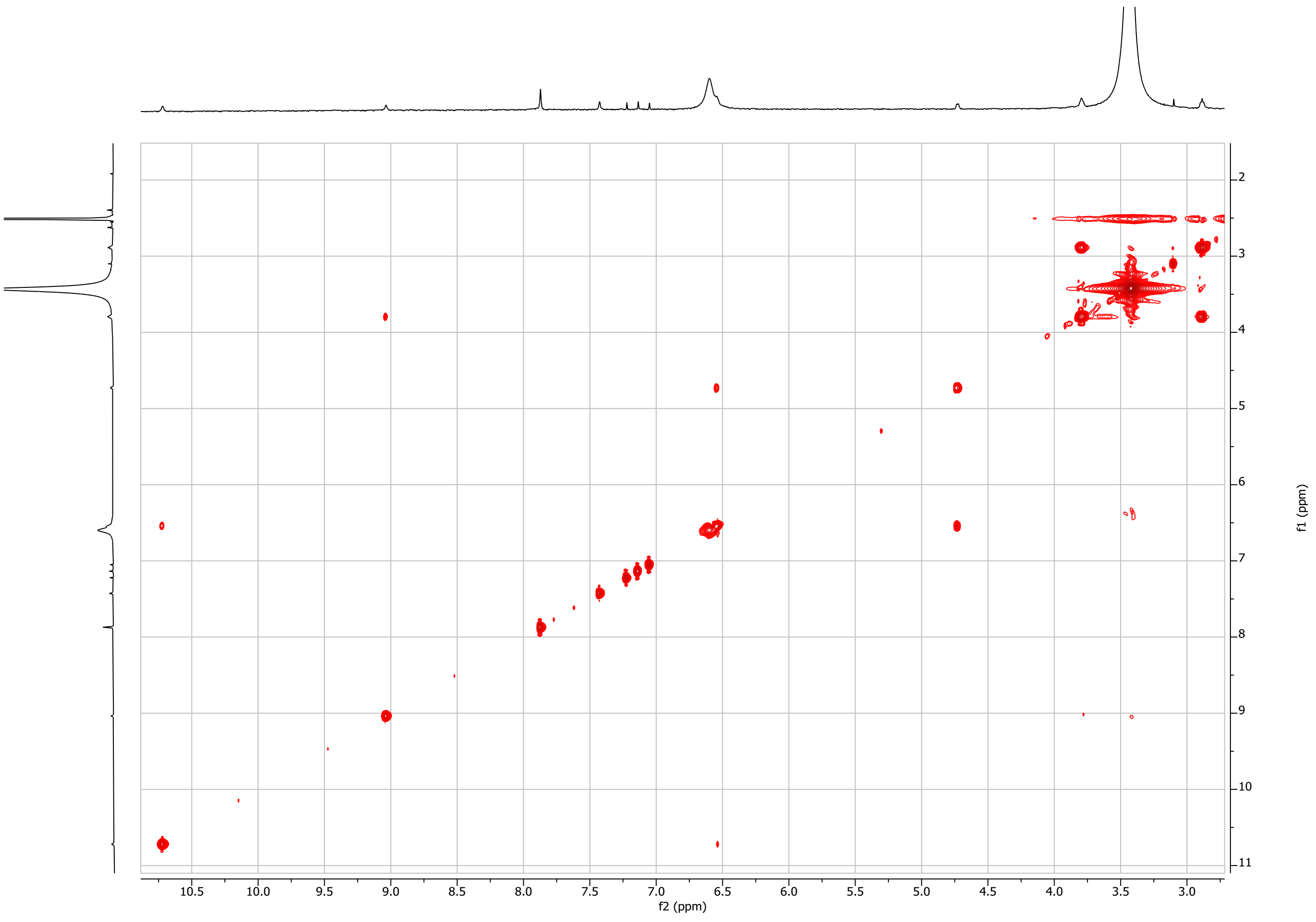

Figure S22. Multiplicity edited ^{1}H – ^{13}C HSQC NMR spectrum (600.0 MHz, DMSO-d_6) for compound **20**. The spectrum was acquired over 32 scans at 256 t1 increments.

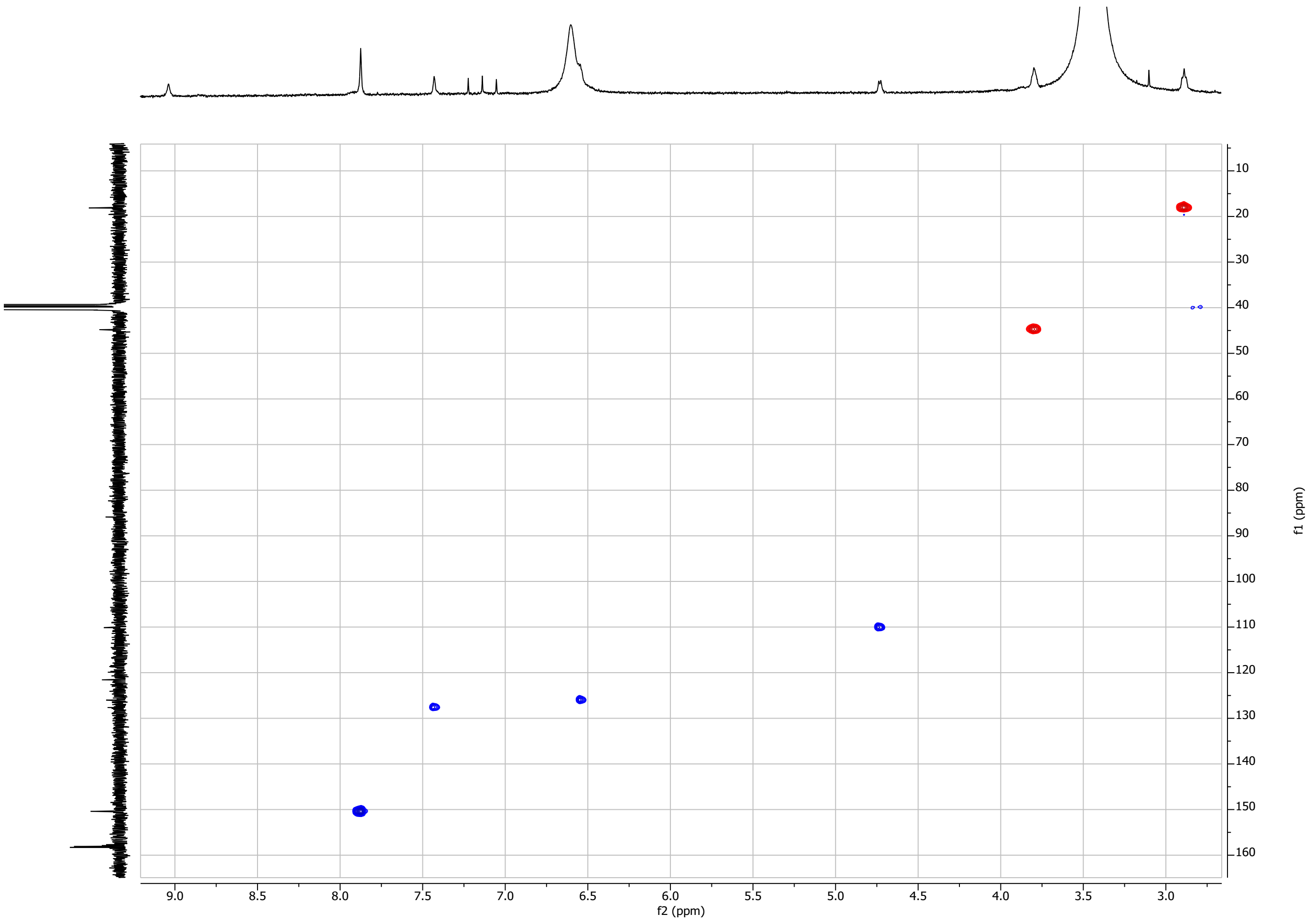

Figure S23. ^{1}H – ^{13}C HMBC NMR spectrum (600.0 MHz, DMSO-d_6) for compound **20**. The spectrum was acquired over 1400 scans at 256 t1 increments.

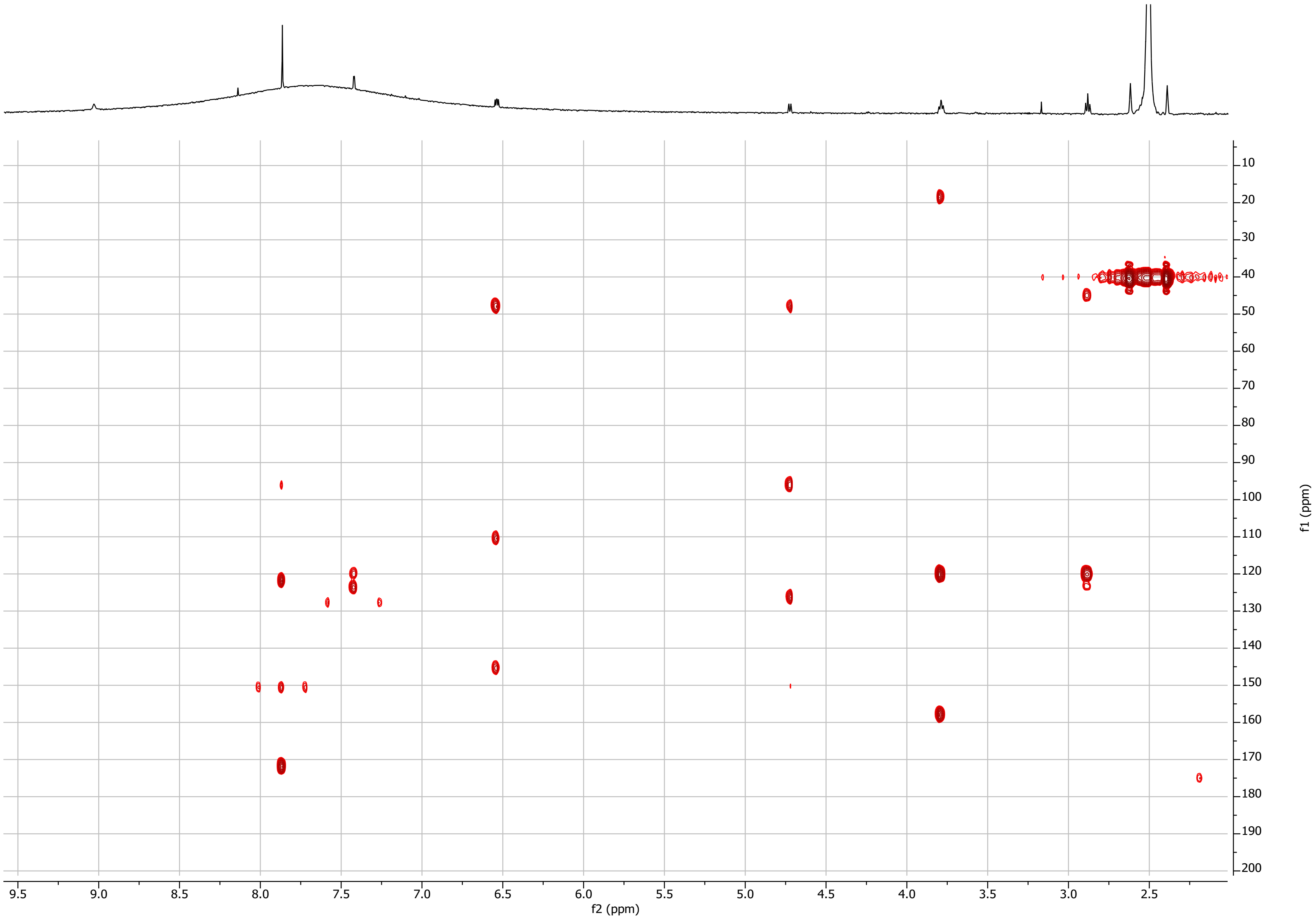

Figure S24. HRESIMS spectrum for compound **20**.

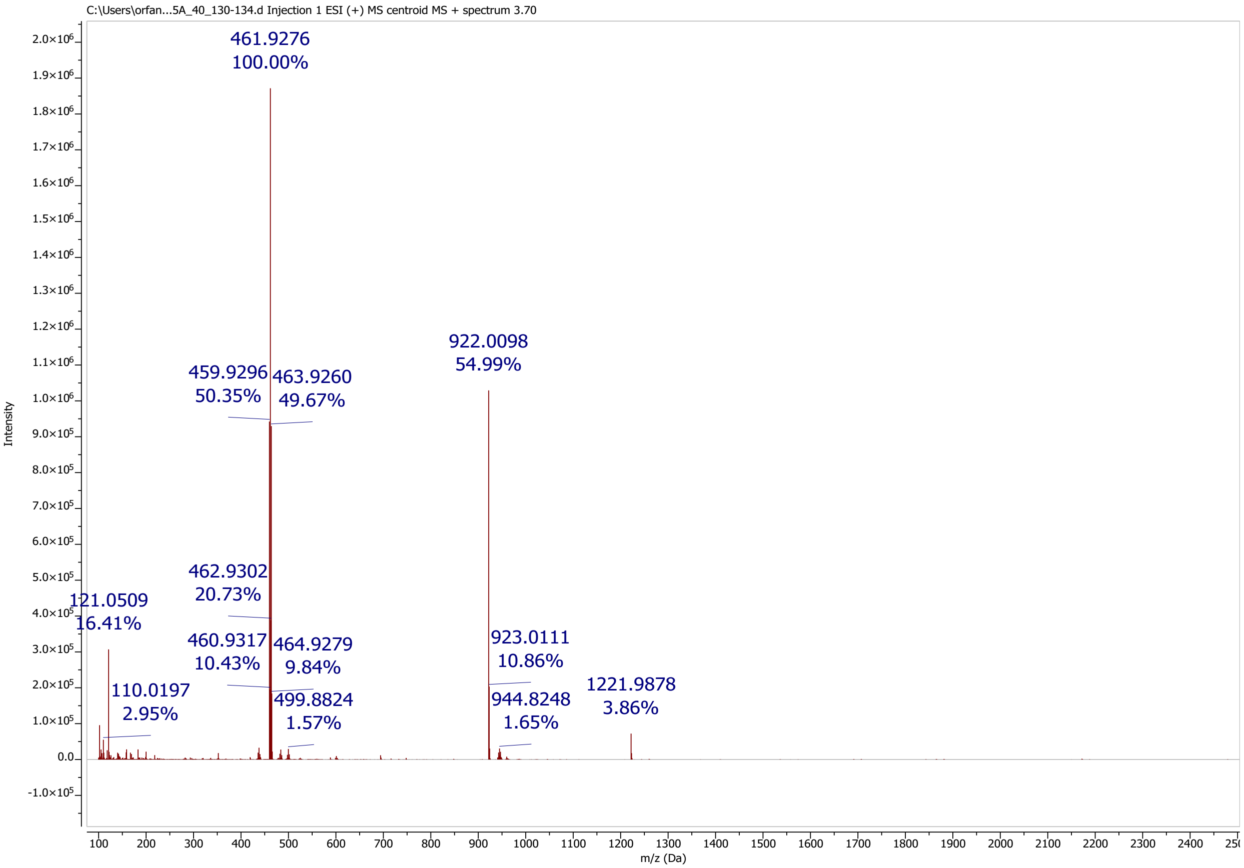

Figure S25. IR spectrum for compound **20**.

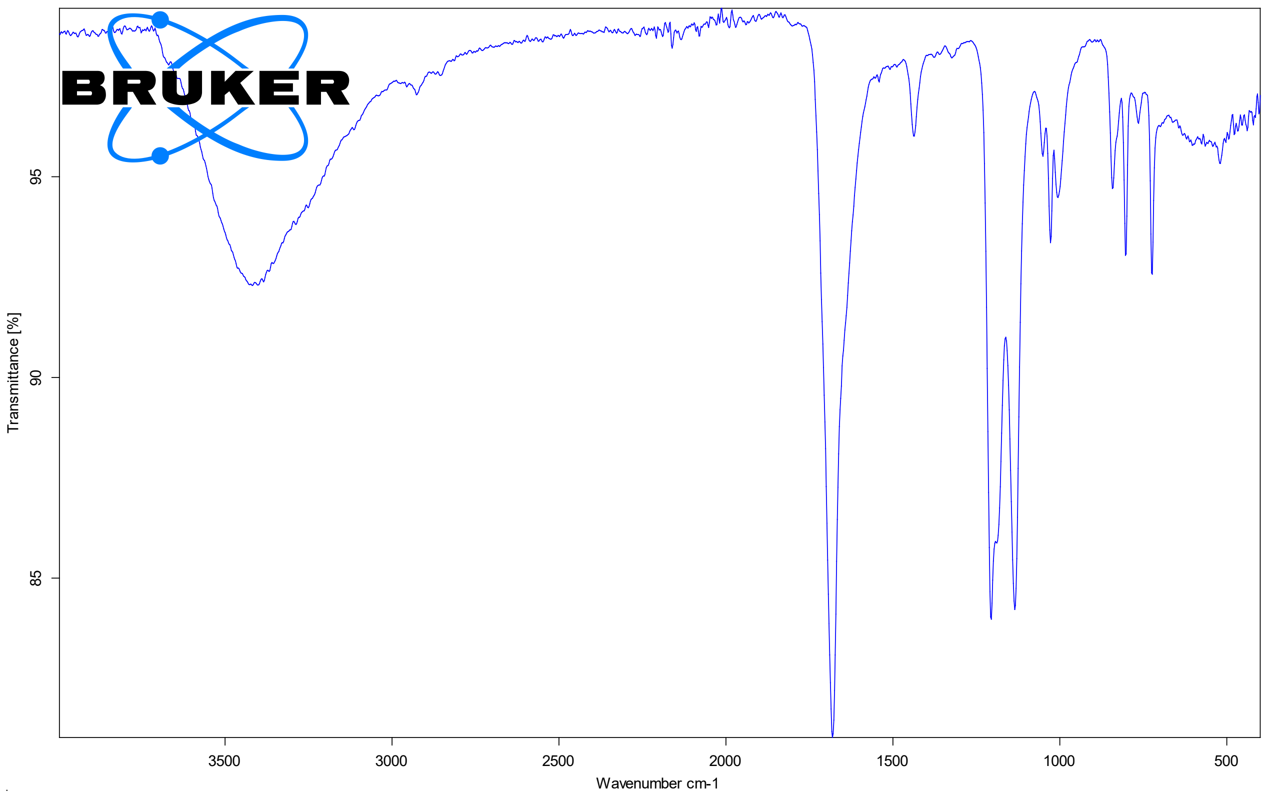

C:\Users\orfanoudakim2\Desktop\Postdoc Frederick\IR\Discos\Discos 2\M5076_3_55_185A_40_130-134.0 M5076_3_55_185A_40_130-134 M5076_3_55_185A_40_130-134	7/14/2022

Page 1/1

Figure S26. ^{1}H NMR spectrum (600.0 MHz, DMSO-d_6) for compound **18**. The spectrum was acquired over 4 scans.

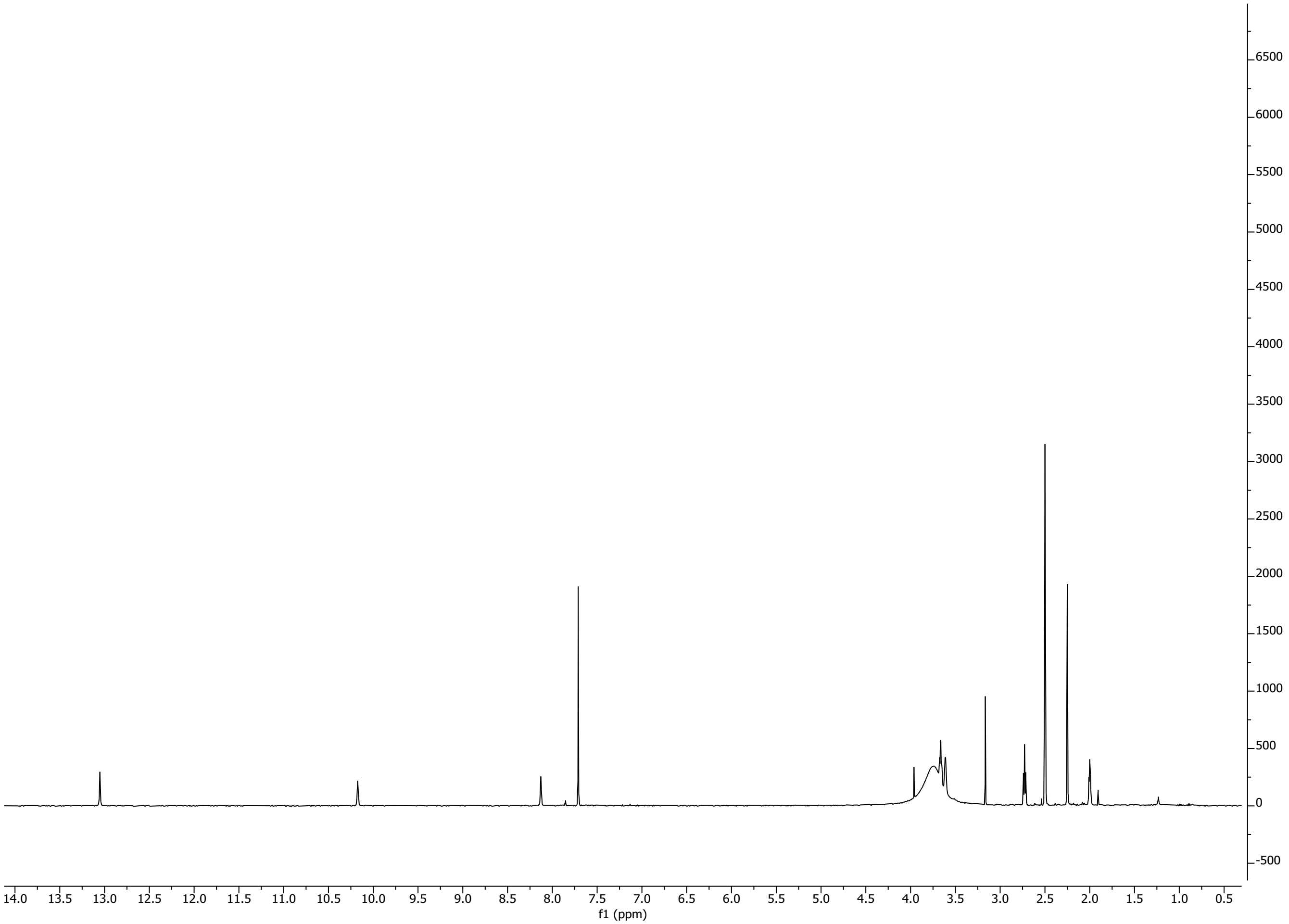

Figure S27. ^{13}C NMR spectrum (150.9 MHz, DMSO-d_6) for compound **18**. The spectrum was acquired over 10000 scans.

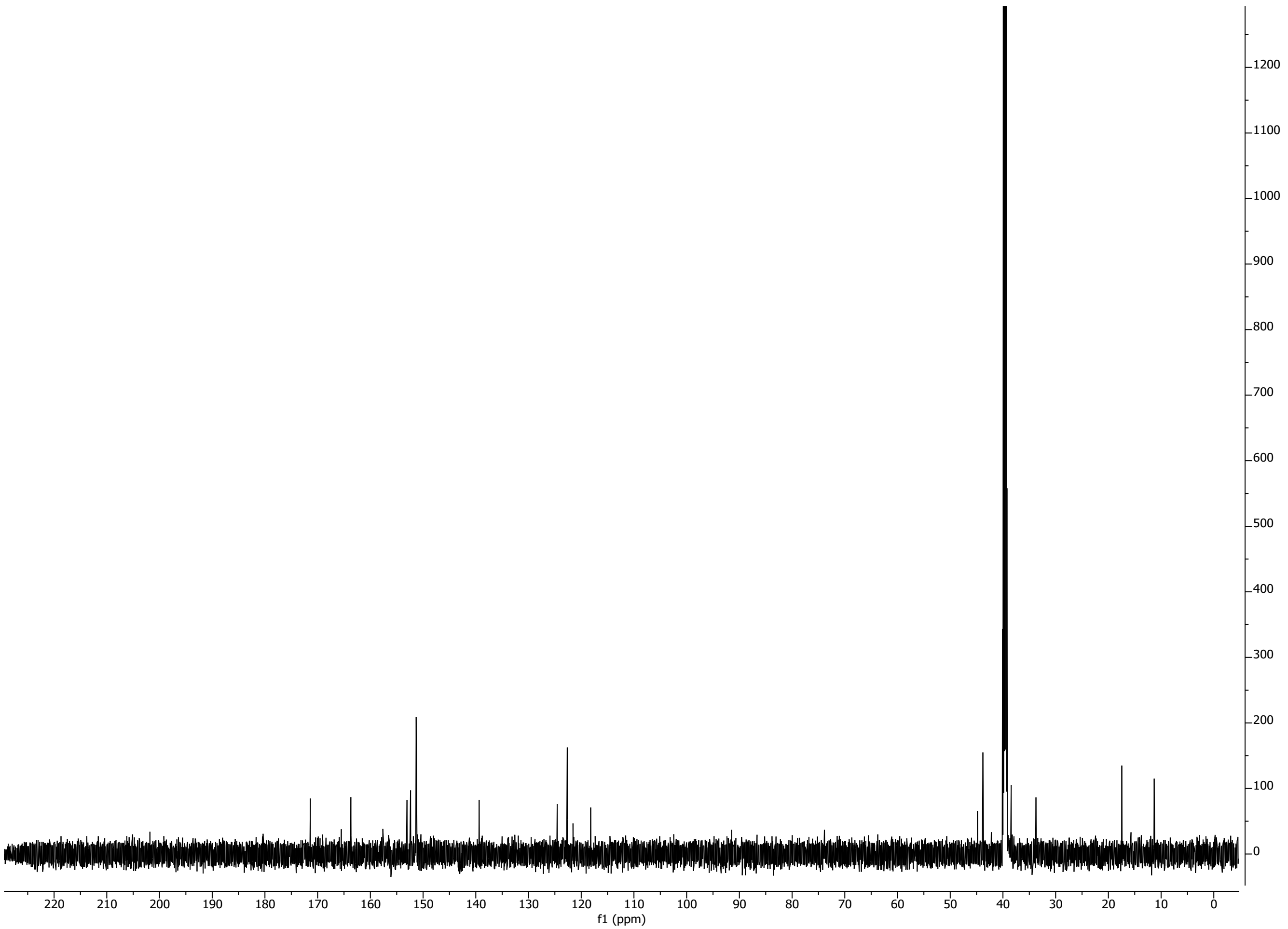

Figure S28. ^{1}H – ^{1}H COSY NMR spectrum (600.0 MHz, DMSO-d_6) for compound **18**. The spectrum was acquired over 8 scans at 256 t1 increments.

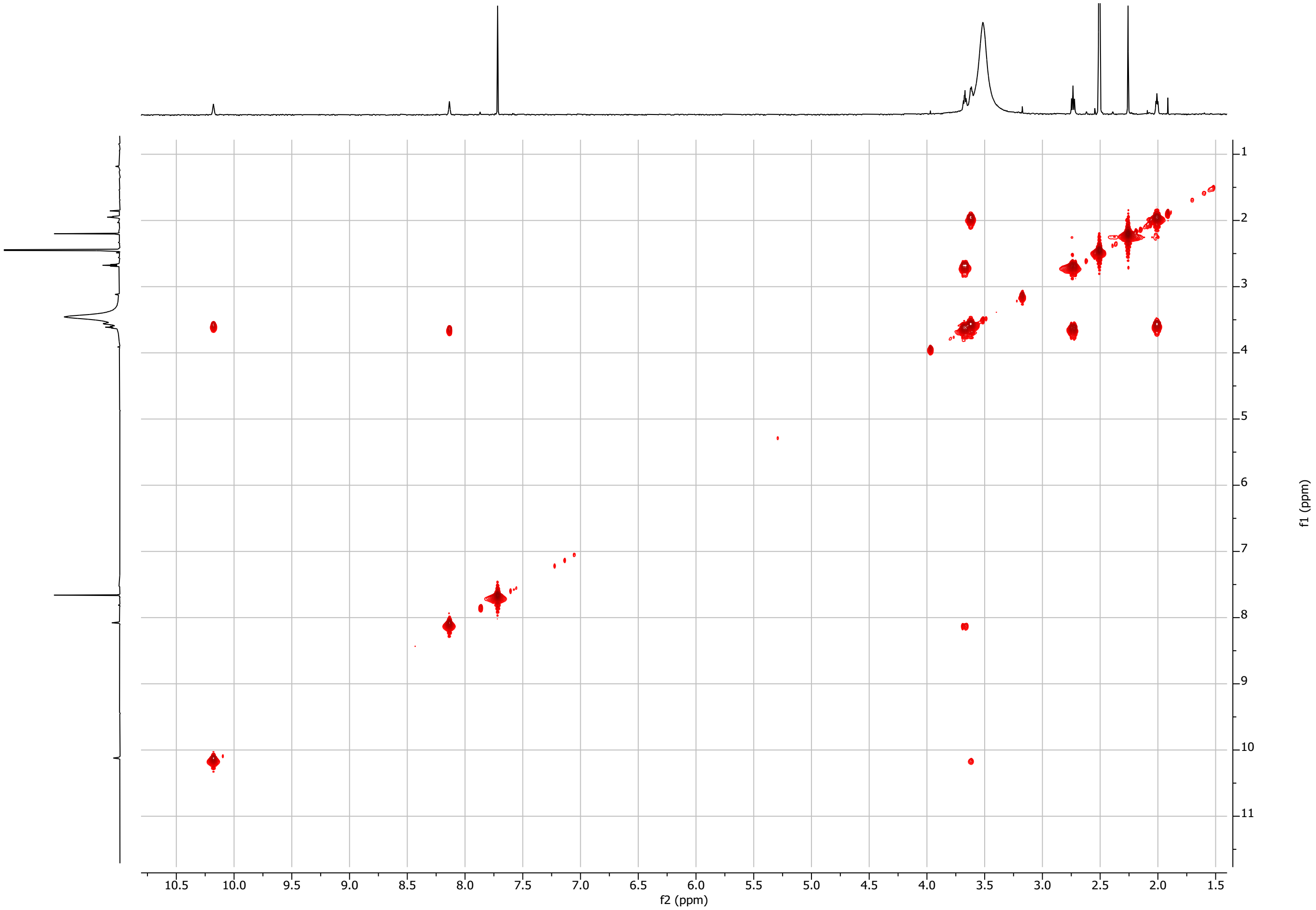

Figure S29. Multiplicity edited ^{1}H – ^{13}C HSQC NMR spectrum (600.0 MHz, DMSO-d_6) for compound **18**. The spectrum was acquired over 16 scans at 256 t1 increments.

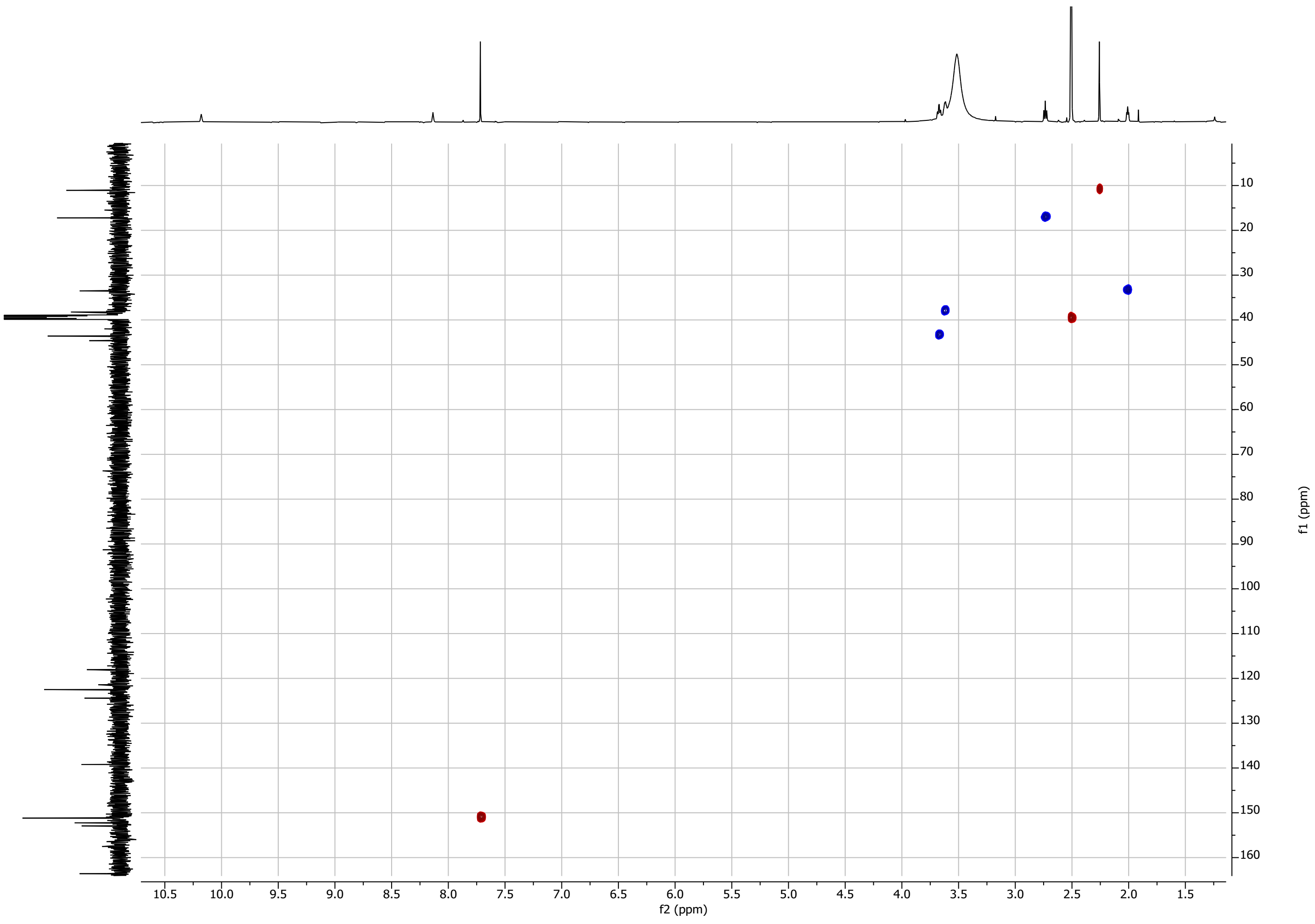

Figure S30. ^{1}H – ^{13}C HMBC NMR spectrum (600.0 MHz, DMSO-d_6) for compound **18**. The spectrum was acquired over 600 scans at 256 t1 increments.

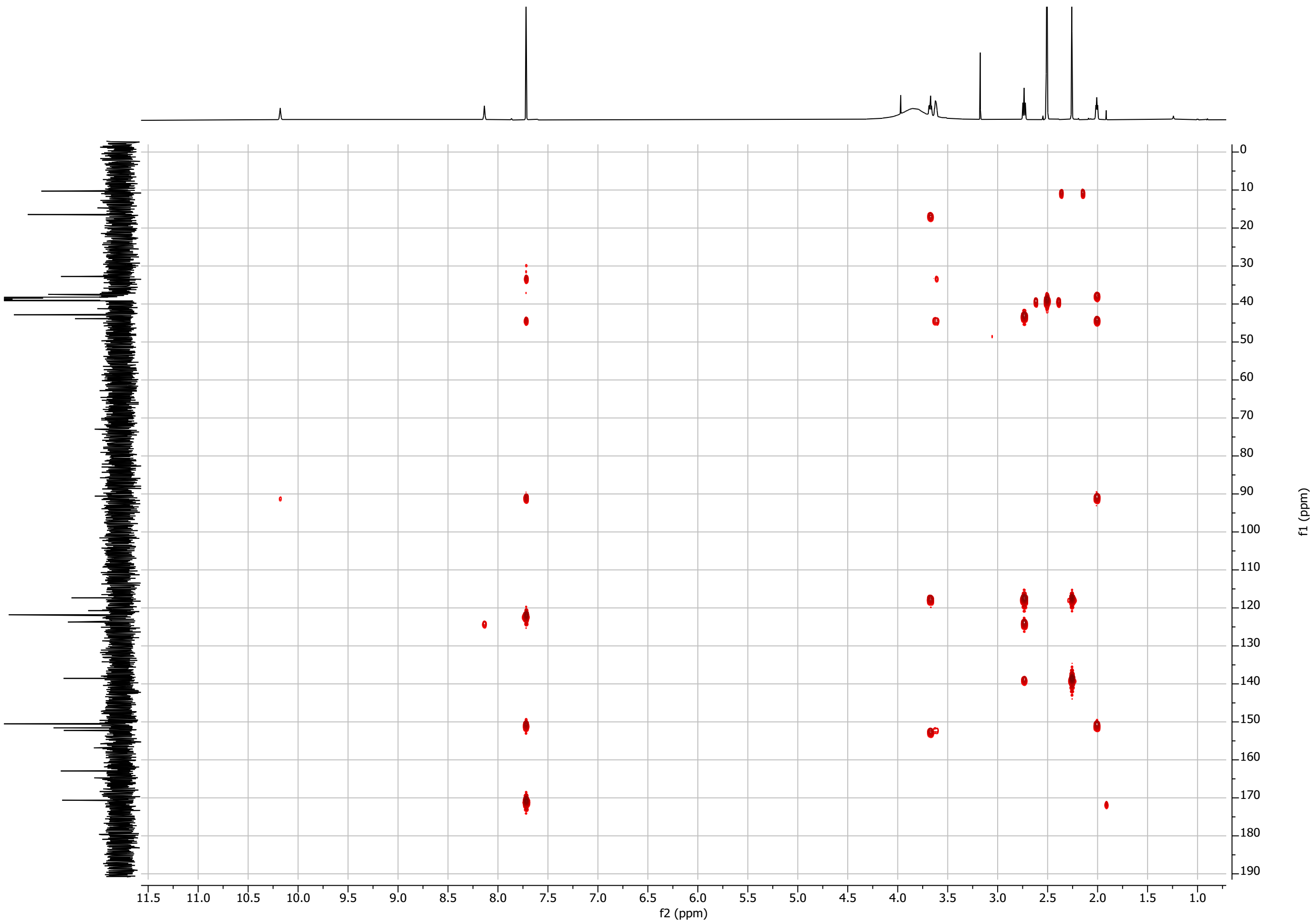

Figure S31. HRESIMS spectrum for compound **18**.

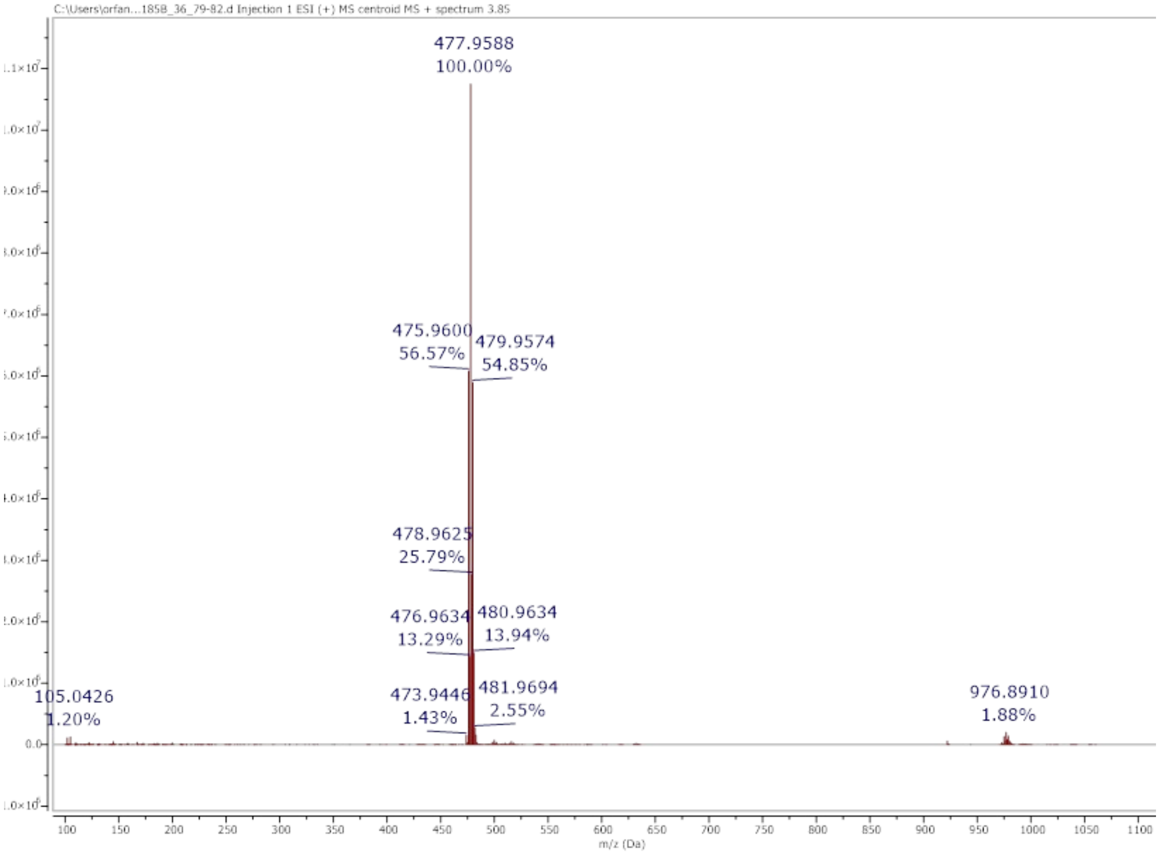

Figure S32. IR spectrum for compound **18**.

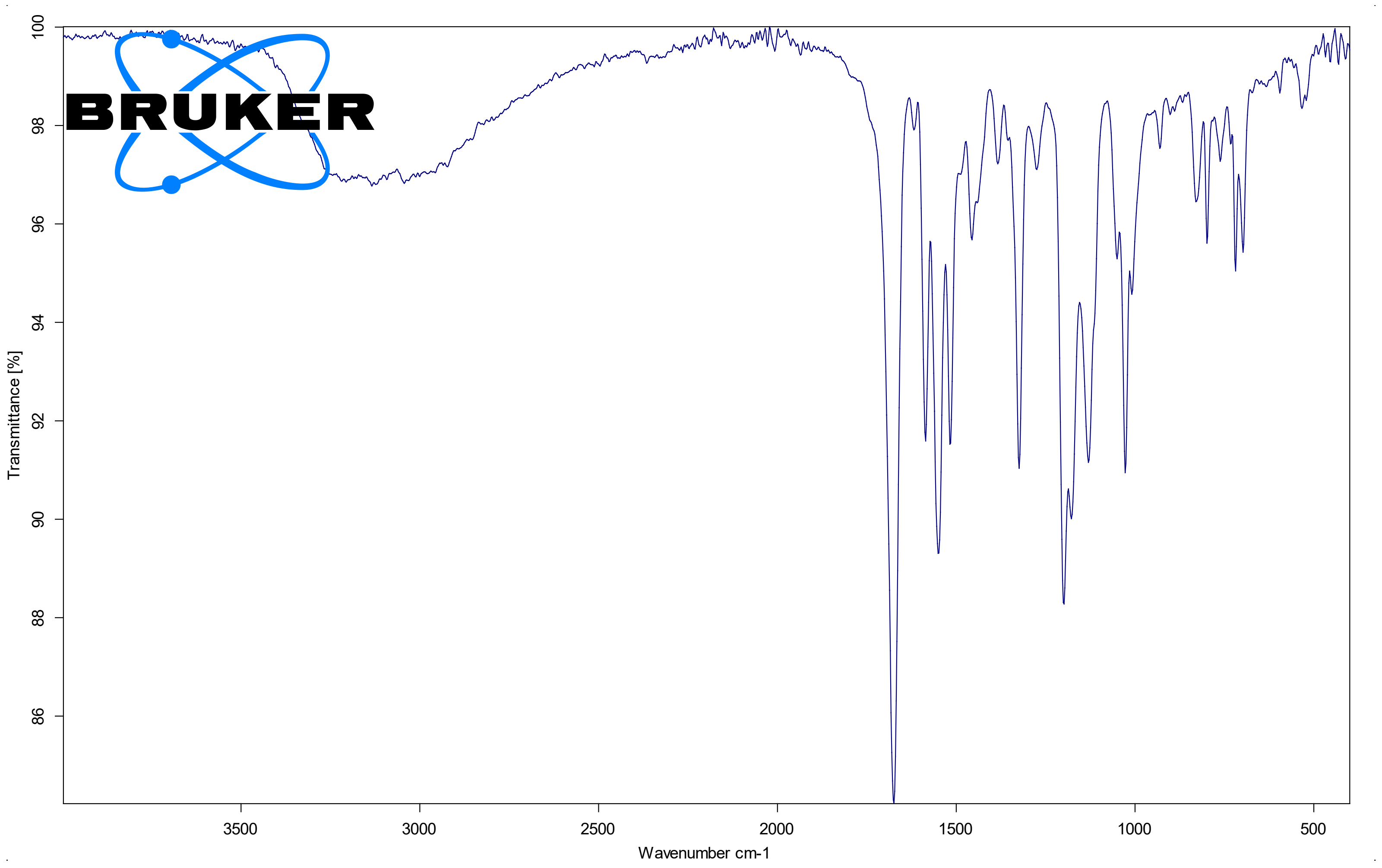

Figure S33. ^{1}H NMR spectrum (600.0 MHz, DMSO-d_6) for compound **23**. The spectrum was acquired over 4 scans.

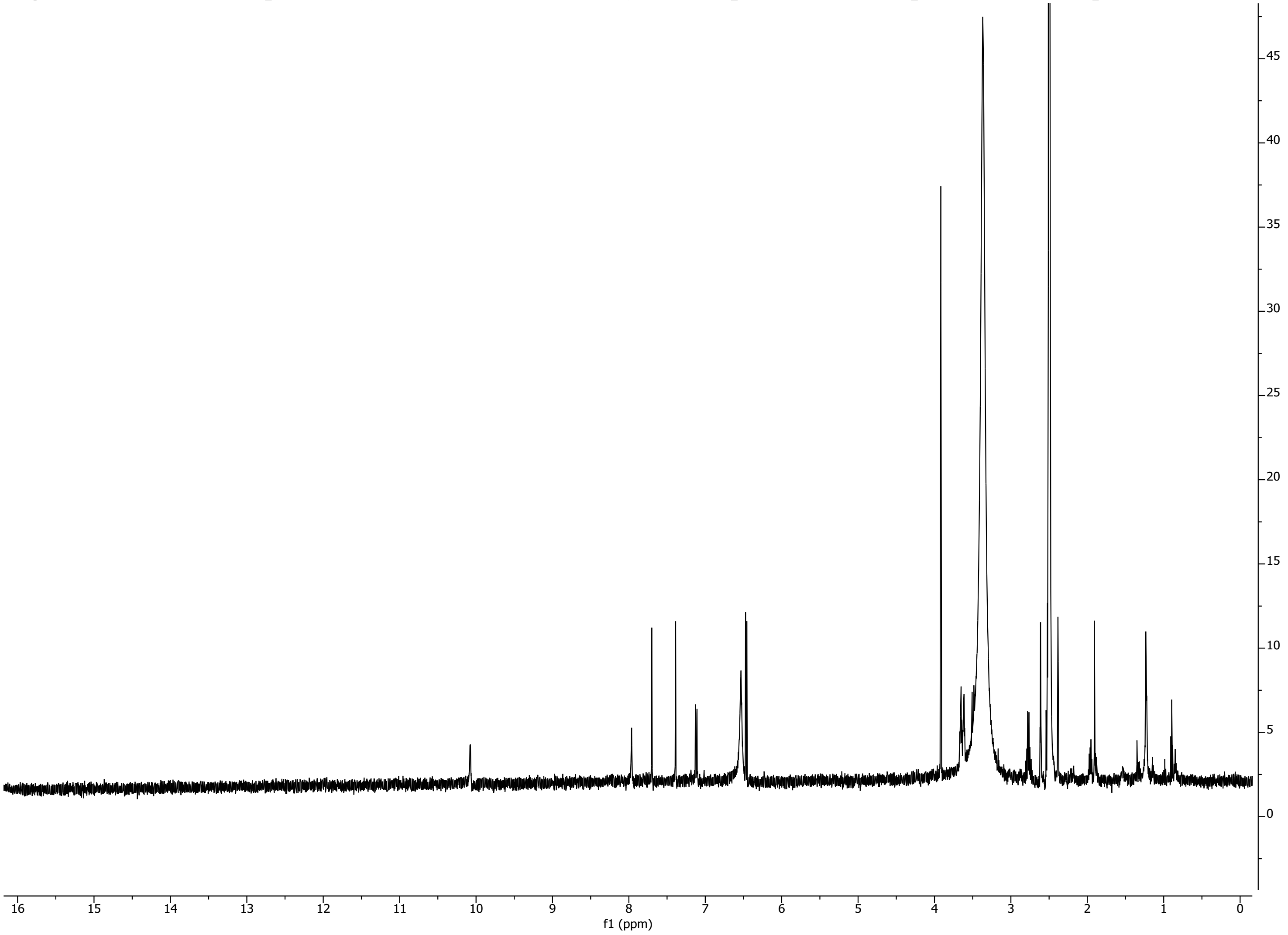

Figure S34. ^{1}H – ^{1}H COSY NMR spectrum (600.0 MHz, DMSO-d_6) for compound **23**. The spectrum was acquired over 8 scans at 256 t1 increments.

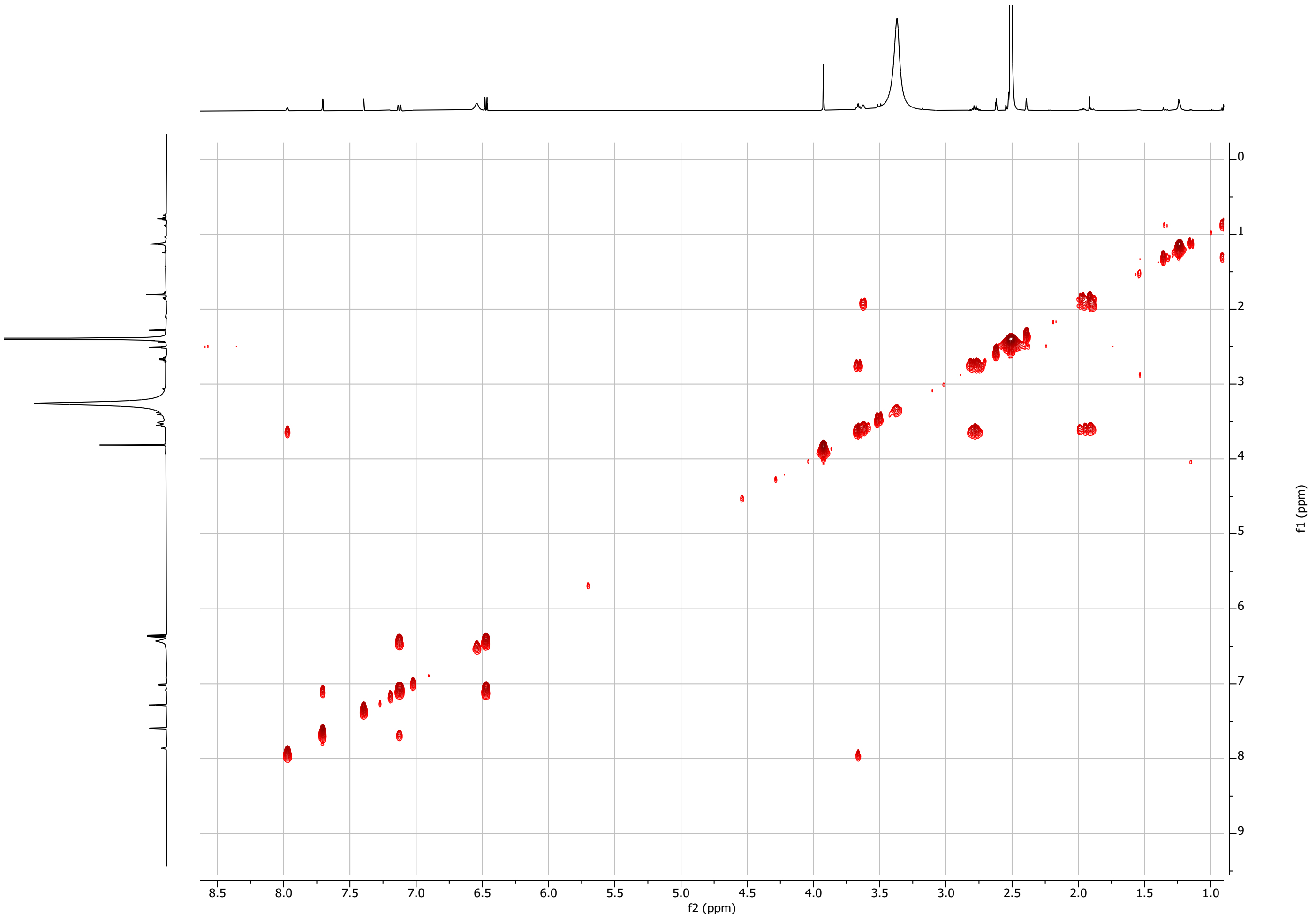

Figure S35. Multiplicity edited ^{1}H – ^{13}C HSQC NMR spectrum (600.0 MHz, DMSO-d_6) for compound **23**. The spectrum was acquired over 16 scans at 256 t1 increments.

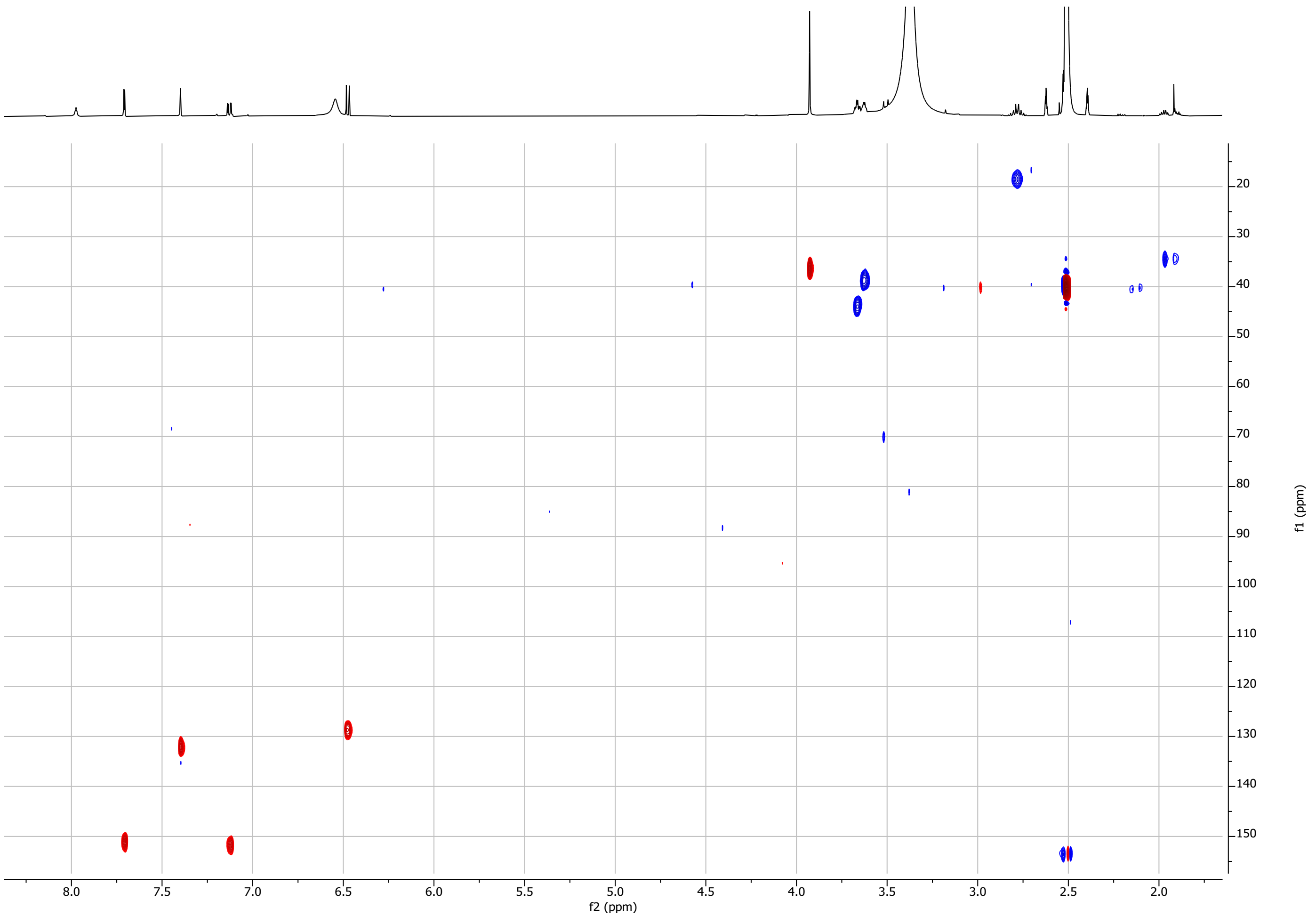

Figure S36. $^{1}H – ^{13}C$ HMBC NMR spectrum (600.0 MHz, DMSO-d_6) for compound **23**. The spectrum was acquired over 4000 scans at 128 t1 increments.

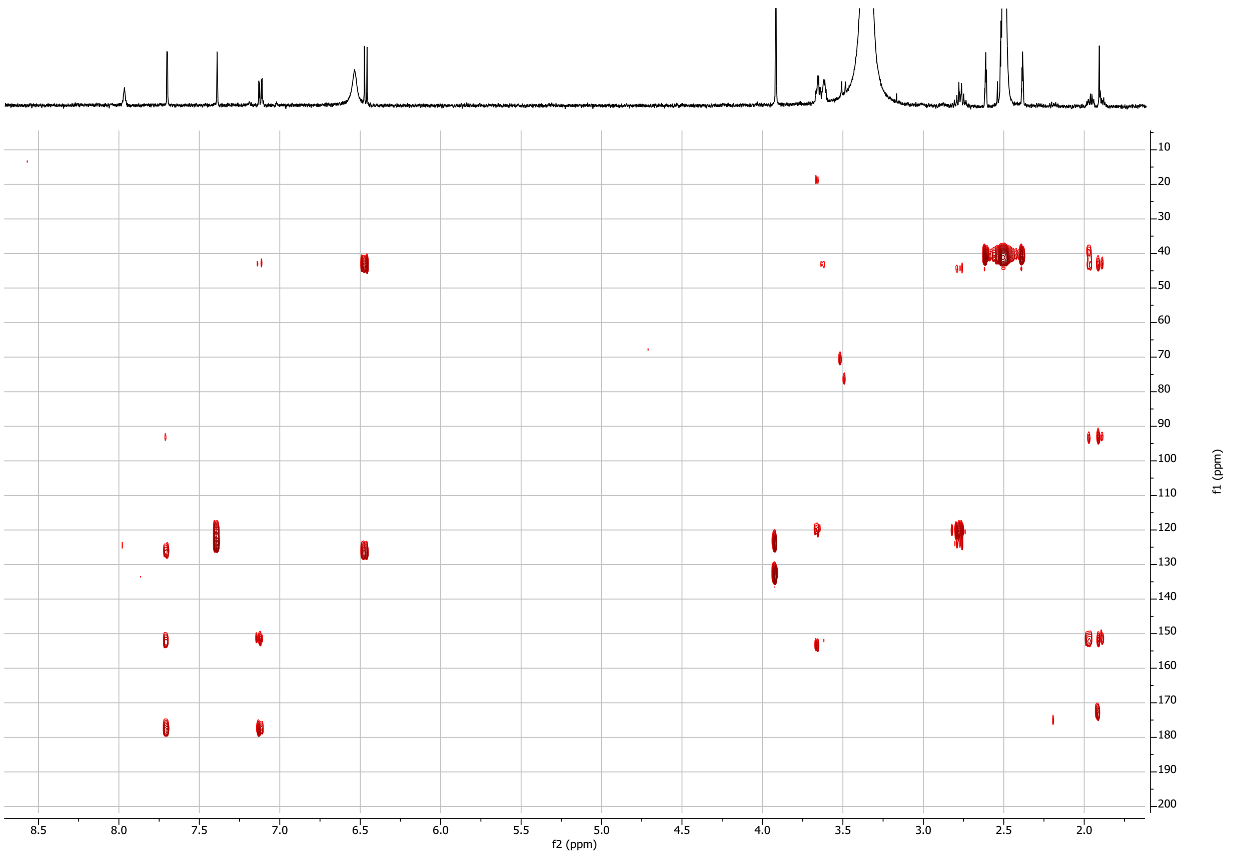

Figure S37. HRESIMS spectrum for compound **23**.

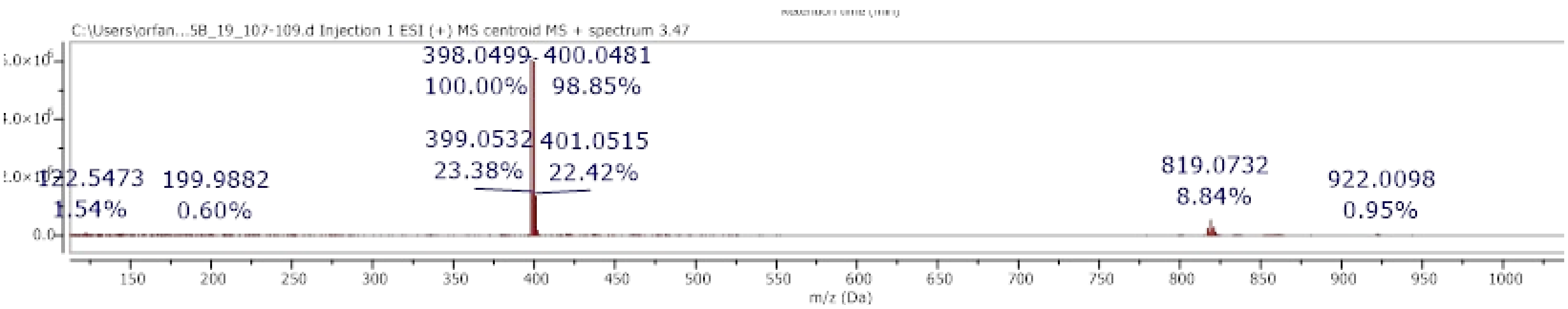

Figure S38. IR spectrum for compound **23**.

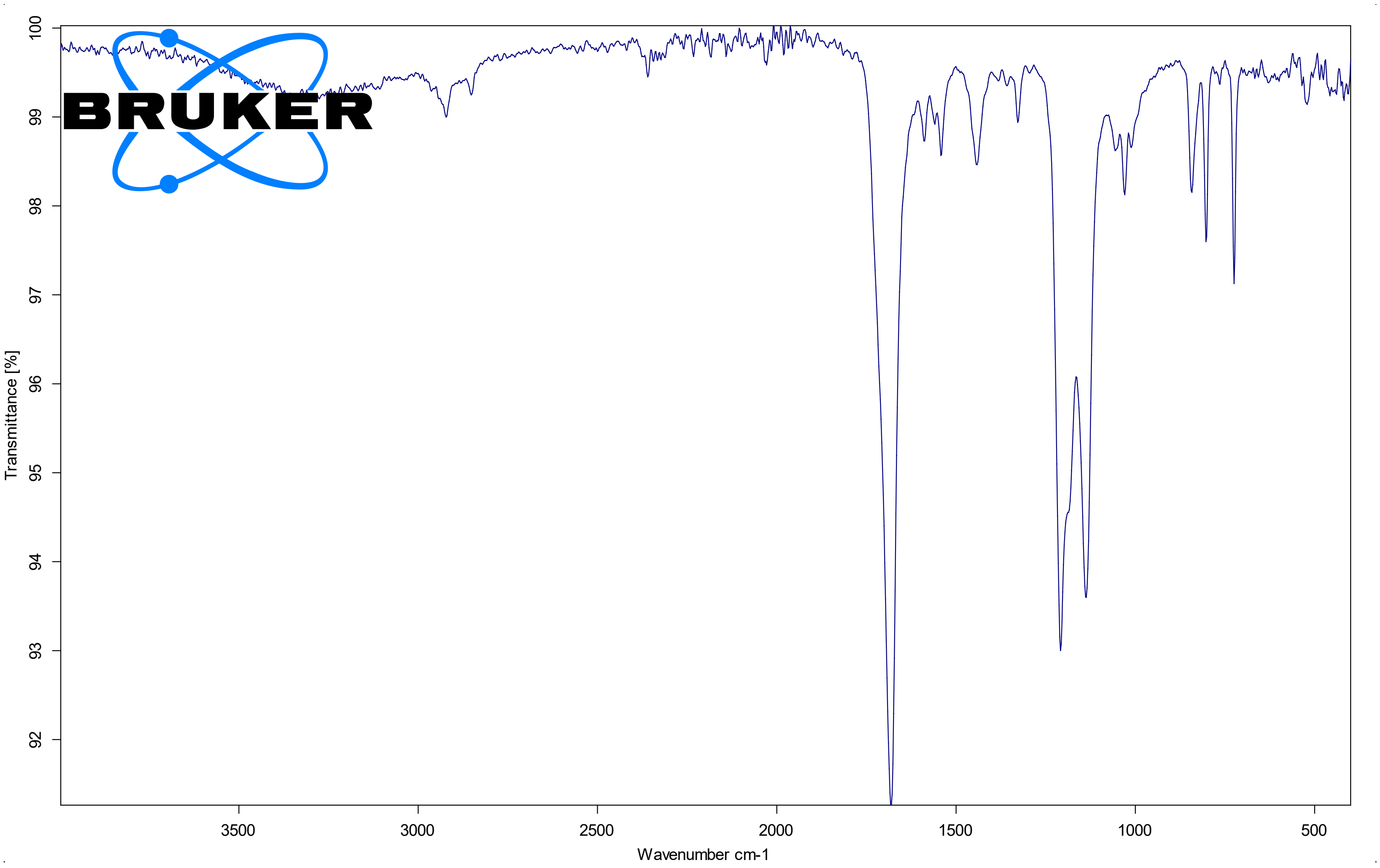

Figure S39: Activity of discorhabdins against MCC cell lines.
The indicated discorhabdins were assessed for their ability to affect survival of control (HaCaT) and Merkel cell carcinoma cell lines (VN-MCC: MCC13, MCC26, UISO and VP-MCC: MKL-1, MKL-2, Waga). Cells were plated at 2500 cells/well and compounds the next day. Cell survival was estimated by CellTiterGlo (normalized to untreated (DMSO) cells on the same plate). Structures were obtained from the supplier and/or the PubChem database or as described in the text. Number in bold in ( ) refers to structures in Figure 1 in the text. Curves were plotted and IC_{50} values and 95% confidence interval calculated by GraphPad software.

Discorhabdin A **(1)**

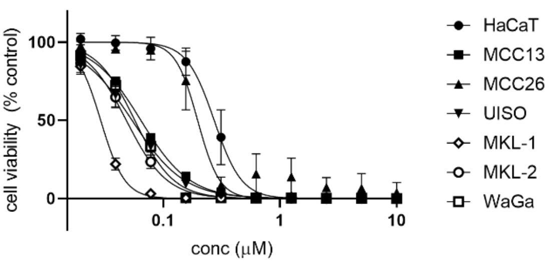

Cell Line	IC_{50} (nM)	95% CI
HaCaT	273	251-295
MCC13	64	63-66
MCC26	197	< 227
UISO	54	51-57
MKL-1	29	28-30
MKL-2	48	46-51
Waga	57	54-60

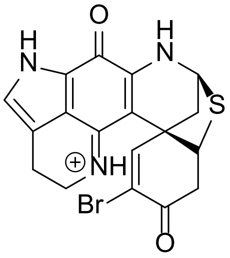

(+) Discorhabdin B **(2)**

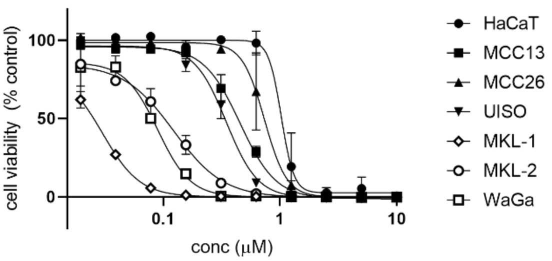

Cell Line	IC_{50} (nM)	95% CI
HaCaT	1040	> 917
MCC13	426	395-459
MCC26	731	< 805
UISO	331	306-357
MKL-1	25	24-25
MKL-2	90	78-104
Waga	75	65-86

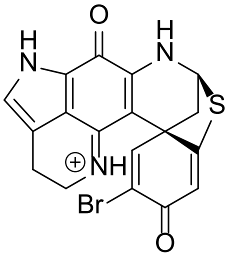

Discorhabdin C **(15)**

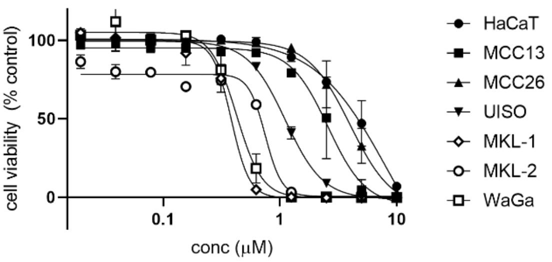

Cell Line	IC_{50} (nM)	95% CI
HaCaT	4240	3813-4708
MCC13	2297	1945-2685
MCC26	3674	3355-4023
UISO	1093	1051-1138
MKL-1	381	< 410
MKL-2	451	294-661
Waga	441	400-488

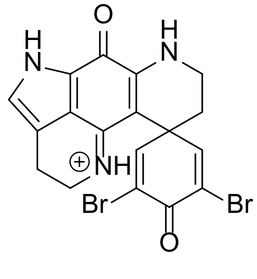

14-bromo discorhabdin C **(19)**

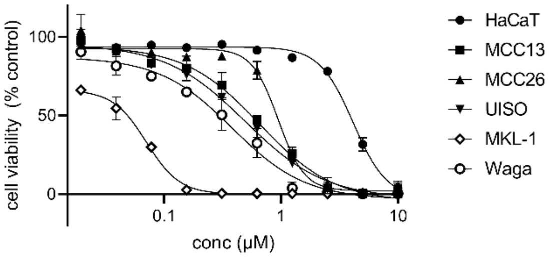

Cell Line	IC_{50} (nM)	95% CI
HaCaT	3739	3329-4184
MCC13	511	437-596
MCC26	893	775-1023
UISO	412	360-471
MKL-1	37.8	32.9-43.1
MKL-2	ND	
Waga	249	202-307

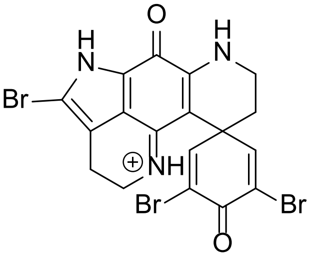

7,8-dehydro discorhabdin C **(20)**

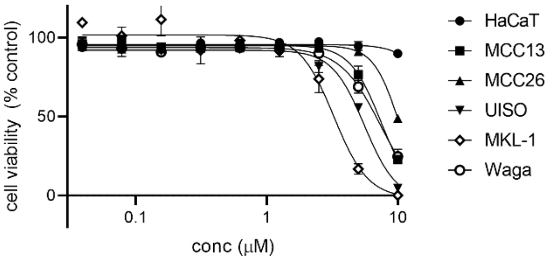

Cell Line	IC_{50} (nM)	95% CI
HaCaT	> 10,000	
MCC13	7478	7020-7963
MCC26	~ 10,000	
UISO	5567	5224-5967
MKL-1	3256	2804-3803
MKL-2	ND	
Waga	7429	6868-8116

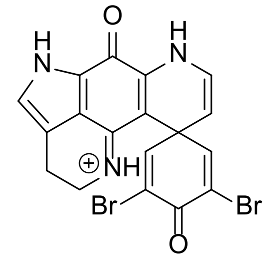

didebromo discorhabdin C **(21)**

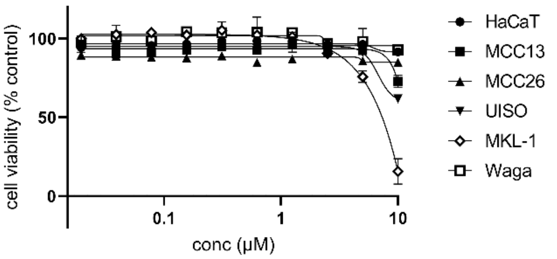

IC_{50} > 10 µM, all cell lines

(MKL-1, > 5 µM)

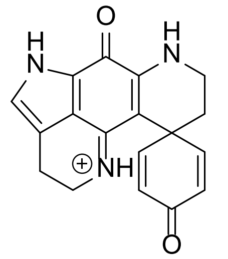

3-dihydro discorhabdin C **(16)**

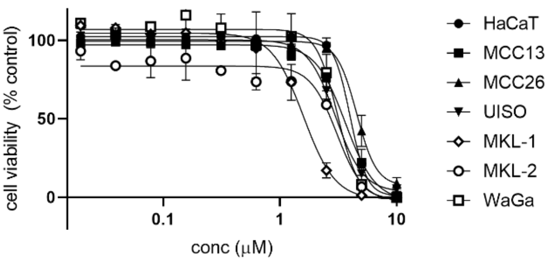

Cell Line	IC_{50} (nM)	95% CI
HaCaT	4132	> 3885
MCC13	3468	3254-3696
MCC26	4704	4471-4948
UISO	3086	2931-3253
MKL-1	1634	1490-1795
MKL-2	1935	1268-2799
Waga	3204	< 3847

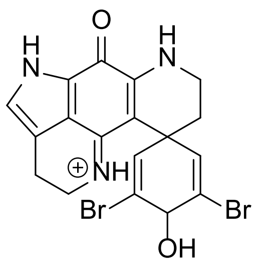

14-methyl discorhabdin C **(18)**

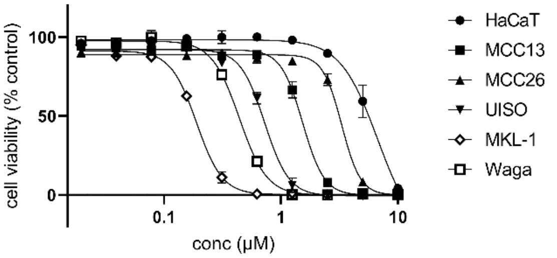

Cell Line	IC_{50} (nM)	95% CI
HaCaT	5381	5063-5717
MCC13	1451	1308-1609
MCC26	3063	2613-3575
UISO	670	598-743
MKL-1	175	161-191
MKL-2	ND	
Waga	430	413-448

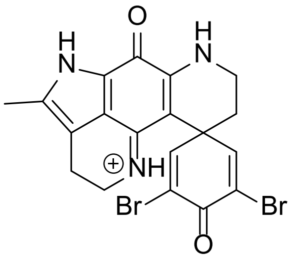

Discorhabdin C phenol **(24)**

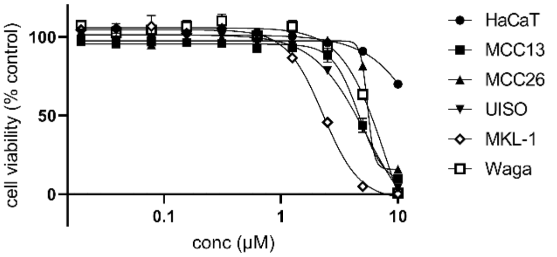

Cell Line	IC_{50} (nM)	95% CI
HaCaT	> 10,000	
MCC13	4642	4293-5016
MCC26	6920	6558-7304
UISO	4210	3978-4452
MKL-1	2333	2164-2508
MKL-2	ND	
Waga	5460	< 5972

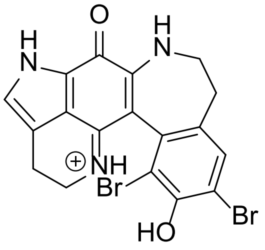

Discorhabdin D **(11)**

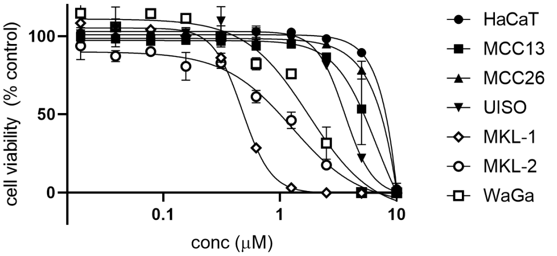

Cell Line	IC_{50} (nM)	95% CI
HaCaT	> 6000	-
MCC13	4995	4420-5557
MCC26	5853	-
UISO	3624	3343-3926
MKL-1	497	467-528
MKL-2	865	691-1073
Waga	1807	1466-2203

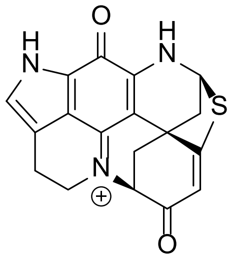

Discorhabdin E **(22)**

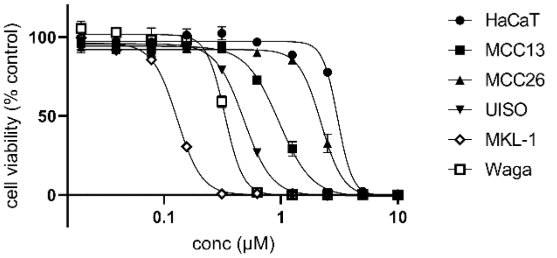

Cell Line	IC_{50} (nM)	95% CI
HaCaT	3006	< 3263
MCC13	888	812-969
MCC26	2002	1800-2253
UISO	459	434-485
MKL-1	125	119-130
MKL-2	ND	
Waga	332	< 347

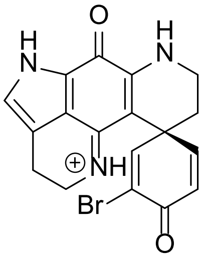

Methyl discorhabdin E **(23)**

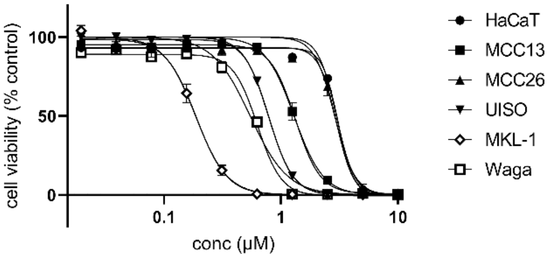

Cell Line	IC_{50} (nM)	95% CI
HaCaT	2981	< 3304
MCC13	1294	1209-1387
MCC26	2884	< 3177
UISO	781	761-803
MKL-1	186	176-198
MKL-2	ND	
Waga	546	472-625

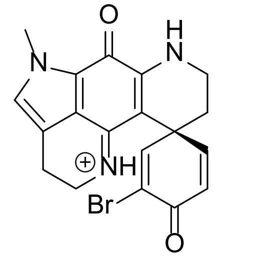

5-sulphonyl-7,8 dehydro-discorhabdin E **(9)**

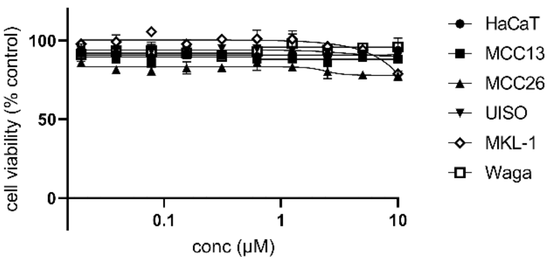

IC_{50} > 10 μM, all cell lines

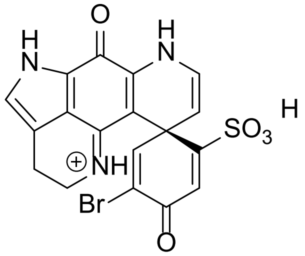

Discorhabdin G*/I **(4)**

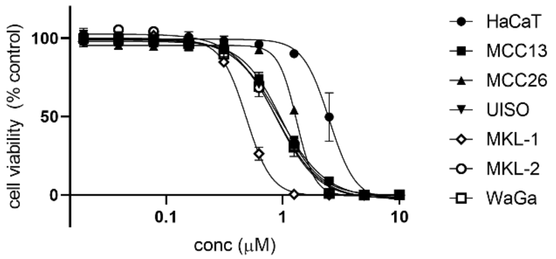

Cell Line	IC_{50} (nM)	95% CI
HaCaT	2449	2301-2591
MCC13	940	900-982
MCC26	1285	1235-1337
UISO	921	888-955
MKL-1	481	469-493
MKL-2	879	817-946
Waga	859	810-911

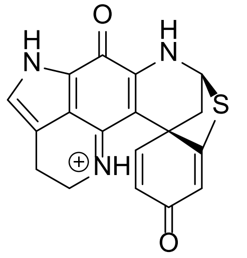

5-thiomethyl discorhabdin G*/I **(7)**

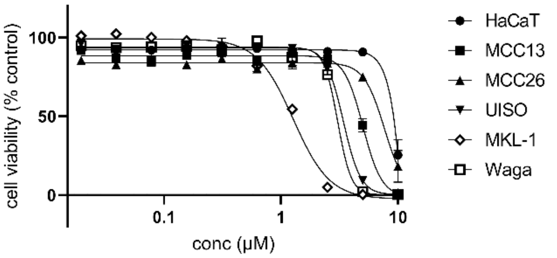

Cell Line	IC_{50} (nM)	95% CI
HaCaT	7963	> 7021
MCC13	4503	3694-5364
MCC26	7891	> 3916
UISO	3269	2937-3594
MKL-1	1230	1138-1326
MKL-2	ND	
Waga	2986	< 3336

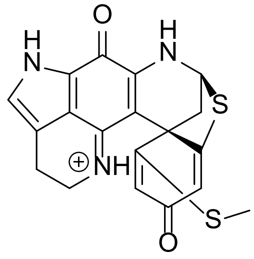

Discorhabdin H **(14)**

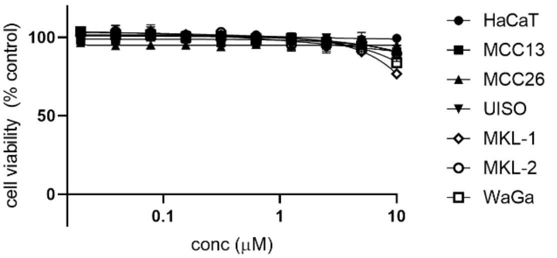

IC_{50} > 10 µM, all cell lines

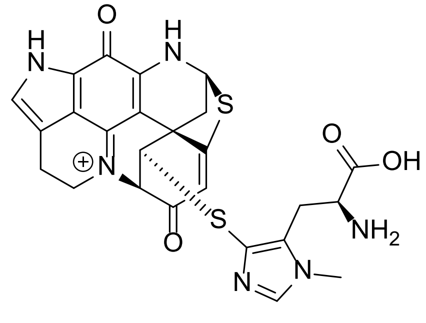

Discorhabdin K **(5)**

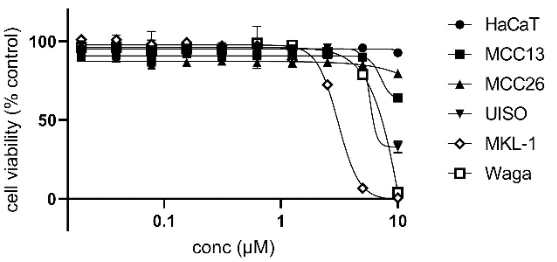

Cell Line	IC_{50} (nM)	95% CI
HaCaT	> 10,000	
MCC13	> 10,000	
MCC26	> 10,000	
UISO	8300	7650-8970
MKL-1	3026	2833-3241
MKL-2	ND	
Waga	6152	< 6770

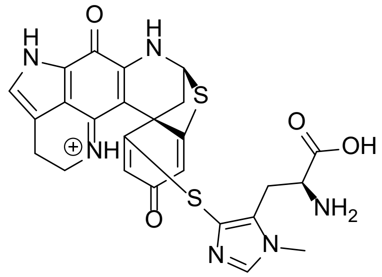

Discorhabdin K methyl ester **(6)**

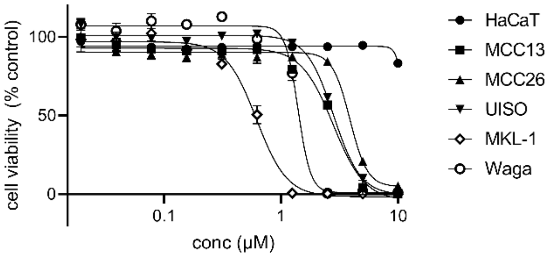

Cell Line	IC_{50} (nM)	95% CI
HaCaT	> 10,000	
MCC13	2442	2093-2813
MCC26	3649	3107-4270
UISO	2821	2693-2961
MKL-1	587	535-641
MKL-2	ND	
Waga	1431	< 1662

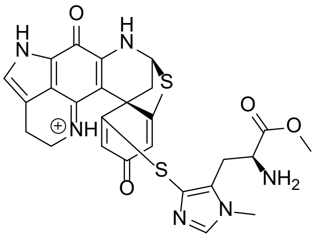

Discorhabdin L **(12)**

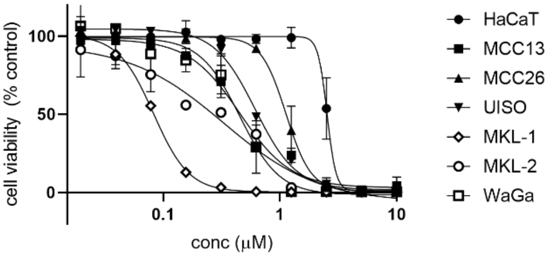

Cell Line	IC_{50} (nM)	95% CI
HaCaT	2554	2424-2734
MCC13	466	609-531
MCC26	1131	1046-1218
UISO	660	611-715
MKL-1	83	80-87
MKL-2	245	189-316
Waga	440	366-523

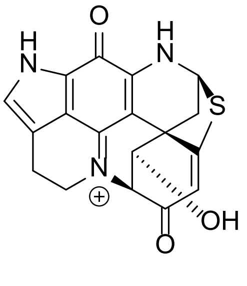

Discorhabdin N **(13)**

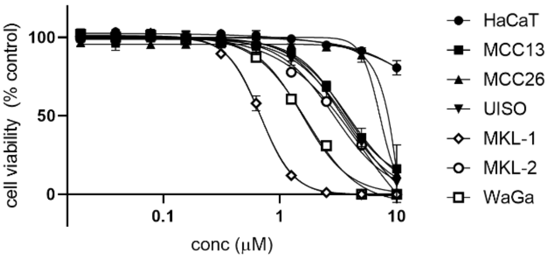

Cell Line	IC_{50} (nM)	95% CI
HaCaT	> 10,000	-
MCC13	3815	3568-4081
MCC26	> 6000	-
UISO	3417	3216-3631
MKL-1	682	661-703
MKL-2	2912	2678-3165
Waga	1564	1492-1661

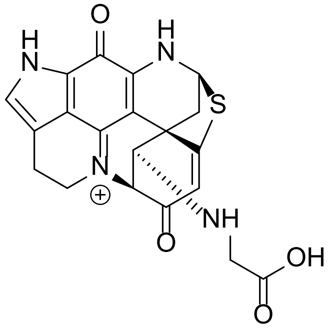

Discorhabdin P **(17)**

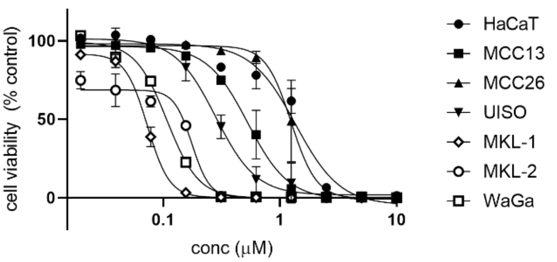

Cell Line	IC_{50} (nM)	95% CI
HaCaT	1260	1059-1477
MCC13	499	454-547
MCC26	1210	1080-1344
UISO	292	265-323
MKL-1	68	64-71
MKL-2	87	65-113
Waga	106	101-113

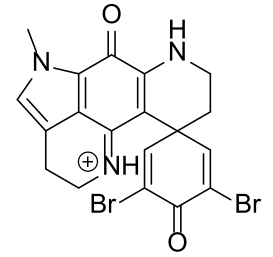

Discorhabdin Q **(3)**

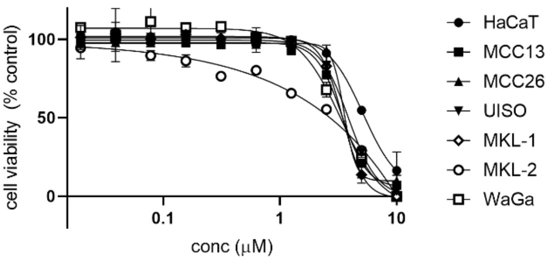

Cell Line	IC_{50} (nM)	95% CI
HaCaT	5446	5062-5868
MCC13	3495	3323-3675
MCC26	3762	3483-4154
UISO	3849	3676-4027
MKL-1	3441	3208-3692
MKL-2	2039	1545-2671
Waga	3293	2859-3803

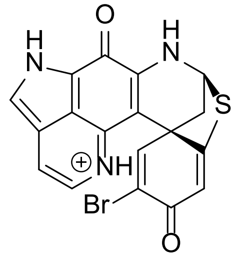

N-13-demethyl discorhabdin U **(8)**

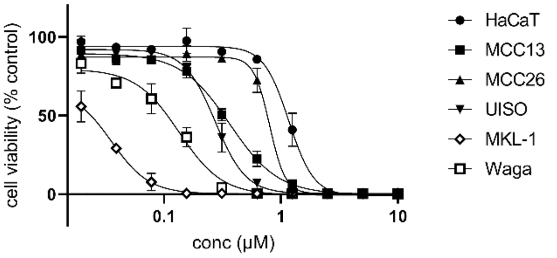

Cell Line	IC_{50} (nM)	95% CI
HaCaT	1009	982-1201
MCC13	299	262-339
MCC26	749	< 870
UISO	251	228-275
MKL-1	22.7	20.8-24.5
MKL-2	ND	
Waga	86.9	73.3-102.4

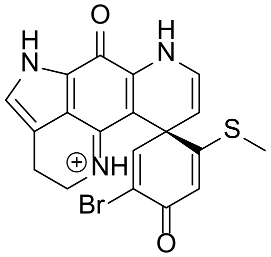

Discorhabdin W **(10)**

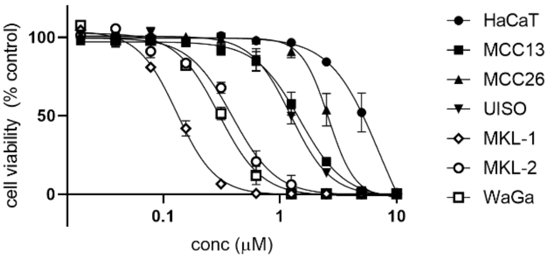

Cell Line	IC_{50} (nM)	95% CI
HaCaT	4865	4470-5264
MCC13	1384	1285-1489
MCC26	2560	2439-2682
UISO	1243	1181-1307
MKL-1	136	130-143
MKL-2	387	350-426
Waga	307	284-333

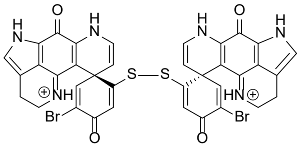

Table S3: Activity of discorhabdins against MCC cell lines (area under the curve).
Areas under the dose-response curves (AUC) shown in Figure S1 were calculated by GraphPad software. These values were used to generate Figures 2 and 3 in the main text.

		AUC (arbitrary units)						
Discorhabdin	#	95% CI						
		HaCaT	MCC13	MCC26	UISO	MKL-1	MKL-2	WaGa
A	1	29.87	8.589	88.19	7.172	4.944	6.99	6.873
		23.62 to 36.11	7.081 to 10.10	22.43 to 154.0	6.455 to 7.890	2.955 to 6.933	6.261 to 7.719	6.146 to 7.601
(+)B	2	133.9	51.68	83.94	39.51	3.635	14.49	9.624
		83.13 to 184.7	47.54 to 55.82	66.10 to 101.8	35.10 to 43.93	2.216 to 5.053	12.29 to 16.69	8.275 to 10.97
(-)B		114.3	41.45	80.18	33.26	1.929	9.451	5.814
		78.29 to 150.4	38.91 to 43.98	74.46 to 85.89	28.58 to 37.93	0.4524 to 3.406	8.591 to 10.31	3.746 to 7.882
C	15	507.2	272.6	446.4	137.9	42.87	72.69	51.37
		427.5 to 586.9	192.4 to 352.8	372.4 to 520.4	131.7 to 144.1	39.20 to 46.54	54.47 to 90.92	44.10 to 58.64
14-bromo C	19	441.7	90.22	120.6	88.25	7.189	ND	50.51
		413.8 to 469.6	78.20 to 102.2	106.0 to 135.3	64.78 to 111.7	6.646 to 7.732		41.00 to 60.03
7,8 dehydro C	20	940.1	694.4	815.8	542.4	376.3	ND	661
		924.1 to 956.2	664.7 to 724.1	806.3 to 825.3	523.6 to 561.3	346.0 to 406.6		628.8 to 693.2
didebromo C	21	952.2	883	866.1	851.9	682.4	ND	973.8
		944.7 to 959.7	857.7 to 908.3	859.9 to 872.3	838.2 to 865.6	638.1 to 726.8		927.6 to 1020
3-dihydro C	16	454.5	404	543.3	375.8	195.9	277.4	370.5
		434.2 to 474.7	358.4 to 449.7	485.4 to 601.1	349.6 to 402.1	172.3 to 219.5	265.1 to 289.7	326.9 to 414.2
14-methyl C	18	584.7	159.8	329.4	74.19	20.95	ND	51.75
		527.1 to 642.2	152.4 to 167.1	318.7 to 340.2	67.32 to 81.06	17.61 to 24.28		47.44 to 56.05
C phenol	24	884.9	528.6	708.7	493.2	279.7	ND	611.2
		880.0 to 889.8	501.1 to 556.1	695.1 to 722.4	485.5 to 501.0	275.4 to 283.9		599.1 to 623.3
D	11	715.8	540.6	653.2	423.9	60.49	143.2	216.6
		696.8 to 734.8	415.6 to 665.6	621.8 to 684.7	413.0 to 434.7	58.60 to 62.38	135.0 to 151.4	188.2 to 244.9
E	22	328.3	106.9	226.2	53.3	14.16	ND	36.75
		315.7 to 340.9	99.94 to 113.8	210.0 to 242.3	51.20 to 55.39	11.58 to 16.74		34.42 to 39.07
13-methyl E	23	319.6	155.7	306.3	90.78	23.33	ND	65.1
		300.8 to 338.5	147.9 to 163.4	284.9 to 327.7	88.22 to 93.33	21.70 to 24.97		59.80 to 70.41

5-sulphonyl-7-8 dehydro E	9	911.5	882.5	793.9	914.8	917.6	ND	949.7
		889.2 to 933.7	865.6 to 899.4	770.7 to 817.0	909.0 to 920.6	902.0 to 933.1		920.7 to 978.7
G*/I	4	267.7	124.8	140	121.7	54.17	108.8	105.3
		225.2 to 310.1	118.7 to 130.5	136.4 to 143.6	117.1 to 126.3	51.05 to 56.80	102.4 to 115.1	95.51 to 114.8
1-thiomethyl G*/I	7	745.5	495.1	638.2	360.8	144.5	ND	321.6
		697.7 to 793.4	470.7 to 519.5	587.9 to 688.5	347.1 to 374.5	140.8 to 148.3		311.3 to 331.9
H	14	992.5	950.6	947	952.3	901.7	946.5	930
		980.4 to 1005	904.3 to 996.8	934.1 to 959.9	920.5 to 984.1	882.8 to 920.6	910.0 to 982.9	908.7 to 951.4
K	5	944.5	837.7	841.3	767.7	335	ND	660.5
		938.4 to 950.7	824.3 to 851.1	828.5 to 854.1	739.0 to 796.3	327.8 to 342.2		641.3 to 679.7
K methyl ester	6	912.2	281.5	411.6	335.9	65.12	ND	179.3
		897.5 to 926.9	256.7 to 306.3	398.3 to 424.9	329.1 to 342.8	61.08 to 69.17		163.6 to 194.9
L	12	290.4	93.66	140.6	103	13.23	50.71	55.36
		235.0 to 345.8	63.76 to 123.6	117.0 to 164.3	95.62 to 110.4	12.80 to 13.66	45.75 to 55.67	42.95 to 67.76
N	13	909	478.7	719.3	436.7	83.43	391.1	200.7
		883.1 to 934.8	443.4 to 514.0	625.9 to 812.6	410.5 to 462.9	79.87 to 86.99	377.9 to 404.4	191.6 to 209.8
P	17	150.9	64.76	140.1	50.65	7.146	17.05	14.11
		137.0 to 164.8	53.33 to 76.20	104.3 to 175.9	31.81 to 69.48	6.500 to 7.792	13.51 to 20.59	13.20 to 15.01
Q	3	604.2	419.1	432.3	447.9	389.4	350.1	405.1
		543.4 to 665.0	408.8 to 429.3	411.5 to 453.1	427.2 to 468.6	352.0 to 426.8	342.4 to 357.7	384.1 to 426.0
N-13-demethyl U	8	123.3	48.65	80.16	32.84	5.221	ND	14.83
		108.7 to 137.9	44.91 to 52.39	73.53 to 86.79	29.50 to 36.18	4.608 to 5.835		13.17 to 16.48
W	10	538.3	184.2	290.1	162.4	18.4	55.04	40.16
		470.2 to 606.4	166.2 to 202.2	261.3 to 319.0	152.9 to 171.9	17.50 to 19.30	45.37 to 64.71	35.84 to 44.49

Figure S40: Activity of discorhabdins against MCC cell lines – comparison of IC_{50} and AUC IC_{50} and AUC values from Supplementary Figure S1 and Supplementary Table S1 respectively had a linear relationship. Best line and correlation coefficient were calculated by Excel (IC_{50} > 7.5 μM excluded).

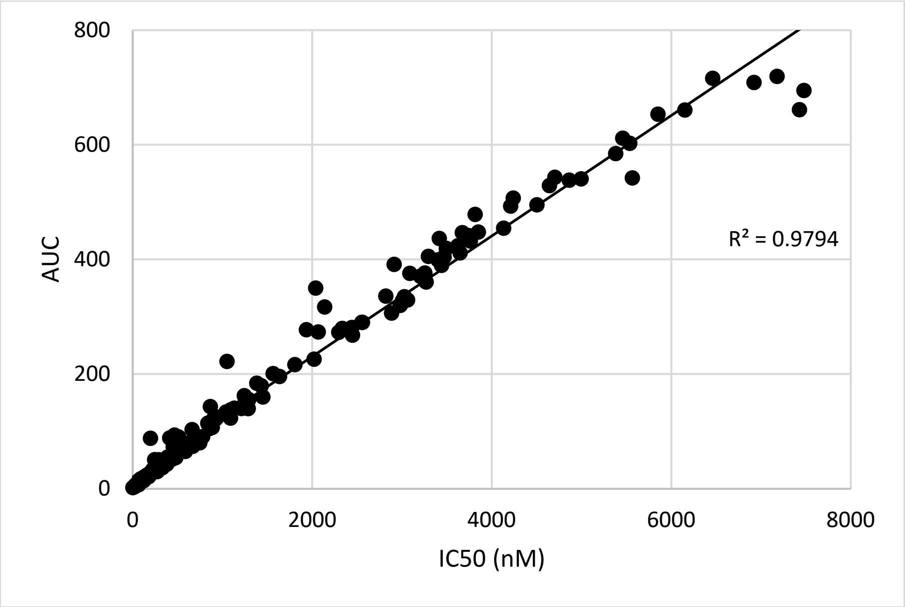

Figure S41: Discorhabdin selectivity

Discorhabdin selectivity for VP-MCC and VN-MCC cells was estimated by comparison of activity (average AUC) for each discorhabdin against VP-MCC (panel A) or VN-MCC (panel B) cell lines compared to control cells. Discorhabdins were sorted by selectivity from most to least selective. -fold difference = (ave VN-MCC or VP-MCC AUC/control AUC). Error bars represent sd or range.

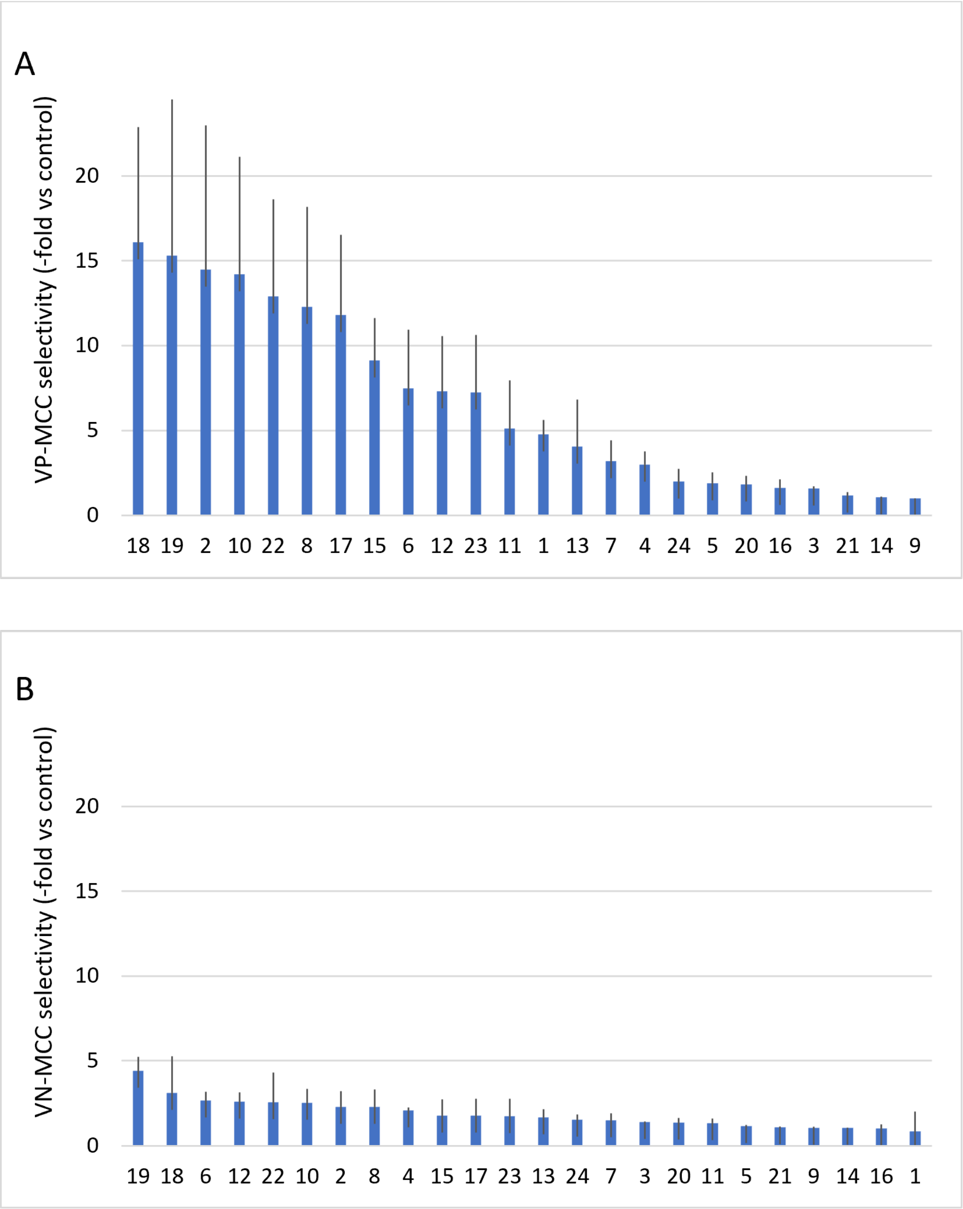

Figure S42: Caspase activation in response to discorhabdin treatment.
Cells were treated for 8-24 h with the indicated discorhabdins or bortezomib (bort) at 1 μM followed by estimation of caspase activity (Promega CaspaseGLO kit). Data normalized to DMSO control. Error bars represent sd (n = 4).

$p < 0.001$ for 24 h bortezomib for all cell lines (students t-test). Discorhabdin effects were insignificant.

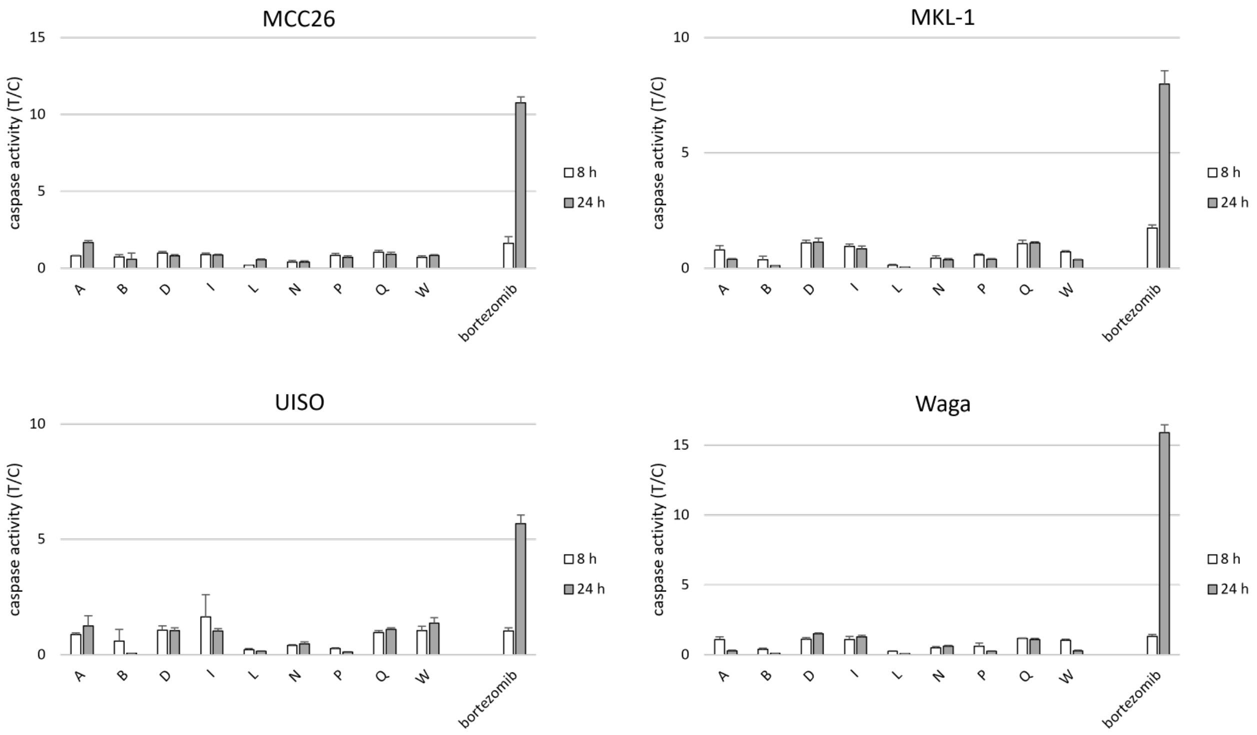

Figure S43: Apoptotic/necrotic signals in MCC cells in response to discorhabdins
Cells were treated for up to 24 h with the indicated discorhabdins while monitoring of Annexin V (LUM signal) and cell permeability (FL signal) using the Promega RealTimeGlo^{TM} Apoptosis and Necrosis assay kit. T/T0 = -fold increase over baseline. Error bars not included for clarity (n = 3).

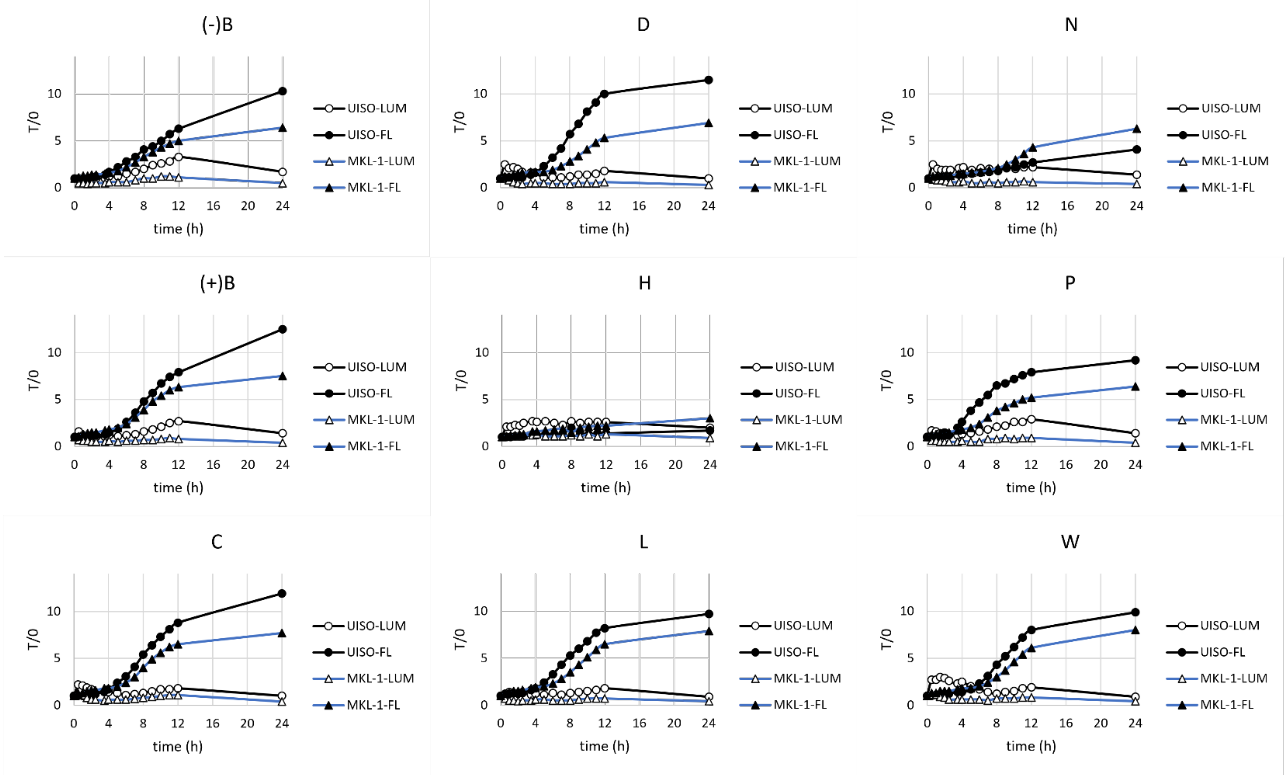

Figure S44: Effect of caspase inhibitor on MCC cell response to discorhabdins
Cells were pre-treated for 1 h ± ZVAD-FMK (40 μM) then 12-24 h with the indicated discorhabdins (10 μM) or bortezomib (bort, 1 μM) in the continued presence of ZVAD followed by estimation of cell survival (Promega MT Cell Viability kit). Data normalized to DMSO or ZVAD only controls. Error bars represent sd (n =3) for discorhabdins, range (n = 2) for bortezomib.

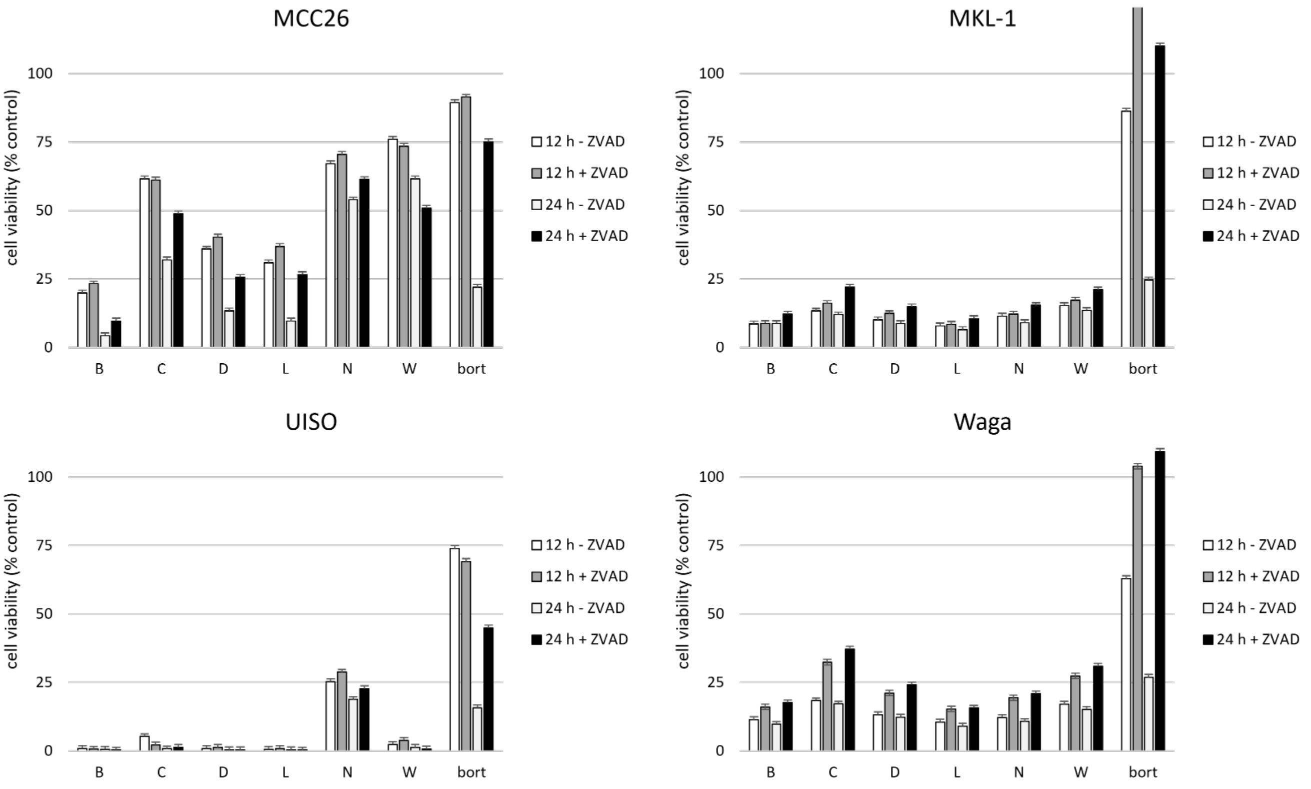

Figure S45: Effects of discorhabdins and bortezomib on MCC cell reductive potential Indicated MCC cell lines were treated with discorhabdins or Bortezomb for 0-24 h with monitoring of reduction potential signal (Promega RealTimeGlo MT Cell Viability kit). Signals were normalized to DMSO control. Error bars not included for clarity (n = 3).

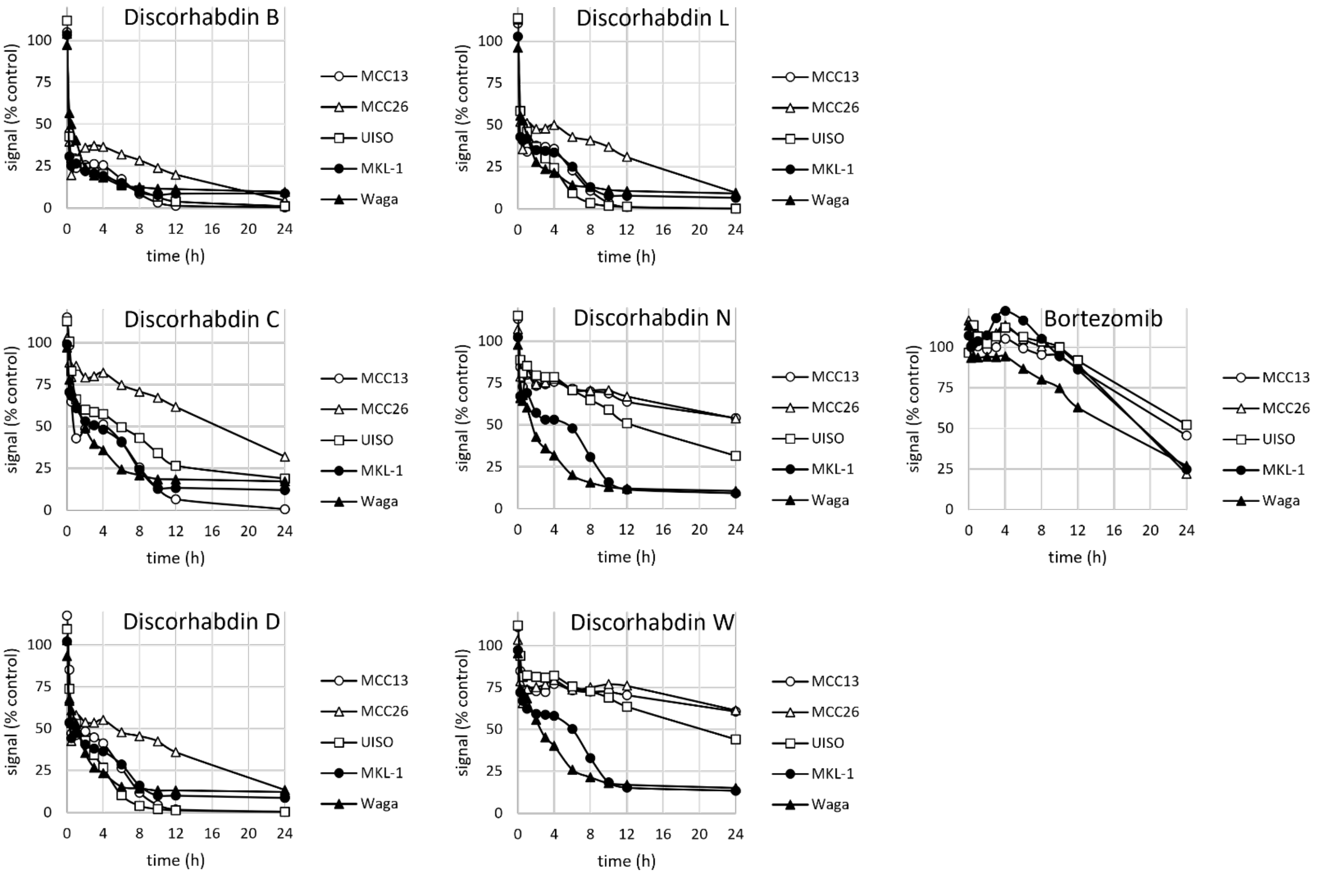

Figure S46: Effects of discorhabdins, doxorubicin (DOX) and bortezomib on MCC cell mitochondrial potential

The VN-MCC and VP-MCC cell lines UISO and MKL-1 respectively were treated for 2 or 24 h with the indicated compound and assessed for mitochondrial membrane potential (MMP) using JC-10 as a probe (added for the last hour of incubation). Red/green fluorescence ratio was normalized to DMSO control. Error bars represent se (n = 3) or range (n = 2 for DOX only). Additional time points appear in Figure 6 for discorhabdins B, C, and L.

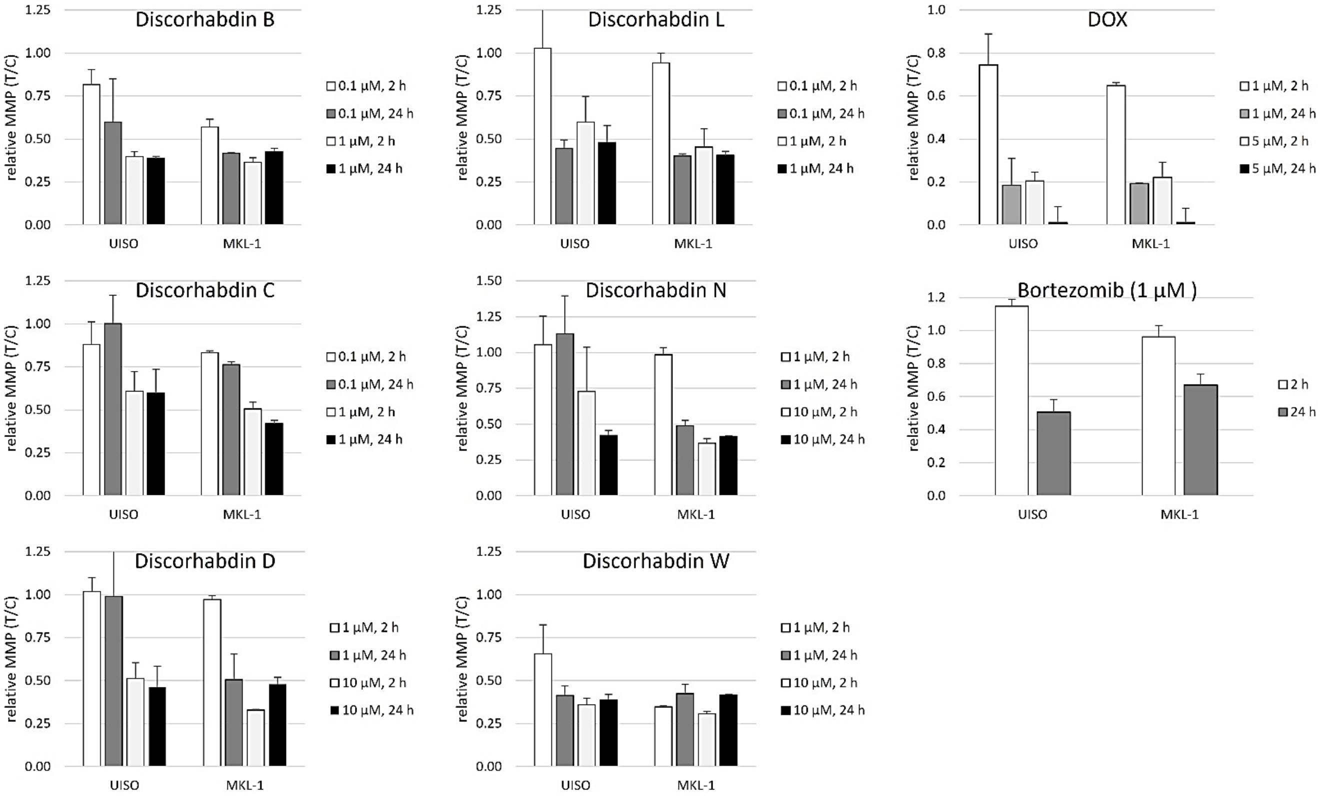

Figure S47: Discorhabdins do not induce ROS generation in MCC cells
MCC cells were treated with discorhabdins (10 µM) or with TBHP (0.1 mM) and ROS generation monitored by measuring DCFDA signal at intervals over 4 h. Signals were normalized to DMSO control in the same cell line/same time point. Error bars (n = 3) not shown for clarity.

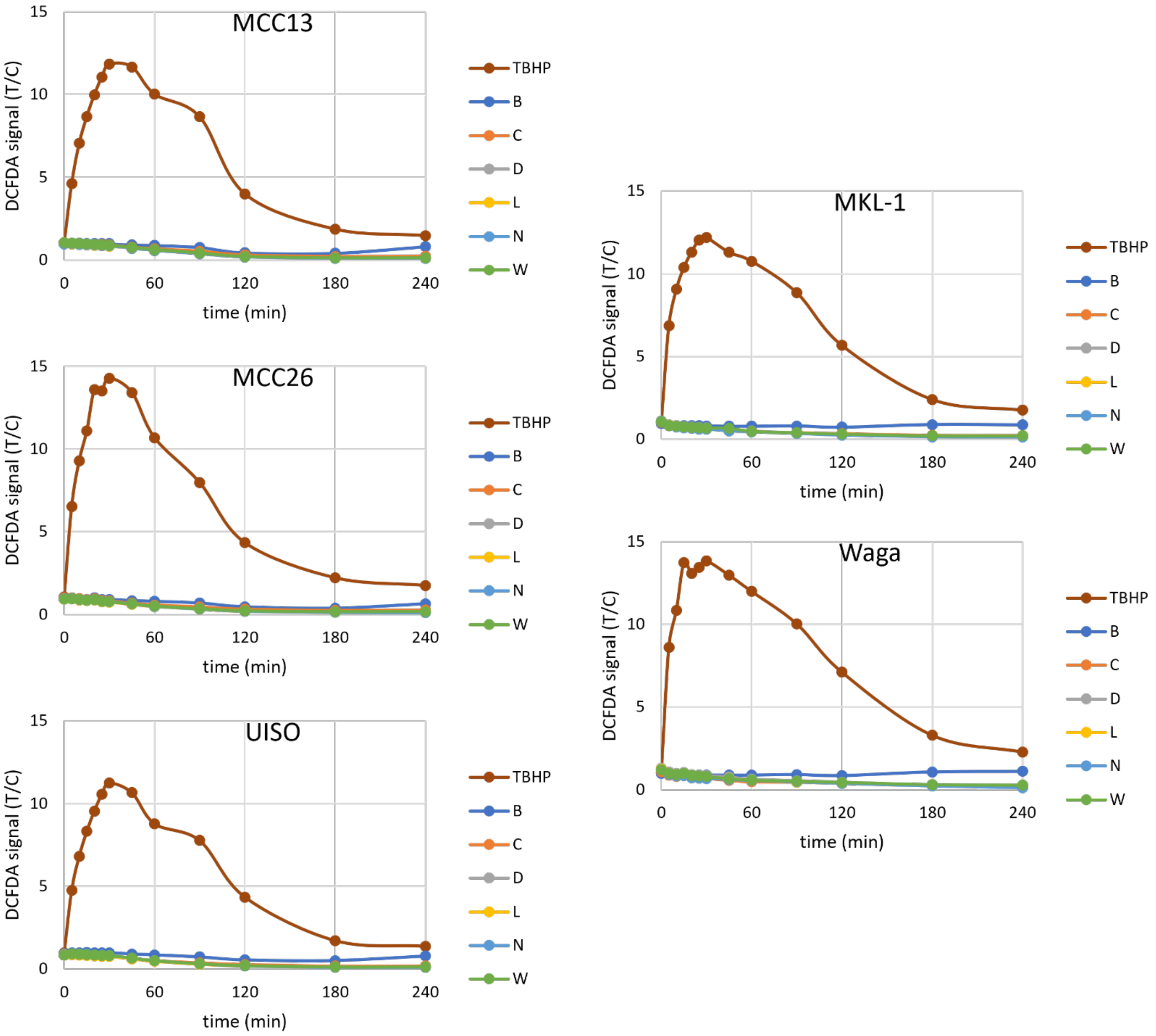

Figure S48: Discorhabdins do not increase intracellular calcium in MCC cells
MCC cells were treated with discorhabdins at 10 µM or with thapsigargin (20 µM) and calcium mobilization monitored by measuring Fluo-4 signal at intervals over 30 min and normalized to control (DMSO) at the same time point, same cells. Error bars (n = 3) not included for clarity.

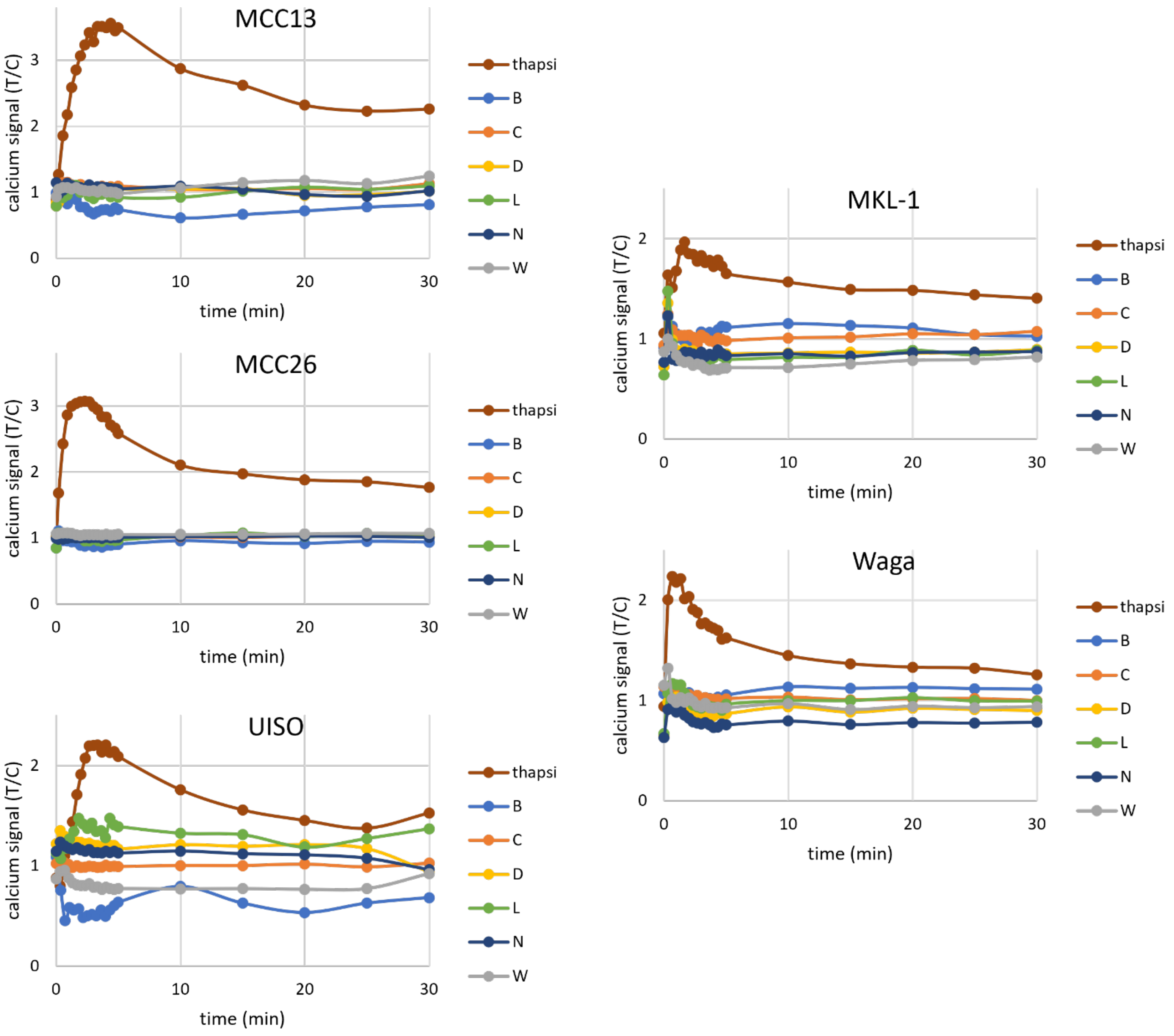

Supplement: Supplementary file 1 [file marinedrugs-21-00474-s001.zip › marinedrugs-2556743-supplementary.pdf]
